# Hepialiamides A–C: Aminated Fusaric Acid Derivatives and Related Metabolites with Anti-Inflammatory Activity from the Deep-Sea-Derived Fungus *Samsoniella hepiali* W7

**DOI:** 10.3390/md21110596

**Published:** 2023-11-16

**Authors:** Zheng-Biao Zou, Tai-Zong Wu, Long-He Yang, Xi-Wen He, Wen-Ya Liu, Kai Zhang, Chun-Lan Xie, Ming-Min Xie, Yong Zhang, Xian-Wen Yang, Jun-Song Wang

**Affiliations:** 1 Center for Molecular Metabolism, School of Environmental and Biological Engineering, Nanjing University of Science and Technology, 200 Xiaolingwei Street, Nanjing 210094, China; zhengbiaozou@njust.edu.cn (Z.-B.Z.); wenyaliu2015@163.com (W.-Y.L.); 2 Key Laboratory of Marine Genetic Resources, Third Institute of Oceanography, Ministry of Natural Resources, 184 Daxue Road, Xiamen 361005, China; wutaizong@tio.org.cn (T.-Z.W.); z18252730063@163.com (K.Z.); xiechunlanxx@163.com (C.-L.X.); xiemingmin@tio.org.cn (M.-M.X.); zhangyong@tio.org.cn (Y.Z.); 3 Technical Innovation Center for Utilization of Marine Biological Resources, Third Institute of Oceanography, Ministry of Natural Resources, 184 Daxue Road, Xiamen 361005, China; longheyang@tio.org.cn (L.-H.Y.); hexiwen1224@163.com (X.-W.H.)

**Keywords:** deep-sea-derived fungus, fusaric acid derivatives, GNPS molecular networking, inflammation, nitric oxide production

## Abstract

A systematic investigation combined with a Global Natural Products Social (GNPS) molecular networking approach, was conducted on the metabolites of the deep-sea-derived fungus *Samsoniella hepiali* W7, leading to the isolation of three new fusaric acid derivatives, hepialiamides A–C (**1**–**3**) and one novel hybrid polyketide hepialide (**4**), together with 18 known miscellaneous compounds (**5**–**22**). The structures of the new compounds were elucidated through detailed spectroscopic analysis. as well as TD-DFT-based ECD calculation. All isolates were tested for anti-inflammatory activity in vitro. Under a concentration of 1 µM, compounds **8**, **11**, **13**, **21**, and **22** showed potent inhibitory activity against nitric oxide production in lipopolysaccharide (LPS)-activated BV-2 microglia cells, with inhibition rates of 34.2%, 30.7%, 32.9%, 38.6%, and 58.2%, respectively. Of particularly note is compound **22,** which exhibited the most remarkable inhibitory activity, with an IC_50_ value of 426.2 nM.

## 1. Introduction

Secondary metabolites produced by marine-derived fungi have been proven to possess a broad spectrum of bioactivities, such as cytotoxicity, antibacterial, antioxidant, antimalarial, anti-inflammatory, and antiviral properties and have been recognized as significant chemical entities for drug discovery [1,2,3,4,5,6,7,8]. However, continuous investigations on secondary metabolites from marine-derived fungi have led to a high frequency of rediscovery of known compounds, for which, dereplication becomes critical in microbial biodiscovery. Mass spectrometry-based GNPS molecular networking (https://gnps.ucsd.edu, accessed on 1 January 2016) is a strategy that has been proven to be a powerful and promising tool for dereplication. GNPS uses an untargeted metabolomics approach that powerfully processes tandem mass spectrometry (MS/MS) fragmentation data. It is a vector-based workflow that calculates cosine scores (between 0 and 1) to determine the degree of similarity between MS^2^ fragments. These fragment ions (nodes) are then organized into relational networks depending on their similarity [9,10,11]. GNPS has been widely used to identify known compounds and potential new analogs, greatly speeding up the dereplication and structure-based discovery of natural products [12,13,14].

Fusaric acid (FA), featuring a picolinic acid core, is a well-known mycotoxin commonly found in the metabolites of numerous *Fusarium* species. The biosynthesis of FA and its related analogues has attracted attention since the last century due to the intriguing pyridine moiety, which has been proven to start from an aspartic acid precursor and malonyl-CoA [15,16,17,18]. However, naturally occurring amidated derivatives of FA have not been well-described yet in terms of their structural diversity and biological activity, with only two examples reported so far, including atransfusarin from the endophytic fungus *Alternaria atrans* MP-7 [19] and 2-(4-butylpicolinamide) acetic acid from *Fusarium fujikuroi* [20]. Atransfusarin was documented with mild anti-fungi activity while 2-(4-butylpicolinamide) acetic acid was inactive against the tested pathogens.

**Figure 1:**
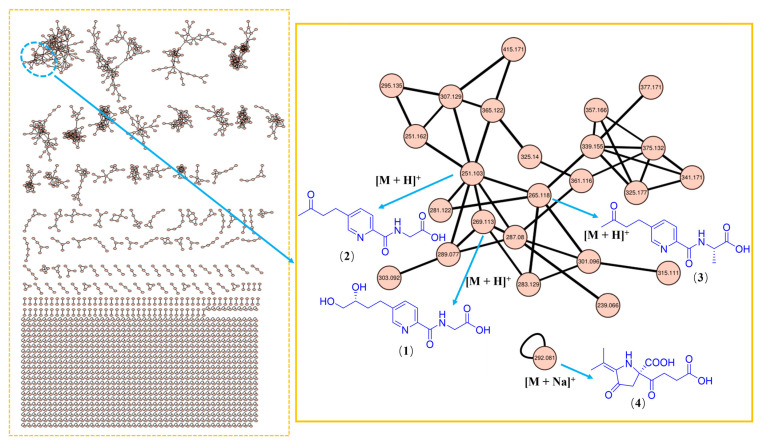
Molecular network of extract of the fungus *Samsoniella hepiali* W7. The cluster of new compounds (**1**–**4**) is expanded, and the nodes are marked with the *m*/*z* value of the parent ion.

**Figure 2:**
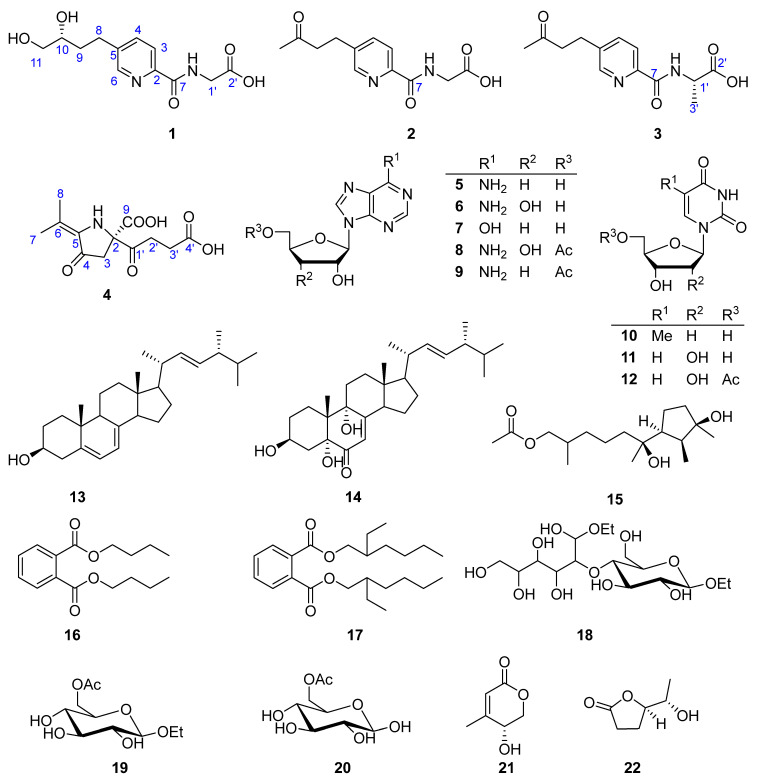
Compounds **1**–**22** from *Samsoniella hepiali* W7.

As part of our ongoing exploration of structurally novel and bioactive secondary metabolites from marine microbes [21,22,23,24], we performed a systematic investigation, aided by a GNPS molecular networking approach, on the crude extract of the culture of fungus *Samsoniella epialid* W7, isolated from the sulfide at a depth of 3073 m in the South Atlantic. Prior to isolation, the GNPS network of the extract revealed a big molecular cluster within which none of the nodes were identified by the internal compound library (Figure 1), indicating a certain possibility of new compounds to be found among these metabolites. A further scrutiny of their MS/MS fragmentation pattern (Figure S29), followed by HRMS-based molecular formula analysis, strongly suggested that they were new analogues of fusaric amides [20]. The systematic isolation, along with targeted purification, finally yielded three new fusaric acids, hepialiamides A–C (**1**–**3**), and one novel hybrid polyketide, hepialide (**4**), together with 18 known compounds (**5**–**22**) (Figure 2). The known compounds **8**, **11**, **13**, **21**, and **22** exhibited inhibitory activity against LPS-induced NO production. Herein, we report the fermentation, isolation, structure characterization and bioactivity of these isolates.

## 2. Results and Discussion

Compound **1** was isolated as a yellow oil. The HRESI(+)MS of **1** exhibited a protonated molecular ion [M + H]^+^ at *m*/*z* 269.1142, indicating a molecular formula of C_12_H_17_N_2_O_5_, accounting for seven degrees of unsaturation (DoU). The ^1^H NMR (Table 1) data of **1** presented the diagnostic signals of three aromatic protons, typically an ABX coupling system at *δ*_H_ 8.51 (1H, d, *J* = 1.5 Hz, H-6), 7.96 (1H, d, *J* = 8.0 Hz, H-3), and 7.83 (1H, dd, *J* = 8.0, 2.0 Hz, H-4), indicating the presence of a 2,5-disubstituted pyridine ring in **1**. This was confirmed by the key heteronuclear multiple bond correlation (HMBC) cross-peaks from H-3 to C-5 (*δ*_C_ 141.4), from H-4 to C-2 (*δ*_C_ 147.3) and C-6 (*δ*_C_ 148.5), and from H-6 to C-2 and C-4 (*δ*_C_ 137.4), as well as by the correlation spectroscopy (COSY) correlations of H-3/H-4.

**Figure 3:**
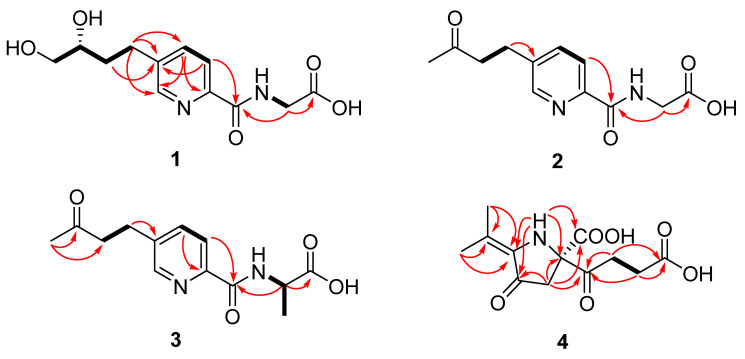
Key HMBC (arrows) and ^1^H–^1^H COSY (bold) correlations of compounds **1**–**4**.

**Figure 4:**
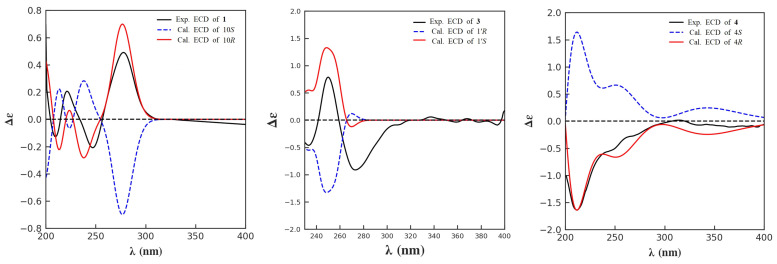
Experimental and calculated ECD spectra of **1**, **3**, and **4**.

Furthermore, four methylene groups at *δ*_H_ 3.97 (2H, s, H-1′), [2.71 (1H, m, H-8), 2.82 (1H, m, H-8)] and [1.56 (1H, m, H-9), 1.77 (1H, m, H-9)], [3.25 (1H, dd, *J* = 10.7, 5.7 Hz, H-11), 3.32 (1H, dd, *J* = 10.7, 5.6 Hz, H-11)] and one oxymethine at *δ*_H_ 3.39 (1H, m, H-10), were observed (Table 1). The ^1^H-^1^H COSY correlations of H-8/H-9/H-10/H-11, together with the HMBC correlations from H-11 to C-10 (*δ*_C_ 70.2) and C-9 (*δ*_C_ 34.7), from H-10 to C-9 and C-8 (*δ*_C_ 28.3), from H-9 to C-8 and C-5, and from H-8 to C-4, C-5, and C-6 indicated a 1,2-butanediol moiety at C-5. Further HMBC correlations of H-1′ to C-7 (*δ*_C_ 164.2) and C-2′ (*δ*_C_ 171.1) suggested the presence of a glycine moiety (Figure 3). An amido bond between C-2 of the pyridine ring and C-1′ of the glycine unit was confirmed by key HMBC correlations from H-3 and H-1′ to C-7. Thus, compound **1** was identified as a new fusaric acid derivative and given the trivial name hepialiamide A. The comparison of its optical rotation value (${\left[\alpha \right]}_{D}^{25}$ + 13) with those of similar known compounds, such as (*R*)-4-phenyl-1,2-butanediol (${\left[\alpha \right]}_{D}^{20}$ + 33) and (*S*)-4-phenyl-1,2-butanediol (${\left[\alpha \right]}_{D}^{20}$ − 34) [25], and comparative analysis of the experimental and theoretically calculated ECD curves established the absolute configuration of hepialiamide A to be 10*R* (Figure 4).

Compound **2** was isolated as a yellow oil. The HRESI(+)MS of **2** exhibited a protonated molecular ion [M + H]^+^ at *m*/*z* 251.0950. Analysis of the HRESI(+)MS and ^13^C NMR data revealed the molecular formula C_12_H_14_N_2_O_4_ of **2** indicating seven DoUs. The NMR data (Table 1) of **2** were closely related to those of **1**, except for the presence of a methyl ketone group at (*δ*_C_ 209.8, C-10) and (*δ*_C_ 29.9, *δ*_H_ 2.13, CH_3_-11) in **2**, which was supported by the HMBC correlations of H_3_-11 to C-10 and C-9 (*δ*_C_ 44.6). The left of the structure was confirmed by 2D NMR correlations, as shown (Figure 3). Therefore, **2** was identified as a new analogue of **1** and named hepialiamide B.

Compound **3** was obtained as a yellow oil with the molecular formula of C_13_H_16_N_2_O_4_ implied by its HRESI(+)MS spectrum. The 1D NMR data (Table 1) of **3** were similar to those of **2** except that **3** had an extra methyl group [*δ*_C_ 18.2 (C-4′)] at C-2′, which was confirmed by the COSY correlations of NH (*δ*_H_ 8.74, d)/H-1′ (*δ*_H_ 4.46, d)/H-3′ (*δ*_H_ 1.42, d) and the HMBC correlations from H-1′ to C-2′ (*δ*_C_ 173.8), C-3′ (*δ*_C_ 17.5), and C-7 (*δ*_C_ 163.5). The absolute configuration of the chiral center C-1′ in **3** was established to be *S*, as indicated by the agreement between the calculated ECD spectrum of **3** and the experimental data (Figure 4). Hence, compound **3** was identified as another new fusaric acid derivative, and named hepialiamide C.

Compound **4** was obtained as a yellow oil. The HRESI(+)MS of **4** exhibited a sodiated molecular ion [M + Na]^+^ at *m*/*z* 292.0800, suggesting a molecular formula (calculated for C_12_H_15_NO_6_Na, 292.0797) that requires six DoUs. The ^1^H NMR (Table 2) data of **4** presented the diagnostic signals of three methylene groups at *δ*_H_ 2.28 (2H, t, *J* = 6.3 Hz, H-2′), 2.64 (2H, q, *J* = 6.9 Hz, H-3′), and 3.10 (2H, q, *J* = 15.0 Hz, H-3), and two methyl groups at 2.08 (3H, s, H-8) and 1.81 (3H, s, H-9). In the ^13^C NMR spectrum, 12 carbon signals were observed and classified as two methyl (*δ*_C_ 20.8, 18.5), three methylene (*δ*_C_ 47.4, 36.3, 27.4), and seven non-protonated carbons (*δ*_C_ 207.2, 197.0, 173.5, 171.4, 1129.1, 122.3, 70.3), with the aid of HSQC data. In the ^1^H-^1^H COSY spectrum of **4**, homonuclear vicinal coupling correlation (Figure 3) between H-7 and H-8, along with the HMBC correlations from H-7 to C-5/6, from H-8 to C-5/6, indicated an isopropenyl group. The HMBC cross-peaks observed from H-3 to C-2/4/9, H-7/8 to C-4, and the diagnostic signals from an exchangeable proton to C-2/4/5/9 permitted the construction of a five-member ring. Finally, based on the rest of the HMBC correlations from H-3 to C-1′, H-2′ to C-1′/3′/4′, from H-3′ to C-1′/4′, and the COSY signals between H-2′ to H-3′, together with a comparable analysis of the chemical shifts of C-2/5 in **4** and those in literature [26,27], the planar structure o f **4** was assigned as shown.

To further assign the absolute configuration of C-2, a theoretical calculation of the ECD spectra of the 4*R*- and 4*S*-isomers was conducted. The experimental ECD spectrum of **4** was in good accordance with the calculated result for the 4*R*-configured **4** (Figure 4). The structure of compound **4** was thus determined and named hepialide.

By comparing the NMR, MS, ECD, and OR data with those reported in the references, 18 previously described components were identified as cordycepin (**5**) [28], adenosine (**6**) [29], 3′-deoxyinosine (**7**) [30], 5′-*O*-acetyladenosine (**8**) [31], 5′-acetyl-3′-deoxyadenosine (**9**) [32], thymidine (**10**) [33], uridine (**11**) [34], 5′-*O*-acetyluridine (**12**) [35], ergosterol (**13**) [36], 3*β*,5*α*,9*α*-trihydroxyergosta-7,22-diene-6-one (**14**) [37], 12-acetoxycycloneran-3,7-diol (**15**) [38], dibutyl phthalate (**16**) [39], di-(2-ethylhexyl)phthalate (**17**) [40], oryzasaccharide A (**18**) [41], d-glucopyranoside, ethyl, 6-acetate (**19**) [42], 6-*O*-acetyl-d-glucose (**20**) [43], walterolactone A (**21**) [44], and (4*R*,5*S*)-5-hydroxyhexan-4-olide (**22**) [45]. Among them, **16** and **17** were previously described as plasticizers and common contaminants in natural products [46].

Naturally occurring aminated fusaric acid derivatives are uncommon in fungal metabolites, as mentioned above. Hepialiamides A–C (**1**–**3**) feature an oxidized fusaric acid core structure with a glycine or alanine coupled through an amide bond, representing the third examples of fusaric amides of fungal origin. Hepialide is a novel PKS-NRPS hybrid polyketide with an unusual isopropenylated pyrrolidone moiety that resembles the tetramic acid.

Neuroinflammation, characterized by the activation of microglia cells, is a common denominator in diverse neurological conditions, including neurodegenerative diseases, traumatic brain injury, and neuroinfectious disorders. Microglia cell activation contributes significantly to the progression of these diseases by releasing pro-inflammatory mediators, exacerbating neuronal damage and impairing synaptic plasticity [47]. Hence, inhibiting glial cell activation emerges as a promising therapeutic strategy. The rich diversity and unique chemical structures of marine compounds provide a vast pool of potential drugs. Inflammatory tests were conducted on BV-2 microglia cells. Stimulation of the cells with bacterial products like lipopolysaccharide (LPS) triggers the production of NO and pro-inflammatory cytokines, key mediators in inflammation [48]. Given the significance of high NO levels in inflammation, targeting inducible nitric oxide synthase (iNOS), the enzyme responsible for NO synthesis, has been suggested as an anti-inflammatory therapeutic approach.

**Figure 5:**
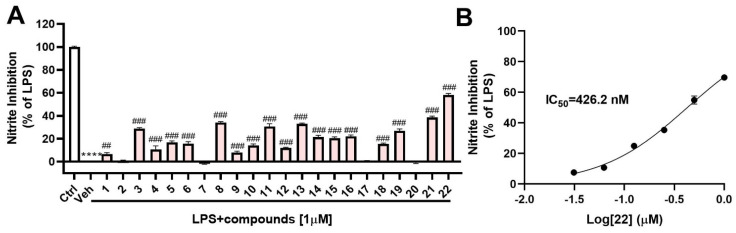
The impact of compounds on NO release in LPS-induced microglia cells: (**A**) Nitrite accumulation in the culture media was quantified using the Griess method, and nitrite production inhibition was calculated compared to the only LPS treated group. Results were normalized to the LPS condition and presented as mean ± SEM (*n* = 4); **** *p* < 0.0001 vs. Ctrl; ### *p* < 0.0001, ## *p* < 0.01 vs. Veh; (**B**) Inhibitory curve of compound **22** against nitrite production.

In this LPS-induced BV-2 microglia cell model, we evaluated the inhibitory activity of these compounds on NO production at a low concentration (1 μM). The cells were pre-exposed to the compounds for 1 h, followed by LPS stimulation for 24 h, and subsequently, the concentration of nitrite, the primary metabolite derived from NO, was quantified. The results indicated that compounds **8**, **11**, **13**, **21**, and **22** reduced NO production by 34.2 ± 1.6%, 30.7 ± 4.8%, 32.9 ± 1.6%, 38.6 ± 2.1%, and 58.2 ± 2.6%, respectively, with respect to vehicle-treated stimulated cells (Figure 5A). Particularly noteworthy, further analysis revealed that compound **22** exhibited a remarkable dose-dependent inhibitory effect, with an IC_50_ value of 426.2 nM (Figure 5B).

These compounds, especially compound **22**, frequently demonstrate anti-inflammatory and immunomodulatory properties, rendering them promising candidates for the development of specific and potent inhibitors that target microglia cell activation pathways. Further research is needed for in-depth evaluation.

## 3. Materials and Methods

### 3.1. General Experimental Procedures

Optical rotations were recorded on a Rudolph IV Autopol automatic polarimeter at 25 °C. NMR spectra, including ^1^H, ^13^C, DEPT, HSQC, COSY, HMBC, and NOESY were measured on a Bruker Avance 400 MHz spectrometer. Chemical shifts were recorded in δ values using solvent signals (DMSO-*d*_6_: *δ*_H_ 2.50/*δ*c 39.5; methanol-*d*_4_: *δ*_H_ 3.31/*δ*c 49.0; CDCl_3_: *δ*_H_ 7.27/*δ*c 77.0) as references. HRESIMS data were measured on a Xevo G2 Q-TOF mass spectrometer (Waters Corporation, Milford, MA, USA). Preparative and semipreparative HPLC were performed on an Agilent Technologies 1260 infinity instrument equipped with the DAD detector. UV spectra were recorded on a UV-8000 UV/Vis spectrometer (Shanghai Yuanxi Instrument Co., Ltd., Shanghai, China). Column chromatography (CC) was performed on ODS (50 μm, Daiso, Hiroshima, Japan), silica gel (Qingdao Marine Chemistry Co., Ltd., Qingdao, China), and Sephadex LH-20 (Amersham Pharmacia Biotech AB, Uppsala, Sweden). The TLC plates were visualized under UV light or by spraying with 10% H_2_SO_4_. Solvents for isolation were analytical grade. HRMS and MS/MS data were acquired with ultra-high performance liquid chromatography–quadrupole time-of-flight mass spectrometry (UHPLC-Q-TOF MS, AB SCIEX Triple TOF 5600^+^, Waters Corporation, Milford, MA, USA), with a Phenomenex Kinetex^®^ C18 (100 × 2.1 mm, 2.6 μm).

### 3.2. Fungal Identification, Fermentation, and Extract

The strain W7 was isolated from a sulfide sample (W 14.52°, S 13.59°) at a depth of 3073 m from the South Atlantic. It was identified as *Samsoniella hepiali* (GenBank accession number OR398925), as the ITS DNA gene sequence alignment demonstrated its similarity to *Samsoniella hepiali* CGMCC 3.17103 (GenBank accession number NR_160318.1). The strain is preserved at the Key Laboratory of Marine Biogenetic Resources, Third Institute of Oceanography, Ministry of Natural Resources (Xiamen, China). The strain *Samsoniella hepiali* was cultured on a PDA plate at 25 °C for 3 days. Fresh mycelia and spores were inoculated into 250 mL Erlenmeyer flasks (×10) containing 30 mL PDB medium and incubated in a 180 rpm rotary shaker at 28 °C for 5 days. Then, the spore cultures were used to inoculate 25 × 1 L Erlenmeyer flasks containing rice medium (80 g rice and 120 mL distilled H_2_O for each flask) to perform large-scale fermentation. After 25 days, the fermented culture was extracted with EtOAc three times to provide a crude extract. The extract was redissolved in MeOH and extracted with petroleum ether (PE) three times. The MeOH solution was evaporated under reduced pressure to obtain a defatted extract (25 g).

### 3.3. UHPLC-Q-TOF and Molecular Networking Analysis

The crude extract was analyzed by LC-MS/MS, equipped with an Atlantis^TM^ Premier BEH C_18_ AX (2.1 × 100 mm, 1.7 μm) column. Samples were dissolved in MeOH with 1 mg/mL. A 10 μL aliquot of each sample was injected and eluted, using a gradient program with water + 0.1% formic acid (A) and CH_3_CN + 0.1% formic acid (B). The elution started with a 1% B isocratic phase for 1 min, followed by a gradient from 1% to 99% B in 10 min, maintaining 99% B for 3 min. Then, a gradient from 99% to 1% B in 1 min was applied, and 1% B was maintained for 3 min at a flow rate of 0.4 mL/min, with the temperature maintained at 40 °C. The full mass spectrometry (MS) survey scan was performed in positive electrospray ionization (ESI) mode within the range of 50 to 1000 Da. The MS/MS data were converted to mzXML files using MSConvert software (version 3.01). The converted MS/MS file was submitted to the GNPS platform for molecular networking for dereplication (https://gnps.ucsd.edu, accessed on 1 January 2016). Parameters for molecular network generation were set as follows: both precursor mass and MS/MS fragment ion tolerance were set at 0.02 Da, minimum pairs cosine score 0.6, minimum matched fragment ions 6, minimum cluster size 2, network TopK 10. The spectral networks were imported into Cytoscape 3.9.1 and visualized using the force-directed layout.

### 3.4. Isolation and Purification

The MeOH extract (25 g) was subjected to CC over silica gel using sequential gradient elution with CH_2_Cl_2_-MeOH (100:1, 50:1, 30:1, 20:1, 10:1, 5:1, 2:1, and 0:1) to obtain five fractions (Fr.1–Fr.5), based on TLC properties. Fraction Fr.1 (5 g) was chromatographed over ODS using gradient elution of MeOH-H_2_O (30 → 80%) to get five subfractions (Fr.1-1–Fr.1-5). Fr.1-1 (625 mg) was subjected to CC on Sephadex LH-20 (MeOH), followed by repeated CC over silica gel, using gradient PE-EtOAc (3:1 → 1:1) to yield **17** (125 mg) and **2** (62 mg). Fr.1-2 (34 mg) was directly separated by Sephadex LH-20 (MeOH) to yield **15** (2 mg). Fr.1-3 (215 mg) was subsequently separated by CC over Sephadex LH-20 (MeOH), silica gel (CH_2_Cl_2_-MeOH, 100:1 → 50:1), and preparative TLC (PTLC) (CH_2_Cl_2_-MeOH, 20:1) to obtain **22** (14 mg) and **21** (2 mg). Fr.1-4 (137 mg) was subjected to CC over Sephadex LH-20 (MeOH) and silica gel (CH_2_Cl_2_-MeOH, 75:1) to yield **3** (40 mg). Fr.1-5 (226 mg) was purified by CC over Sephadex LH-20 (MeOH), silica gel (CH_2_Cl_2_-MeOH, 100:1), and preparative TLC (PTLC) (CH_2_Cl_2_-MeOH, 100:1) to afford compound **14** (7 mg).

Fraction Fr.2 (1.3 g) was subjected to CC over ODS with MeOH-H_2_O (2% → 30%), followed by purification using CC on Sephadex LH-20 (MeOH), crystallization (MeOH), and silica gel (EtOAc-MeOH, 20:1 → 5:1) to yield **20** (84 mg), **18** (29 mg), 5 (46 mg), **6** (5 mg), **10** (11 mg), **7** (7 mg), **1** (15 mg), and **11** (26 mg).

Fraction Fr.3 (1.5 g) was subjected to CC over ODS with MeOH-H_2_O (2% → 50%) and CC on Sephadex LH-20 (MeOH) to yield **19** (4 mg), **4** (11 mg), **12** (11 mg), and **8** (4 mg).

Fraction Fr.4 (9.8 g) was separated by CC over silica gel using sequential gradient elution with PE-EtOAc (100:1, 50:1, 30:1, 20:1, 10:1) and Sephadex LH-20 (CH_2_Cl_2_-MeOH, 1:1) to yield **13** (320 mg).

Fraction Fr.5 (5.3 g) was subjected to CC over ODS with MeOH-H_2_O (10% → 50%), followed by purification using CC on Sephadex LH-20 (MeOH), HPLC (20% → 48%, MeOH-H_2_O), crystallization (MeOH), and silica gel (EtOAc-MeOH, 20:1) to yield **9** (2 mg) and **16** (4 mg).

*Hepialiamide A* (**1**): Yellow oil; ${\left[\alpha \right]}_{D}^{25}$ + 13 (*c* 0.10, MeOH), ECD (MeOH) λmax (Δ*ε*) 278 (0.07), 245 (−0.09), 217 (0.07); UV (MeOH) λmax (log *ε*) 229 (2.72), 269 (2.31) nm; HRESIMS *m*/*z* 269.1142 [M + H]^+^ (calcd for C_12_H_17_N_2_O_5_, 269.1137).

*Hepialiamide B* (**2**): Yellow oil; UV (MeOH) λmax (log *ε*) 230 (2.76), 269 (2.38) nm; ^1^H and ^13^C NMR data, Table 1; HRESIMS *m*/*z* 251.0950 [M + H]^+^ (calcd for C_12_H_15_N_2_O_4_, 251.1032).

*Hepialiamide C* (**3**): Yellow oil; ${\left[\alpha \right]}_{D}^{25}$ + 3 (*c* 0.10, MeOH); ECD (MeOH) λmax (Δ*ε*) 275 (−0.15), 250 (0.13), 213 (−0.17); UV (MeOH) λmax (log *ε*) 200 (3.06), 269 (2.23) nm; HRESIMS *m*/*z* 265.111 [M + H]^+^ (calcd for C_13_H_17_N_2_O_4_, 265.1188).

*Hepialide* (**4**): Yellow oil; ${\left[\alpha \right]}_{D}^{25}$ + 5 (*c* 0.10, MeOH); ECD (MeOH) λmax (Δ*ε*) 211 (−0.28); UV (MeOH) λmax (log *ε*) 228 (2.53), 291 (1.91) nm; HRESIMS *m*/*z* 292.0800 [M + Na]^+^ (calcd for C_12_H_15_NO_6_Na, 292.0797).

### 3.5. Theoretical ECD Calculation

As reported previously, conformational analysis was first performed via random searching in Sybyl-X 2.0 using the MMFF94S force field with an energy cutoff of 7.0 kcal/mol and an RMSD threshold of 0.2 Å. All conformers were consecutively optimized at PM6 and HF/6-31G(d) levels. Dominant conformers were re-optimized at the B3LYP/6-31G(d) level in the gas phase. The theoretical ECD spectra were calculated using the GIAO method at the MPW1PW91/6-31G (d, p) level in MeOH using Gaussian 09. The ECD spectrum was simulated in SpecDis (version 1.71) by overlapping Gaussian functions for each transition.

### 3.6. BV-2 Cell Culture and Treatment

BV-2 microglia cells were cultured in DMEM medium containing 10% fetal bovine serum and antibiotics (100 units/mL of penicillin and 100 g/mL of streptomycin) and maintained in a humidified 5% CO_2_ incubator at 37 °C. For the experiment, cells were seeded overnight into 24-wells with 2 × 10^4^ cells per well. The next day, cells were incubated with fresh culture medium containing the indicated concentration of compounds for an hour, followed by lipopolysaccharides (LPS) treatment (1 μg/mL). Cells treated with the vehicle (DMSO, 0.1%) served as the control.

### 3.7. Nitrite Quantification

The concentration of nitrite in the culture medium was determined using the Griess Reagent Kit (Therofisher, Shanghai, China). Briefly, 75 μL of cell culture supernatants was reacted with an equal volume of the Griess Reagent Kit for 30 min at room temperature, and the absorbance of the diazonium compound was obtained at a wavelength of 560 nm. Nitrite production by vehicle stimulation was designated as 100% inhibition (Ctrl) compared to LPS stimulation (Veh) for the experiment.

## 4. Conclusions

In summary, three new aminated fusaric acid derivatives, hepialiamides A–C (**1**–**3**), and one novel hybrid polyketide, hepialide (**4**), together with 18 known miscellaneous compounds (**5**–**22**), were isolated from the cultures of the deep-sea-derived fungus *Samsoniella hepiali* W7 with the aid of GNPS molecular networking. Hepialiamides A–C feature a glycine/alanine unit embedded in the fusaric acid backbone, representing the third examples of naturally occurring fusaric amides, while hepialide features an uncommon isopropenylated pyrrolidone moiety. Biologically, compound **8**, **11**, **13**, **21,** and **22** showed more than 30% inhibition against NO production induced by LPS at 1 μM, with **22** exhibiting a particularly low nanomole IC_50_, suggesting potential for developing potent anti-inflammatory drugs.

## Figures and Tables

**Table 1:** ^1^H (400 Hz) and ^13^C NMR (100 Hz) spectroscopic data of compounds **1**–**3**.

No.	1 ^a^ (*δ*_C_, Type)	1 ^a^ (*δ*_H_, Mult *J* in Hz)	2 ^b^ (*δ*_C_, Type)	2 ^b^ (*δ*_H_, Mult *J* in Hz)	3 ^a^ (*δ*_C_, Type)	3 ^a^ (*δ*_H_, Mult *J* in Hz)
2	147.3, C		148.4, C		147.4, C	
3	121.7, CH	7.96, d (8.0)	122.8, CH	7.96, d (8.0)	121.6, CH	7.93, d (7.6)
4	137.4, CH	7.83, dd (2.0, 8.0)	138.3, CH	7.77, dd (2.0, 8.0)	137.4, CH	7.82, d (8.0)
5	141.4, C		141.9, C		141.3, C	
6	148.5, CH	8.51, d (1.5)	150.1, CH	8.47, d (1.6)	148.7, CH	8.51, s
7	164.2, C		167.0, C		163.5, C	
8	28.3, CH_2_	2.71, m; 2.82, m	27.5, CH_2_	2.90, d (5.2)	26.1, CH_2_	2.85, m
9	34.7, CH_2_	1.56, m; 1.77, m	44.6, CH_2_	2.88, d (5.2)	43.3, CH_2_	2.84, m
10	70.2, CH	3.39, m	209.8, C		207.3, C	
11	65.8, CH_2_	3.25, dd (5.7, 10.7)	29.9, CH_3_	2.13, s	29.7, CH_3_	2.09, s
		3.32, dd (5.6, 10.7)				
1′	41.0, CH_2_	3.97, s	41.9, CH_2_	4.15, s	47.6, CH	4.46, d (7.1)
2′	171.1, C		172.8, C		173.8, C	
3′					17.5, CH_3_	1.42, d (7.1)
NH		8.92, t (6.0)		9.04, t (5.4)		8.74, d (7.3)

^a^ Measured in DMSO-*d*_6_. ^b^ Measured in methanol-*d*_4_.

**Table 2:** ^1^H (400 Hz) and ^13^C (100 Hz) NMR spectroscopic data of compound **4** in DMSO-*d*_6_.

No.	*δ*_C_, Type	*δ*_H_, Mult (*J* in Hz)
2	70.3, C	
3	47.4, CH_2_	3.15, d (18.0); 3.06, d (18.0)
4	197.0, C	
5	122.3, C	
6	129.1, C	
7	18.5, CH_3_	2.08, s
8	20.8, CH_3_	1.81, s
9	171.4, C	
1′	207.2, C	
2′	27.4, CH_2_	2.28, t (6.3)
3′	36.3, CH_2_	2.68, m; 2.60, m
4′	173.5, C	
NH		10.27, s

## Data Availability

The data presented in this study are available in Supplementary Materials.

## Supplementary Materials

The following are available online at https://www.mdpi.com/article/10.3390/md21110596/s1. Figures S1–S52: One-dimensional and two-dimensional NMR, UV spectra, ECD calculation details of compounds **1**–**4**, proton NMR spectra of **5**–**22**.
