# Supplementary material for: Hepialiamides A–C: Aminated Fusaric Acid Derivatives and Related Metabolites with Anti-Inflammatory Activity from the Deep-Sea-Derived Fungus Samsoniella hepiali W7
Source: Mar Drugs. 2023 Nov 16;21(11):596. doi: 10.3390/md21110596 (PMC10672582; doi:10.3390/md21110596)
Supplement: Supplementary file 1 [file marinedrugs-21-00596-s001.zip › marinedrugs-2702010-supplementary.pdf]

Supporting Information

Hepialiamides A–C: Aminated Fusaric Acid Derivatives and Related Metabolites with Anti-Inflammatory Activity from the Deep-Sea-Derived Fungus *Samsoniella hepiali* W7

Zheng-Biao Zou [1,2,†], Tai-Zong Wu [2,†], Long-He Yang[3], Xi-Wen He [3], Wen-Ya Liu [1], Kai Zhang [2], Chun-Lan Xie [2], Ming-Min Xie [2], Yong Zhang [2], Xian-Wen Yang [2,*] and Jun-Song Wang [1,*]

1 Center for Molecular Metabolism, School of Environmental and Biological Engineering, Nanjing University of Science and Technology, 200 Xiaolingwei Street, Nanjing 210094, China; zhengbiaozou@njust.edu.cn (Z.-B.Z.); wenyaliu2015@163.com (W.-Y.L.)

2 Key Laboratory of Marine Genetic Resources, Third Institute of Oceanography, Ministry of Natural Re-sources, 184 Daxue Road, Xiamen 361005, China; wutaizong@tio.org.cn (T.-Z.W.); z18252730063@163.com (K. Z.); xiechunlanxx@163.com (C.-L.X.); xiemingmin@tio.org.cn (M.-M.X.); zhangyong@tio.org.cn (Y. Z.)

3 Technical Innovation Center for Utilization of Marine Biological Resources, Third Institute of Oceanogra-phy, Ministry of Natural Resources, 184 Daxue Road, Xiamen 361005, China; longheyang@tio.org.cn (L.-H.Y.);

* Correspondence: wang.junsong@gmail.com (J.W.); yangxianwen@tio.org.cn (X.-W.Y.); Tel.: +86-25- 8431-5512 (J.W.); Tel.: +86-592-2195319 (X.-W.Y.)

† These authors contributed equally to this work.

Content

Figure S1. ^{1}H NMR spectrum of compound **1** in DMSO.

Figure S2. ^{13}C NMR spectrum of compound **1** in DMSO.

Figure S3. HSQC spectrum of compound **1** in DMSO.

Figure S4. ^{1}H– ^{1}H COSY spectrum of compound **1** in DMSO.

Figure S5. HMBC spectrum of compound **1** in DMSO.

Figure S6. NOESY spectrum of compound **1** in DMSO.

Figure S7. HRESIMS spectrum of compound **1**.

Figure S8. ^{1}H NMR spectrum of compound **2** in CD_3OD.

Figure S9. ^{13}C NMR spectrum of compound **2** in CD_3OD.

Figure S10. HSQC spectrum of compound **2** in CD_3OD.

Figure S11. ^{1}H– ^{1}H COSY spectrum of compound **2** in CD_3OD.

Figure S12. HMBC spectrum of compound **2** in CD_3OD.

Figure S13. NOESY spectrum of compound **2** in CD_3OD.

Figure S14. HRESIMS spectrum of compound **2**.

Figure S15. ^{1}H NMR spectrum of compound **3** in DMSO.

Figure S16. ^{13}C NMR spectrum of compound **3** in DMSO.

Figure S17. HSQC spectrum of compound **3** in DMSO.

Figure S18. ^{1}H– ^{1}H COSY spectrum of compound **3** in DMSO.

Figure S19. HMBC spectrum of compound **3** in DMSO.

Figure S20. NOESY spectrum of compound **3** in DMSO.

Figure S21. HRESIMS spectrum of compound **3**.

Figure S22. ^{1}H NMR spectrum of compound **4** in DMSO.

Figure S23. ^{13}C NMR spectrum of compound **4** in DMSO.

Figure S24. HSQC spectrum of compound **4** in DMSO.

Figure S25. ^{1}H– ^{1}H COSY spectrum of compound **4** in DMSO.

Figure S26. HMBC spectrum of compound **4** in DMSO.

Figure S27. NOESY spectrum of compound **4** in DMSO.

Figure S28. HRESIMS spectrum of compound **4**.

Figure S29. The proposed fragmentation scheme of compound **1** by ESI-QTrap-MS/MS.

Figure S30. ^{1}H NMR spectrum of compound **5** in DMSO.

Figure S31. ^{1}H NMR spectrum of compound **6** in DMSO.

Figure S32. ^{1}H NMR spectrum of compound **7** in CD_3OD.

Figure S33. ^{1}H NMR spectrum of compound **8** in DMSO.

Figure S34. ^{1}H NMR spectrum of compound **9** in CD_3OD.

Figure S35. ^{1}H NMR spectrum of compound **10** in CD_3OD.

Figure S36. ^{1}H NMR spectrum of compound **11** in CD_3OD.

Figure S37. ^{1}H NMR spectrum of compound **12** in CD_3OD.

Figure S38. ^{1}H NMR spectrum of compound **13** in $CDCl_3$.

Figure S39. ^{1}H NMR spectrum of compound **14** in CD_3OD.

Figure S40. ^{1}H NMR spectrum of compound **15** in CD_3OD.

Figure S41. ^{1}H NMR spectrum of compound **16** in CD_3OD.

Figure S42. ^{1}H NMR spectrum of compound **17** in CD_3OD.

Figure S43. ^{1}H NMR spectrum of compound **18** in CD_3OD.

Figure S44. ^{1}H NMR spectrum of compound **19** in CD_3OD.

Figure S45. ^{1}H NMR spectrum of compound **20** in CD_3OD.

Figure S46. ^{1}H NMR spectrum of compound **21** in CD_3OD.

Figure S47. ^{1}H NMR spectrum of compound **22** in CD_3OD.

Figure S48. Gibbs free energy and equilibrium populations of low-energy conformers of **1** in ECD calculations.

Figure S49. Gibbs free energy and equilibrium populations of low-energy conformers of **3** in ECD calculations.

Figure S50. Gibbs free energy and equilibrium populations of low-energy conformers of **4** in ECD calculations.

Figure S51. UV spectrum of compounds **1-4**.

Figure S52. Inhibitory effects of compounds **1**–**22** (1 μM) on LPS-induced nitrite production in BV-2.

Figure S1. ^{1}H NMR spectrum of compound **1** in DMSO.

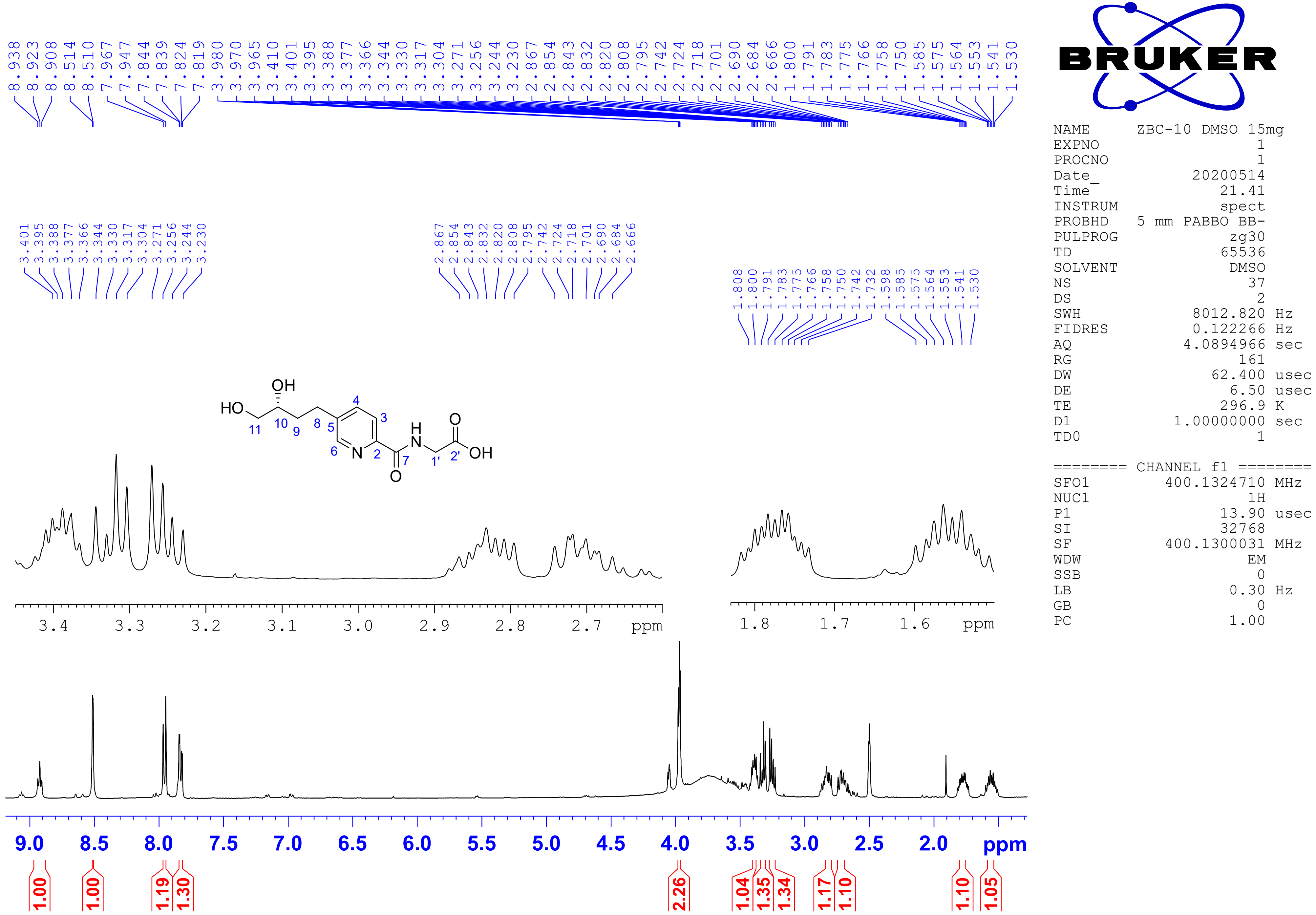

Figure S2. ^{13}C NMR spectrum of compound **1** in DMSO.

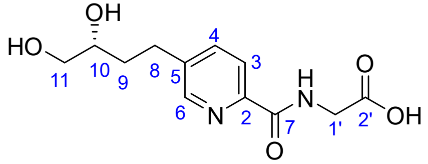

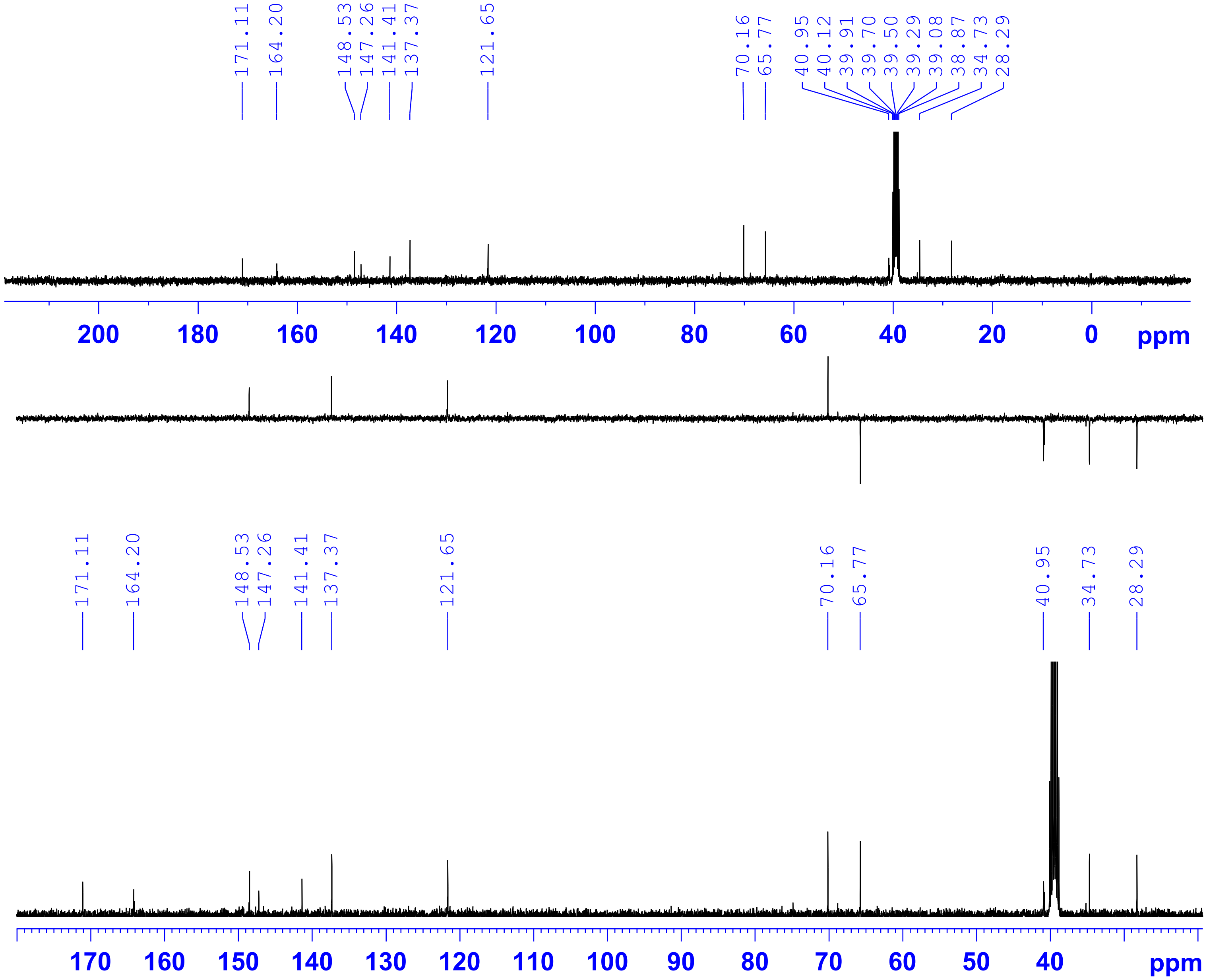

NAME	ZBC-10 DMSO 15mg
EXPNO	2
PROCNO	1
Date_	20200514
Time	21.45
INSTRUM	spect
PROBHD	5 mm PABBO BB-
PULPROG	zgpg30
TD	65536
SOLVENT	DMSO
NS	175
DS	4
SWH	24038.461 Hz
FIDRES	0.366798 Hz
AQ	1.3631988 sec
RG	203
DW	20.800 usec
DE	6.50 usec
TE	297.4 K
D1	2.00000000 sec
D11	0.03000000 sec
TD0	1

======== CHANNEL f1 ========

SFO1	100.6228293 MHz
NUC1	13C
P1	12.37 usec
SI	32768
SF	100.6128129 MHz
WDW	EM
SSB	0
LB	1.00 Hz
GB	0
PC	1.40

Figure S3. HSQC spectrum of compound **1** in DMSO.

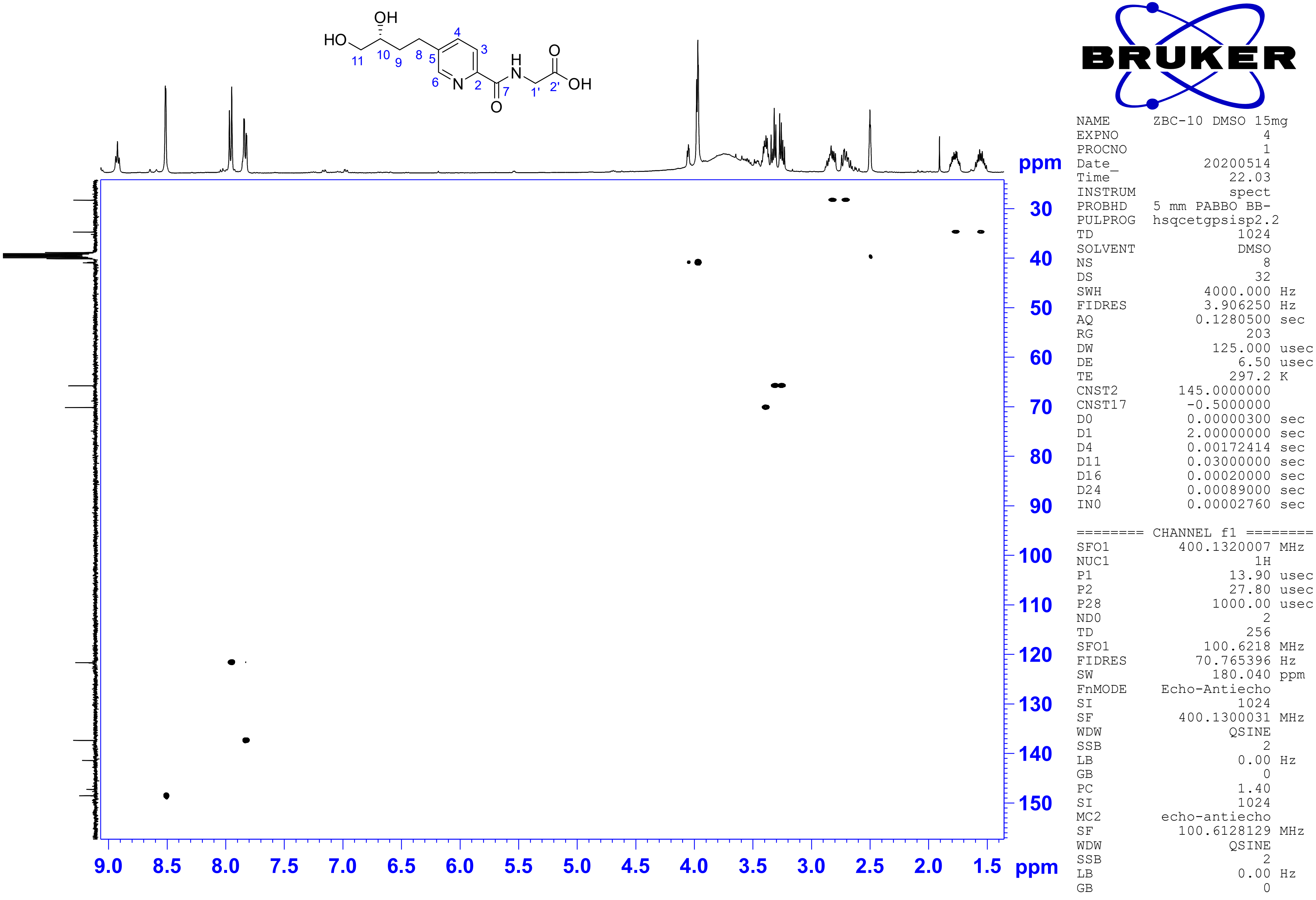

Figure S4. ^{1}H– ^{1}H COSY spectrum of compound **1** in DMSO.

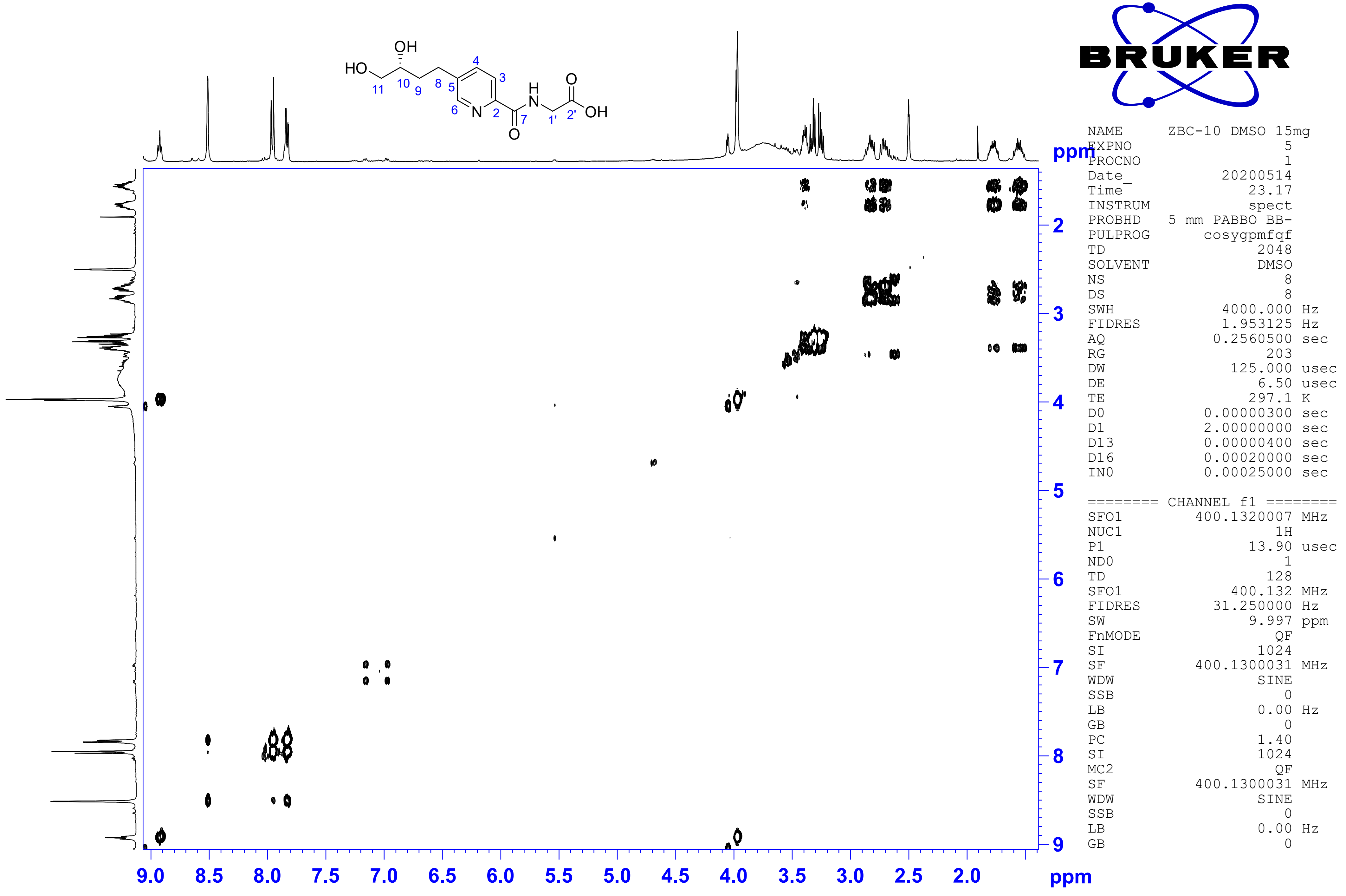

Figure S5. HMBC spectrum of compound **1** in DMSO.

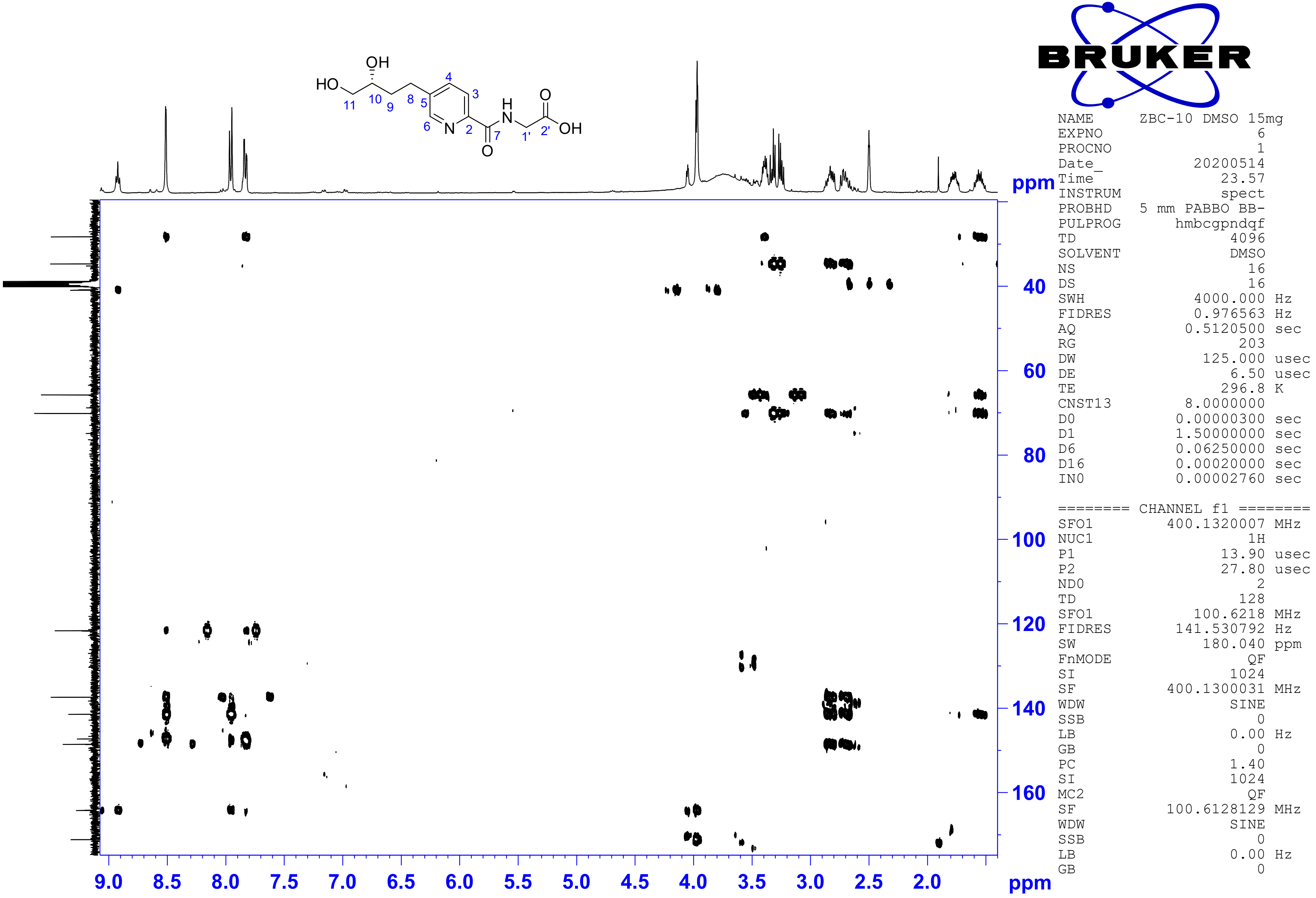

Figure S6. NOESY spectrum of compound **1** in DMSO.

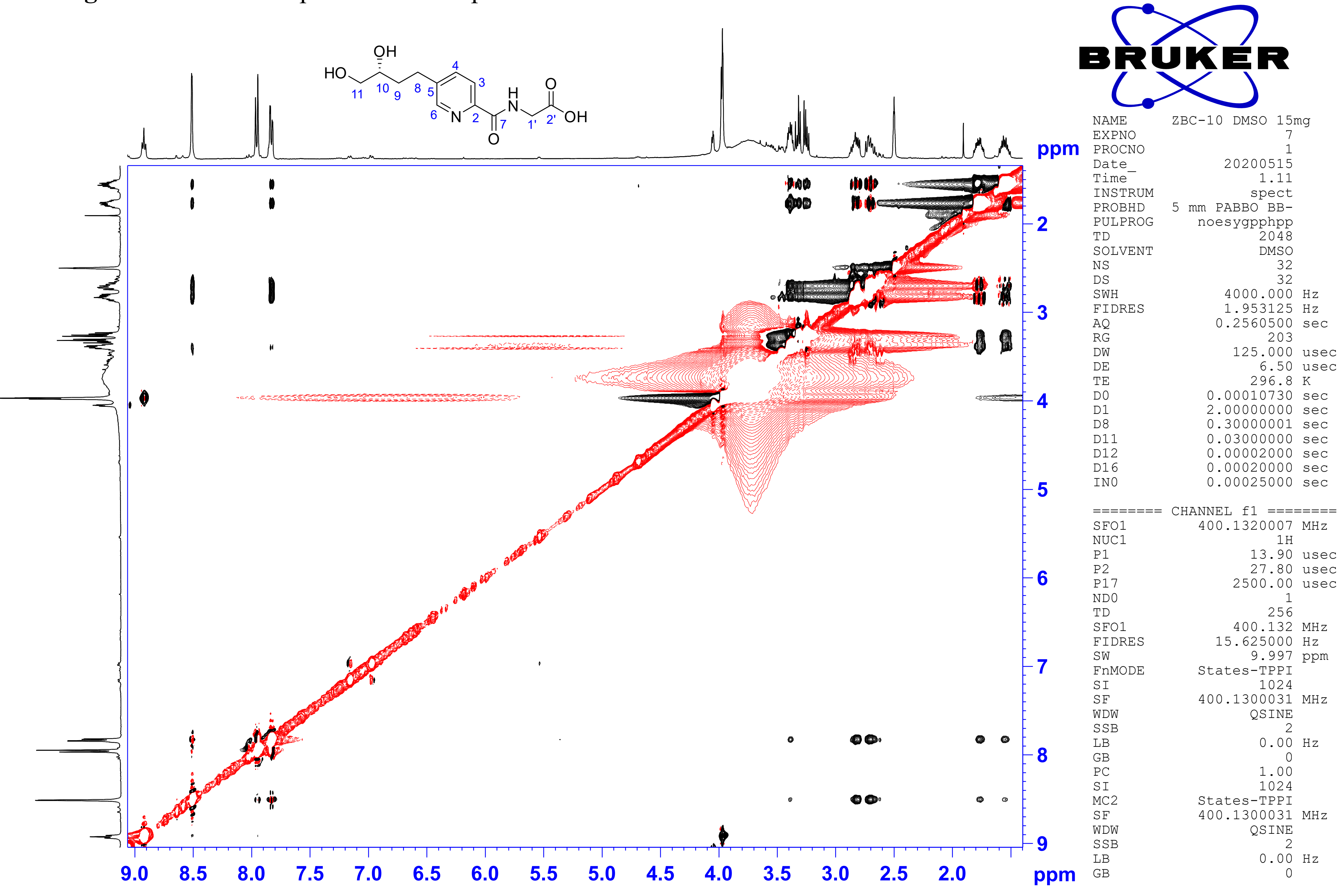

Figure S7. HRESIMS spectrum of compound **1**.

Elemental Composition Report

Single Mass Analysis

Tolerance = 40.0 mDa / DBE: min = -1.5, max = 50.0
Element prediction: Off
Number of isotope peaks used for i-FIT = 3

Monoisotopic Mass, Even Electron Ions
61 formula(e) evaluated with 4 results within limits (up to 50 best isotopic matches for each mass)
Elements Used:
C: 0-13 H: 0-50 N: 0-5 O: 0-5

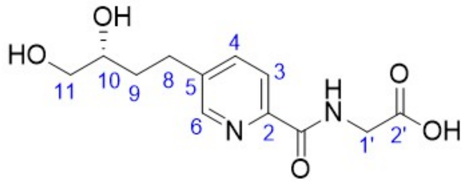

Chemical Formula: $C_{12}H_{17}N_2O_5^+$
Exact Mass: 269.1132

ZBC-10 101 (0.400) Cm (70:203)
1: TOF MS ES+

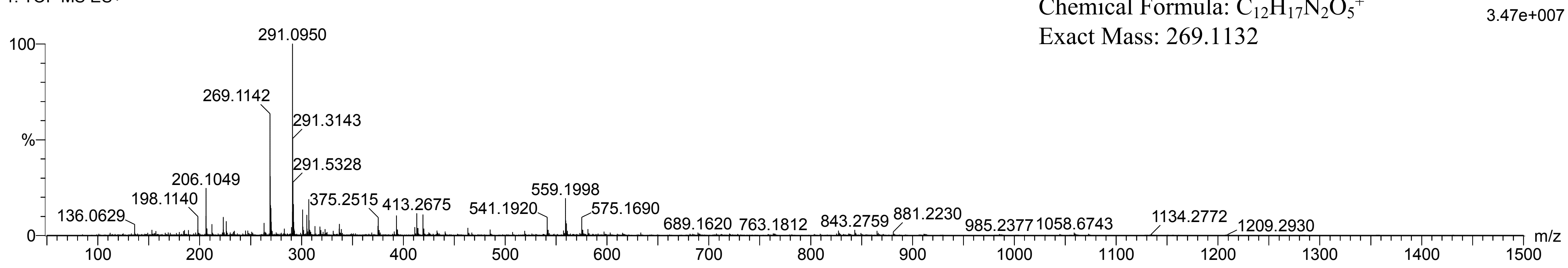

Minimum:				-1.5				
Maximum:		40.0	10.0	50.0				
Mass	Calc. Mass	mDa	PPM	DBE	i-FIT	Norm	Conf(%)	Formula
269.1142	269.1137	0.5	1.9	5.5	2594.2	0.563	56.97	C12 H17 N2 O5
	269.1501	-35.9	-133.4	4.5	2595.0	1.349	25.96	C13 H21 N2 O4
	269.1250	-10.8	-40.1	5.5	2595.9	2.253	10.51	C11 H17 N4 O4
	269.0886	25.6	95.1	6.5	2596.4	2.724	6.56	C10 H13 N4 O5

Figure S8. ^1H NMR spectrum of compound **2** in CD_3OD.

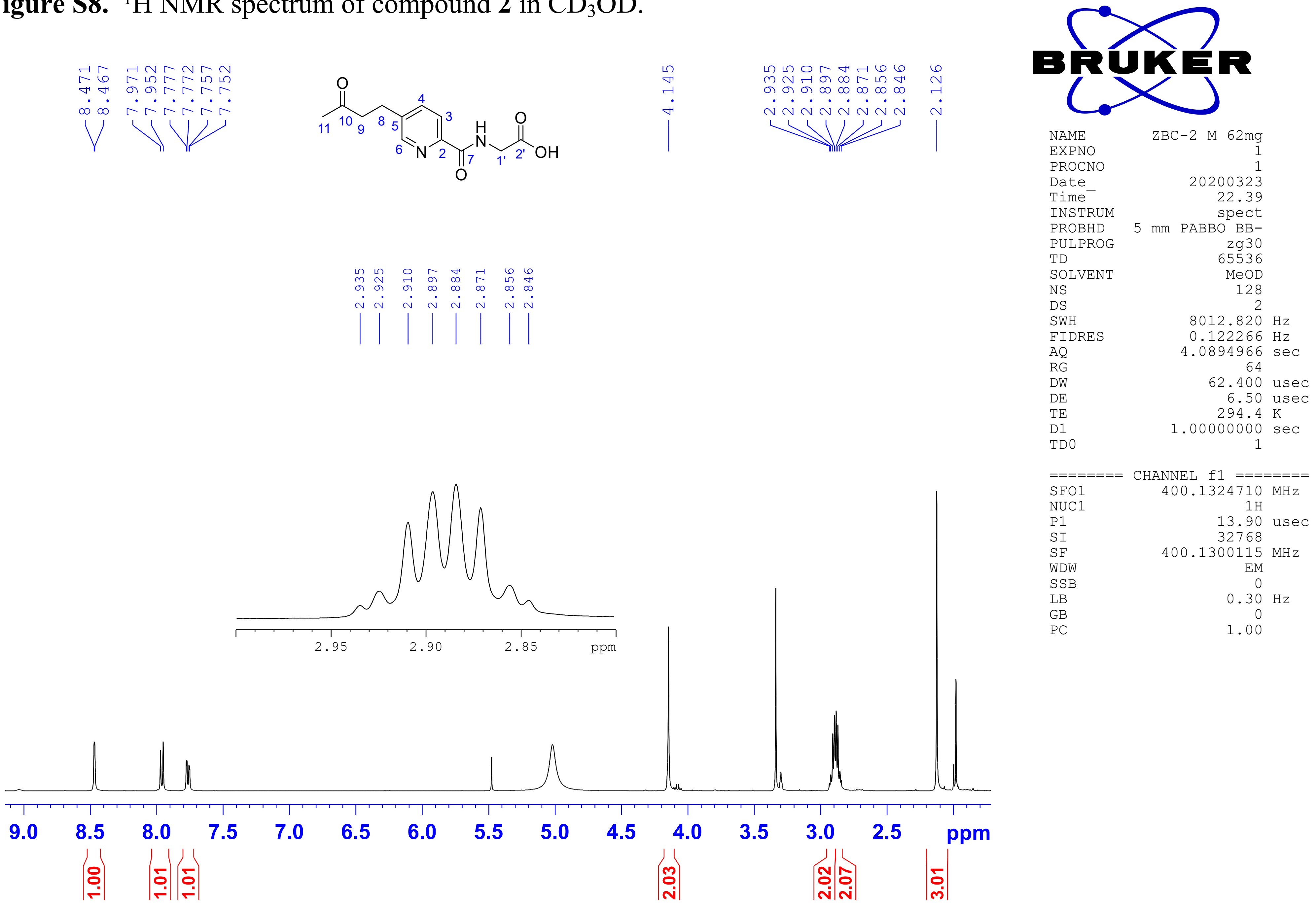

Figure S9. ^{13}C NMR spectrum of compound **2** in CD_3OD.

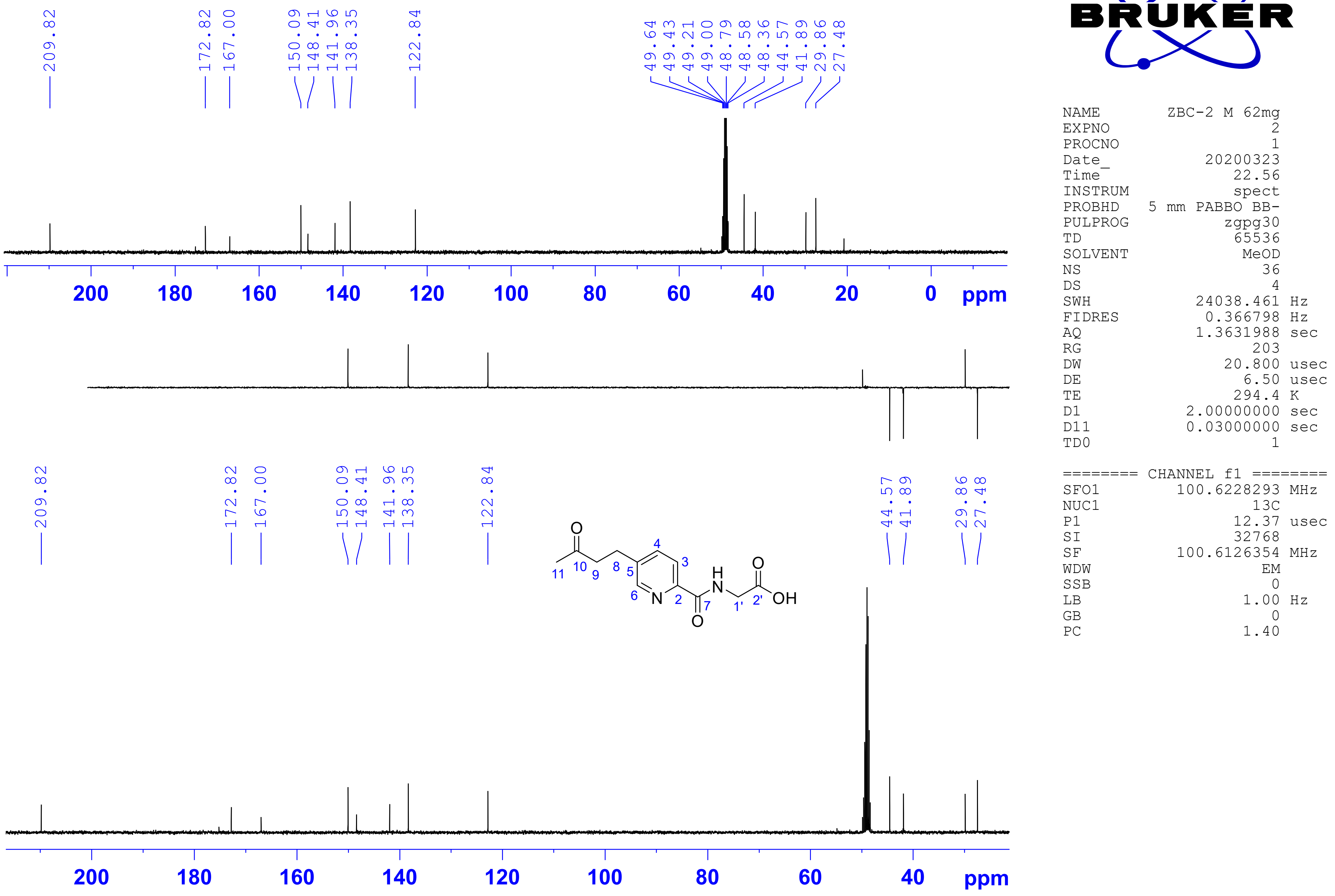

Figure S10. HSQC spectrum of compound **2** in CD_3OD.

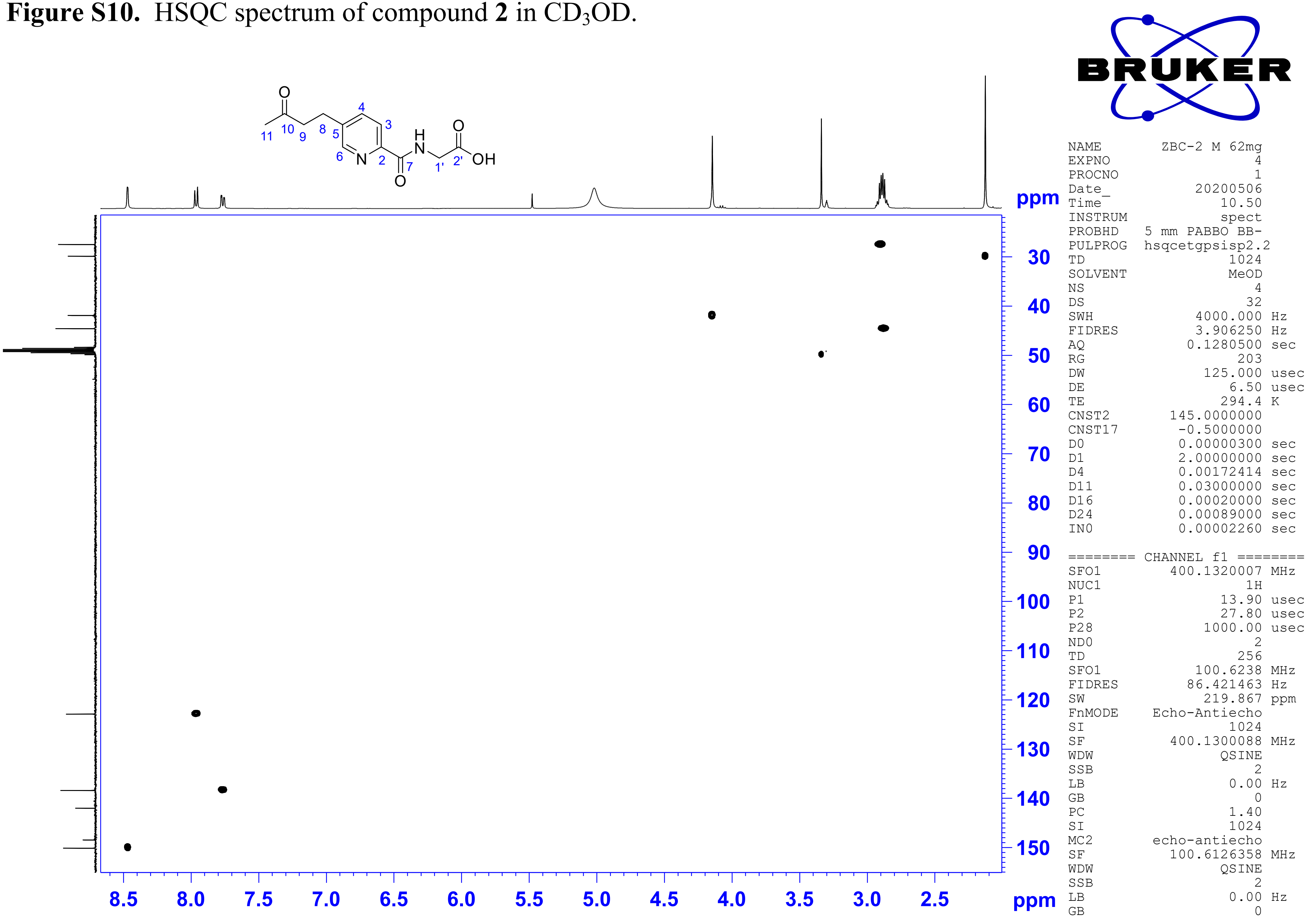

Figure S11. ^{1}H– ^{1}H COSY spectrum of compound **2** in CD_3OD.

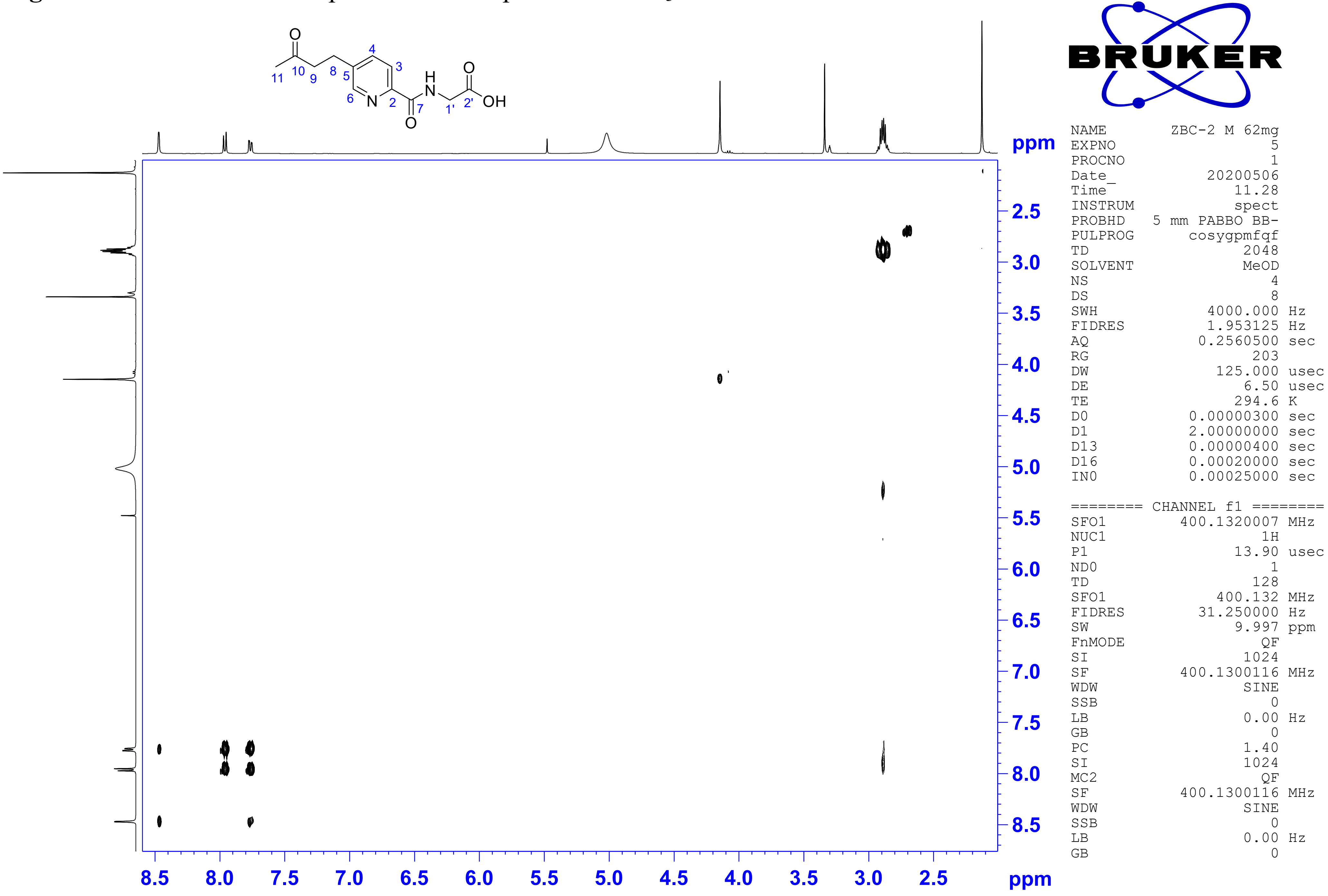

Figure S12. HMBC spectrum of compound **2** in CD_3OD.

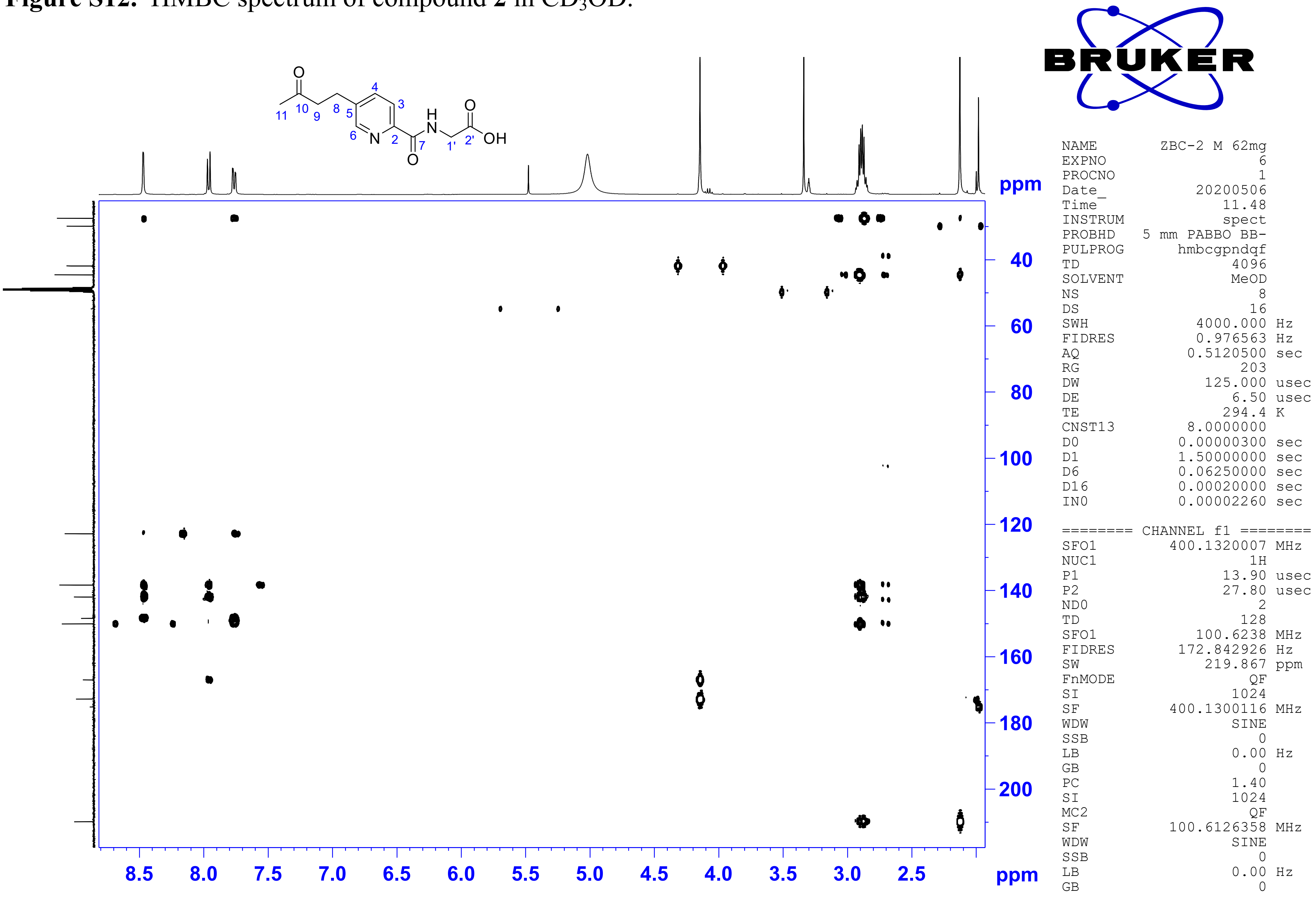

Figure S13. NOESY spectrum of compound **2** in CD_3OD.

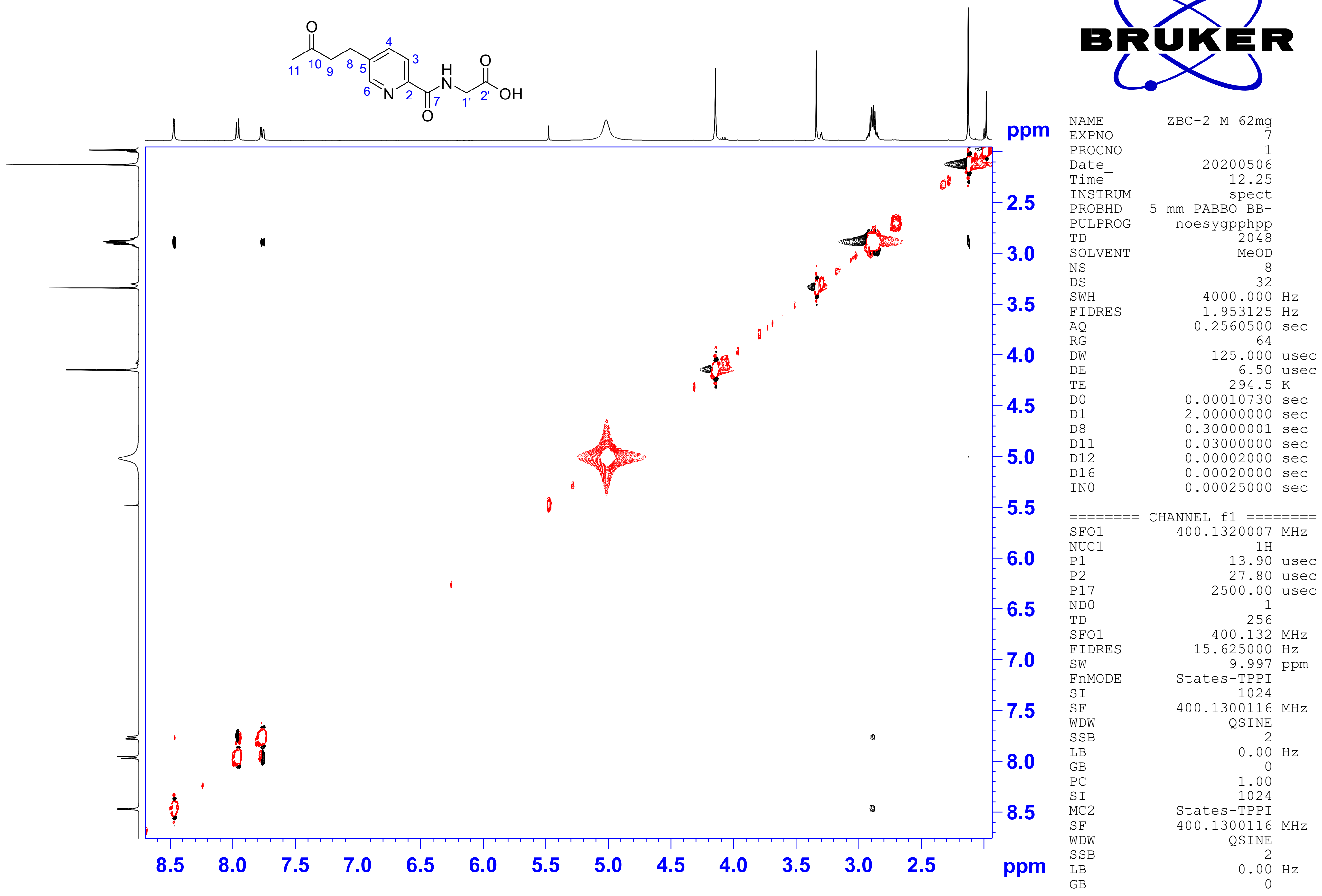

Figure S14. HRESIMS spectrum of compound **2**.

Elemental Composition Report

Single Mass Analysis

Tolerance = 40.0 mDa / DBE: min = -1.5, max = 50.0
Element prediction: Off
Number of isotope peaks used for i-FIT = 3

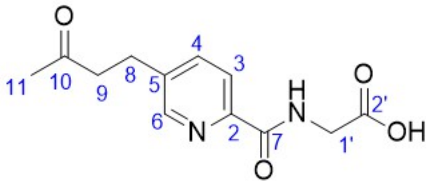

Chemical Formula: $C_{12}H_{15}N_2O_4^+$
Exact Mass: 251.1026

Monoisotopic Mass, Even Electron Ions
69 formula(e) evaluated with 6 results within limits (up to 50 best isotopic matches for each mass)
Elements Used:
C: 0-13 H: 0-50 N: 0-5 O: 0-5
ZBC-2-June-2 88 (0.354) Cm (88:183)
1: TOF MS ES+

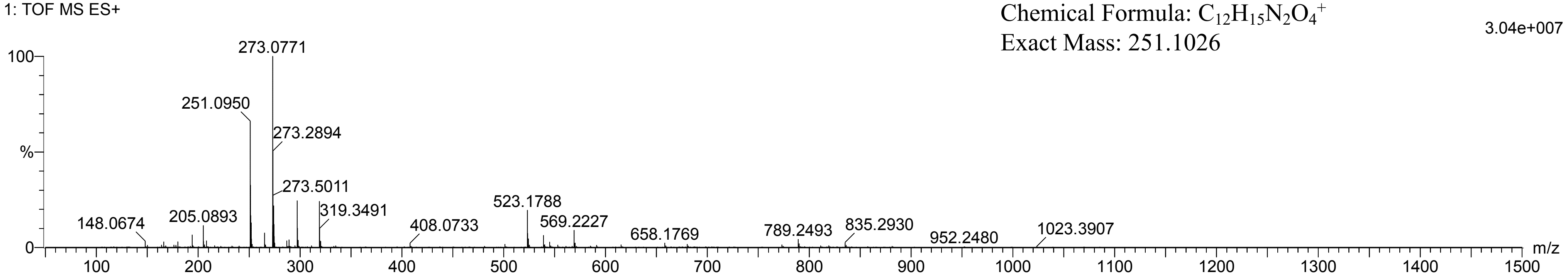

Minimum:				-1.5				
Maximum:		40.0	10.0	50.0				

Mass	Calc. Mass	mDa	PPM	DBE	i-FIT	Norm	Conf(%)	Formula
251.0950	251.0919	3.1	12.3	6.5	2224.2	0.761	46.71	C13 H15 O5
	251.1032	-8.2	-32.7	6.5	2224.8	1.419	24.19	C12 H15 N2 O4
	251.0569	38.1	151.7	12.5	2225.0	1.557	21.08	C13 H7 N4 O2
	251.0668	28.2	112.3	7.5	2226.8	3.395	3.36	C11 H11 N2 O5
	251.1144	-19.4	-77.3	6.5	2227.0	3.651	2.60	C11 H15 N4 O3
	251.0780	17.0	67.7	7.5	2227.3	3.881	2.06	C10 H11 N4 O4

Figure S15. ^{1}H NMR spectrum of compound **3** in DMSO.

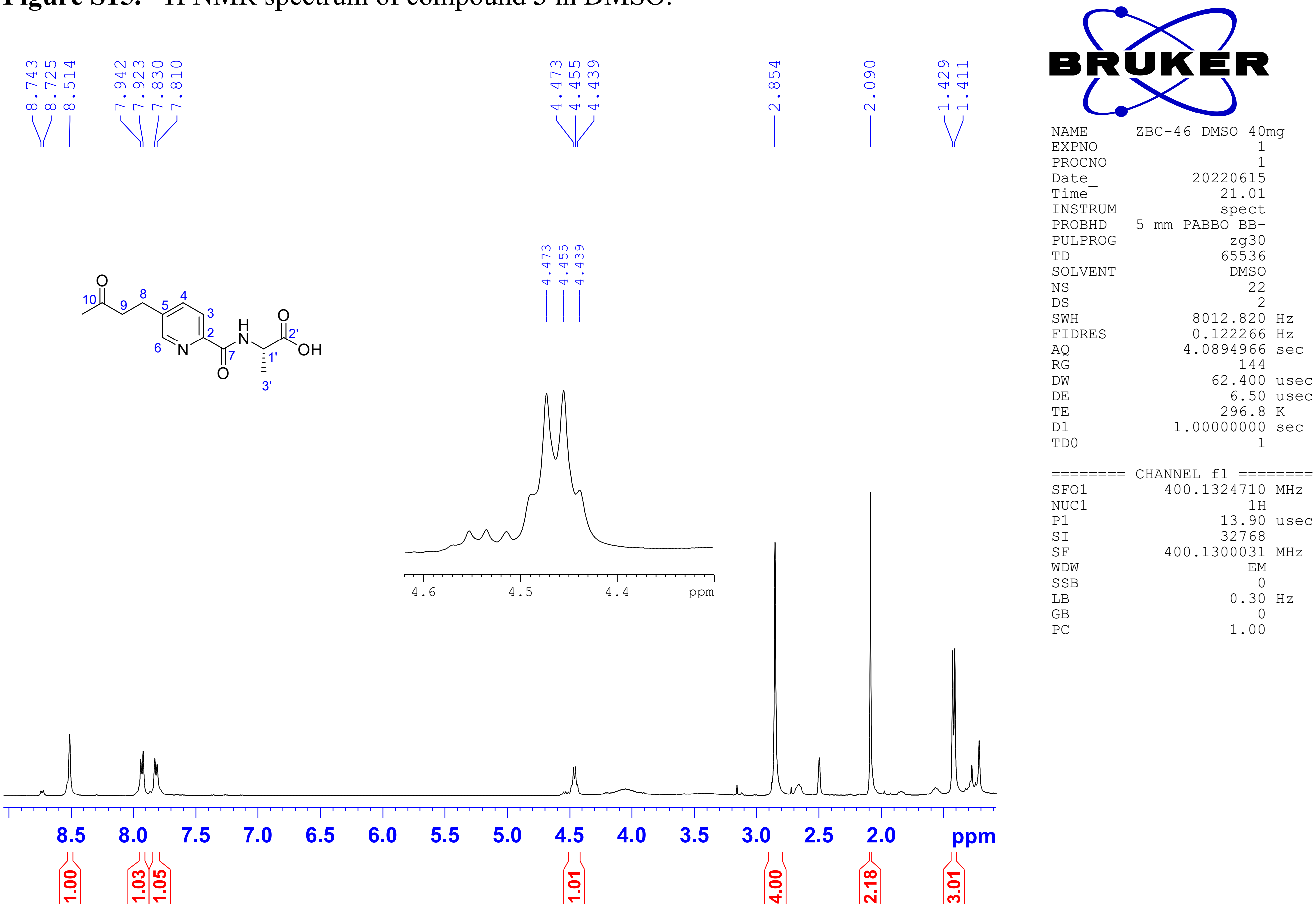

Figure S16. ^{13}C NMR spectrum of compound **3** in DMSO.

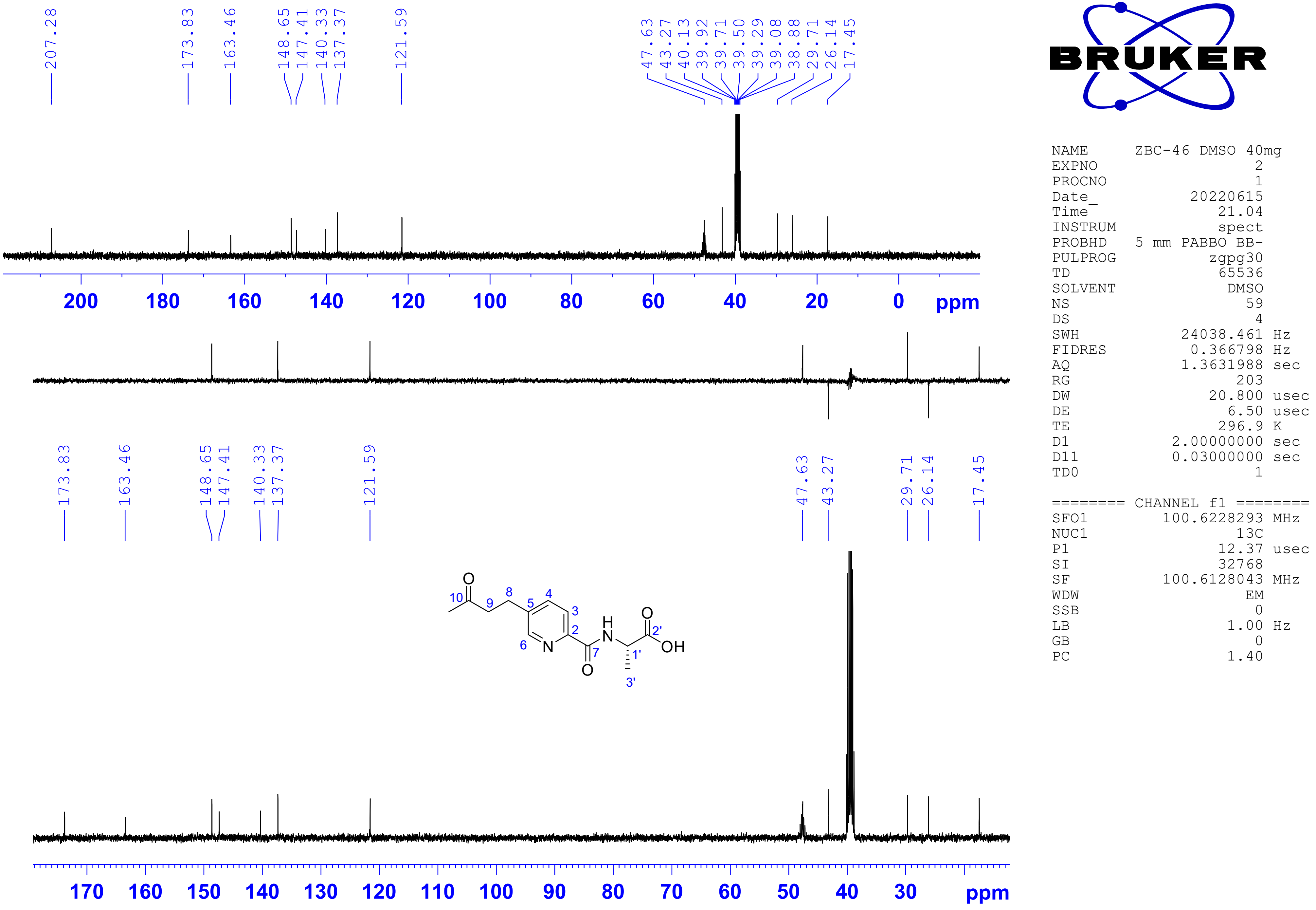

Figure S17. HSQC spectrum of compound **3** in DMSO.

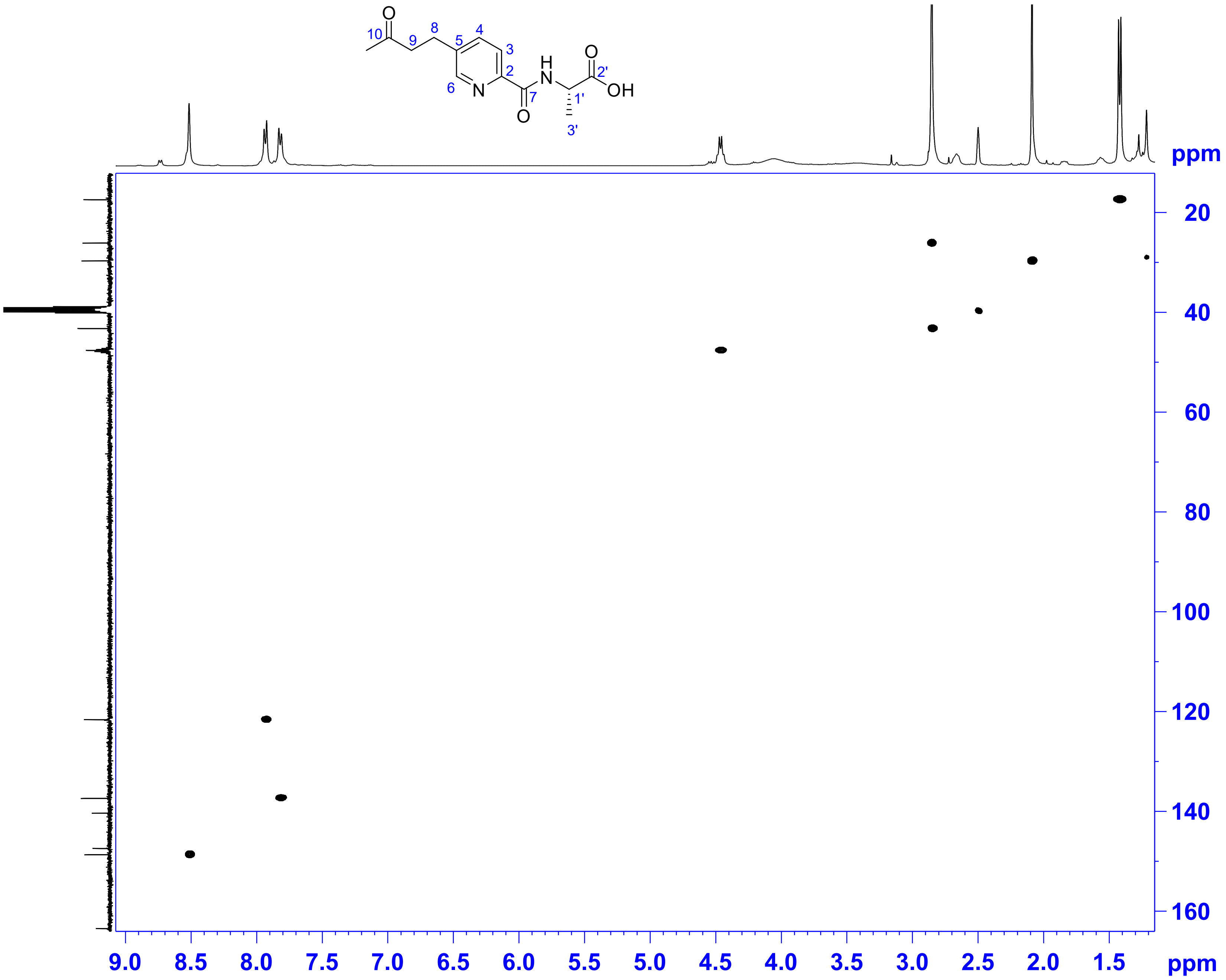

NAME ZBC-46 DMSO 40mg
EXPNO 4
PROCNO 1
Date_ 20220615
Time 21.17
INSTRUM spect
PROBHD 5 mm PABBO BB-
PULPROG hsqcetgpsisp2.2
TD 1024
SOLVENT DMSO
NS 16
DS 32
SWH 6393.862 Hz
FIDRES 6.244006 Hz
AQ 0.0801268 sec
RG 203
DW 78.200 usec
DE 6.50 usec
TE 296.9 K
CNST2 145.0000000
CNST17 -0.5000000
D0 0.00000300 sec
D1 2.00000000 sec
D4 0.00172414 sec
D11 0.03000000 sec
D16 0.00020000 sec
D24 0.00089000 sec
IN0 0.00002260 sec

======== CHANNEL f1 ========
SFO1 400.1332010 MHz
NUC1 1H
P1 13.90 usec
P2 27.80 usec
P28 1000.00 usec
ND0 2
TD 256
SFO1 100.6238 MHz
FIDRES 86.421463 Hz
SW 219.867 ppm
FnMODE Echo-Antiecho
SI 1024
SF 400.1300031 MHz
WDW QSINE
SSB 2
LB 0.00 Hz
GB 0
PC 1.40
SI 1024
MC2 echo-antiecho
SF 100.6128043 MHz
WDW QSINE
SSB 2
LB 0.00 Hz
GB 0

Figure S18. ^{1}H– ^{1}H COSY spectrum of compound **3** in DMSO.

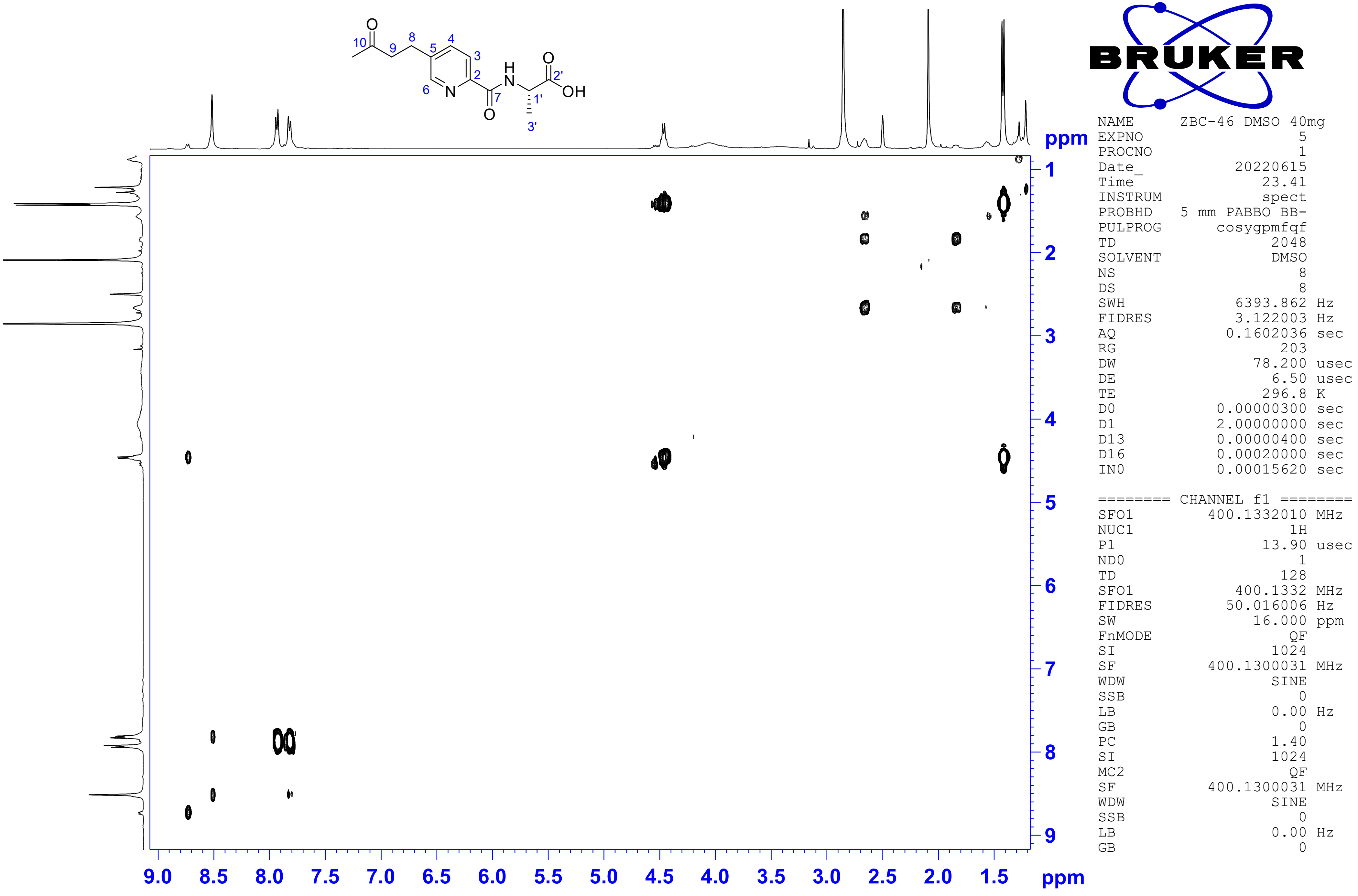

Figure S19. HMBC spectrum of compound **3** in DMSO.

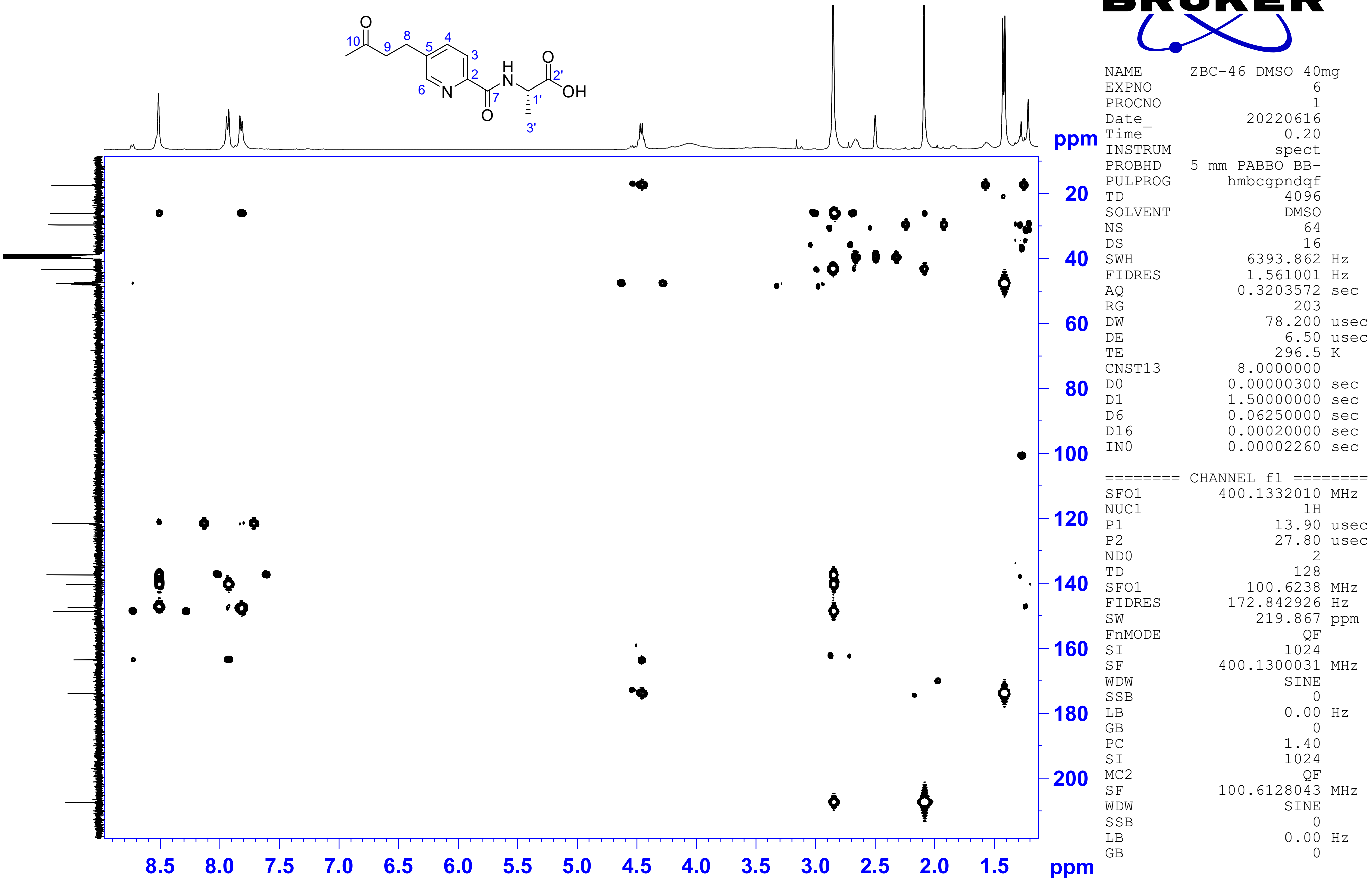

Figure S20. NOESY spectrum of compound **3** in DMSO.

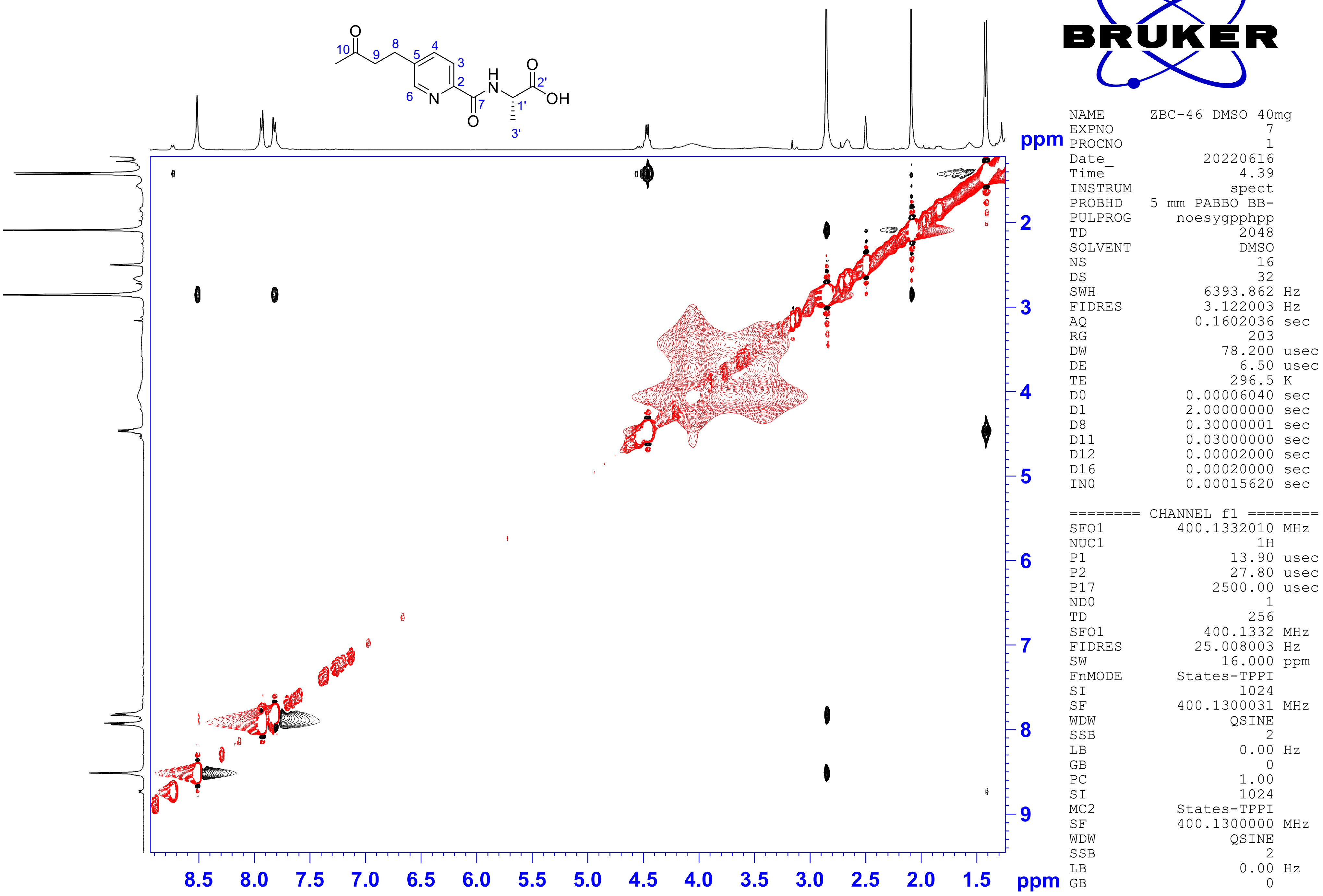

Figure S21. HRESIMS spectrum of compound **3**.

Elemental Composition Report

Single Mass Analysis

Tolerance = 40.0 mDa / DBE: min = -1.5, max = 50.0
Element prediction: Off
Number of isotope peaks used for i-FIT = 3

Monoisotopic Mass, Even Electron Ions
60 formula(e) evaluated with 4 results within limits (up to 50 best isotopic matches for each mass)
Elements Used:
C: 0-13 H: 0-50 N: 0-5 O: 0-5
ZBC-46-June 120 (0.468) Cm (32:193)
1: TOF MS ES+

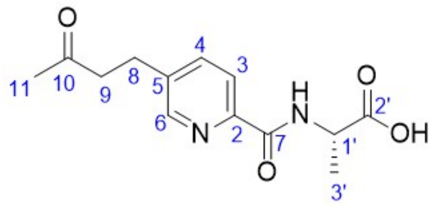

Chemical Formula: $C_{13}H_{17}N_2O_4^{+}$
Exact Mass: 265.1183

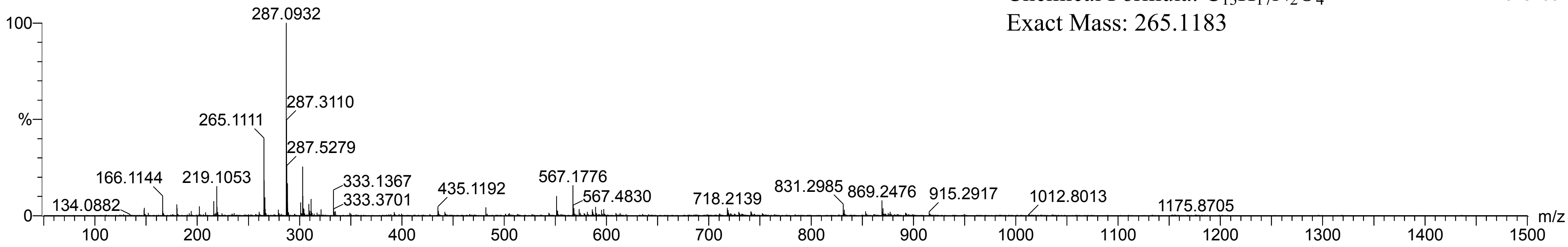

Minimum:				-1.5				
Maximum:		40.0	10.0	50.0				

Mass	Calc. Mass	mDa	PPM	DBE	i-FIT	Norm	Conf(%)	Formula
265.1111	265.1188	-7.7	-29.0	6.5	2329.5	0.498	60.74	C13 H17 N2 O4
	265.0824	28.7	108.3	7.5	2330.6	1.596	20.28	C12 H13 N2 O5
	265.1301	-19.0	-71.7	6.5	2331.3	2.234	10.71	C12 H17 N4 O3
	265.0937	17.4	65.6	7.5	2331.5	2.493	8.27	C11 H13 N4 O4

Figure S22. 1H NMR spectrum of compound 4 in DMSO.

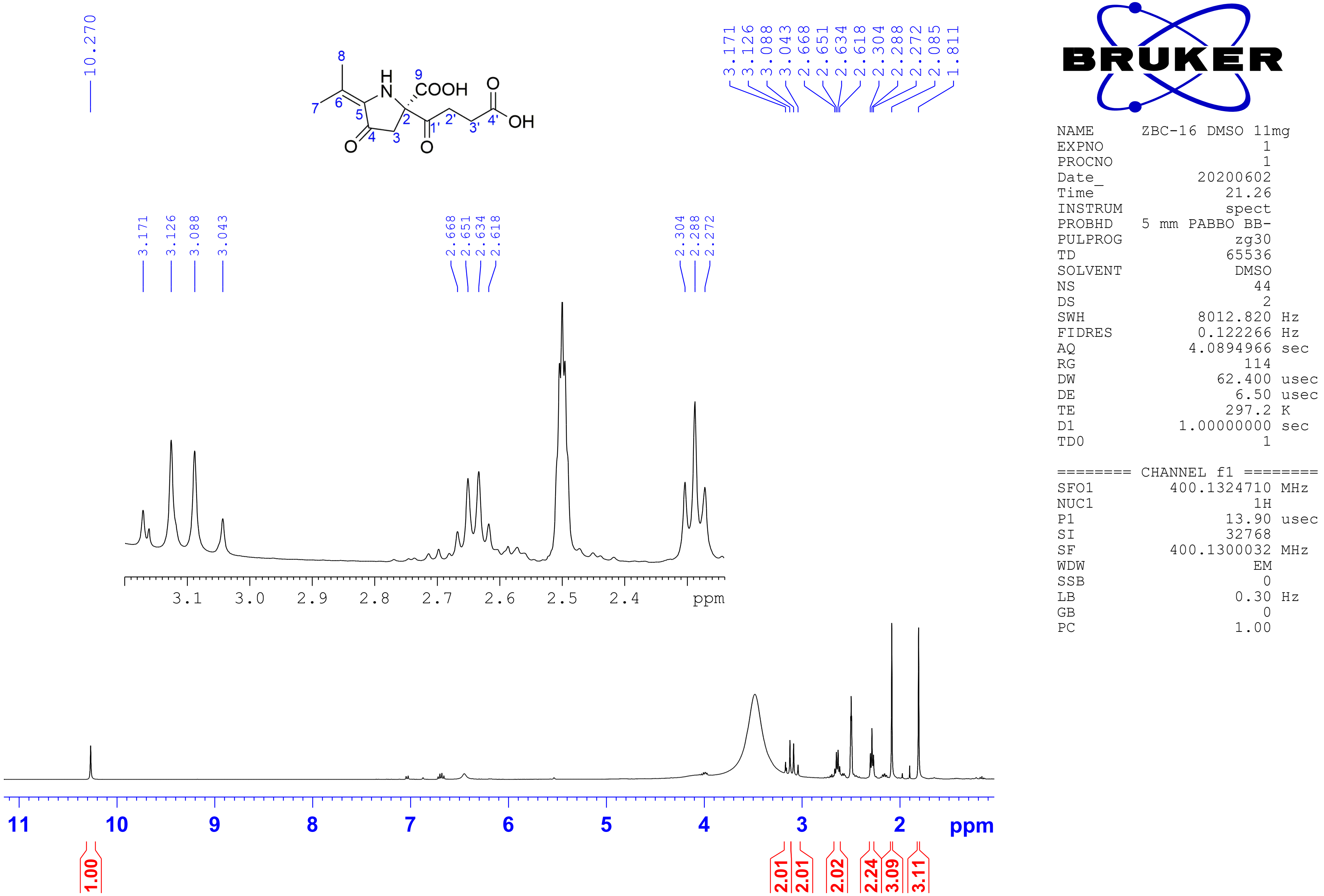

Figure S23. ^{13}C NMR spectrum of compound **4** in DMSO.

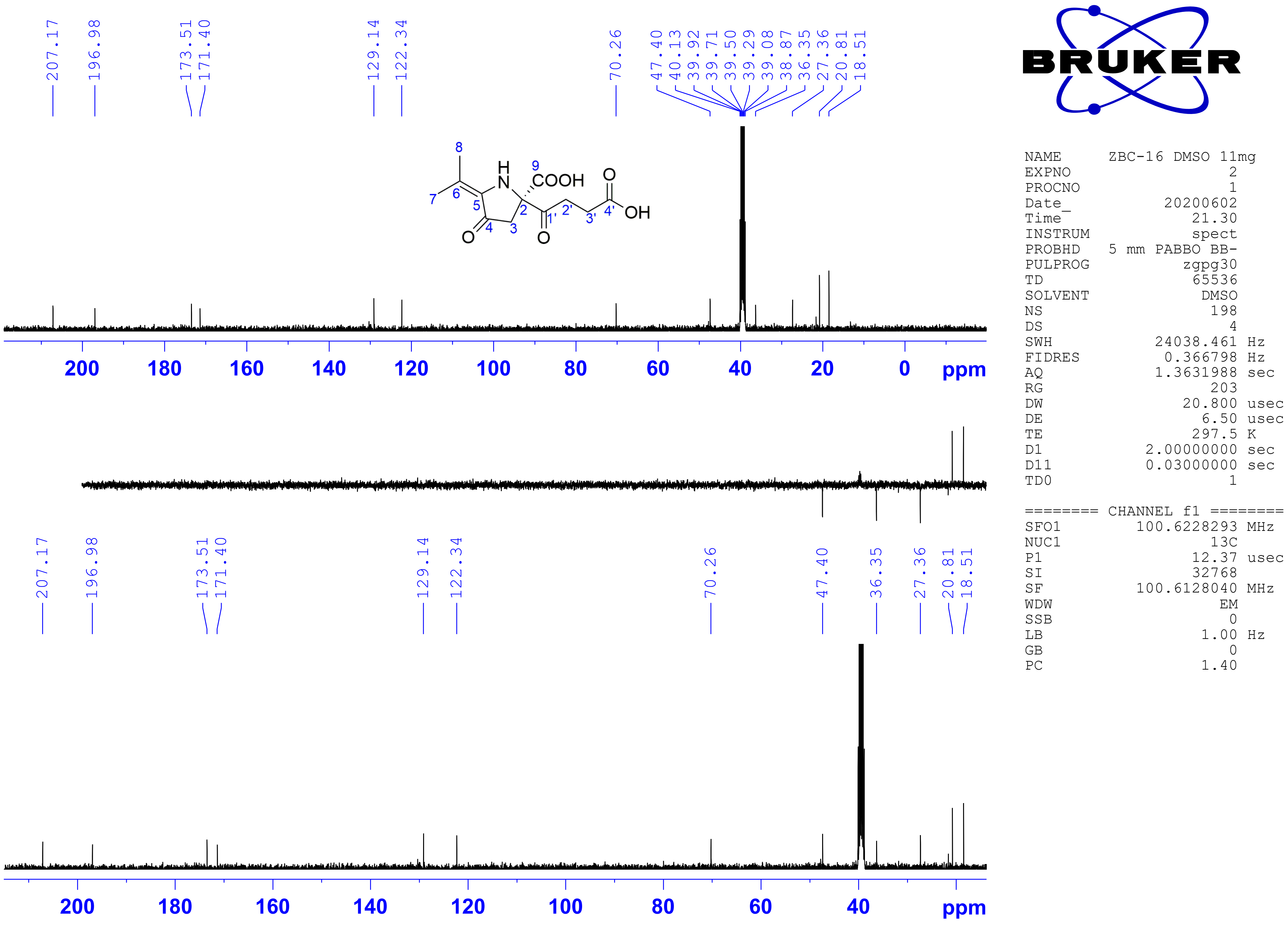

Figure S24. HSQC spectrum of compound **4** in DMSO.

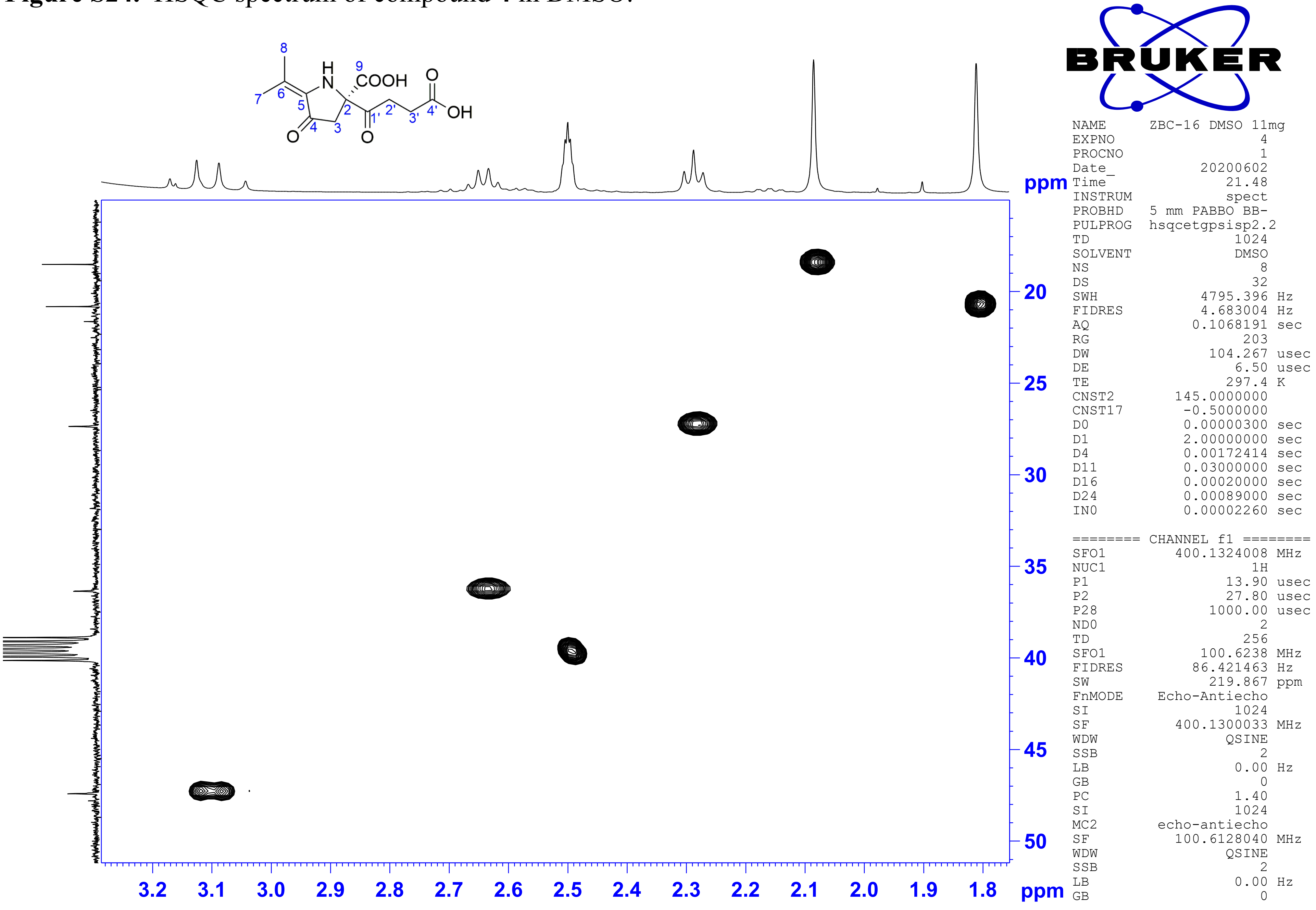

Figure S25. ^1H– ^1H COSY spectrum of compound **4** in DMSO.

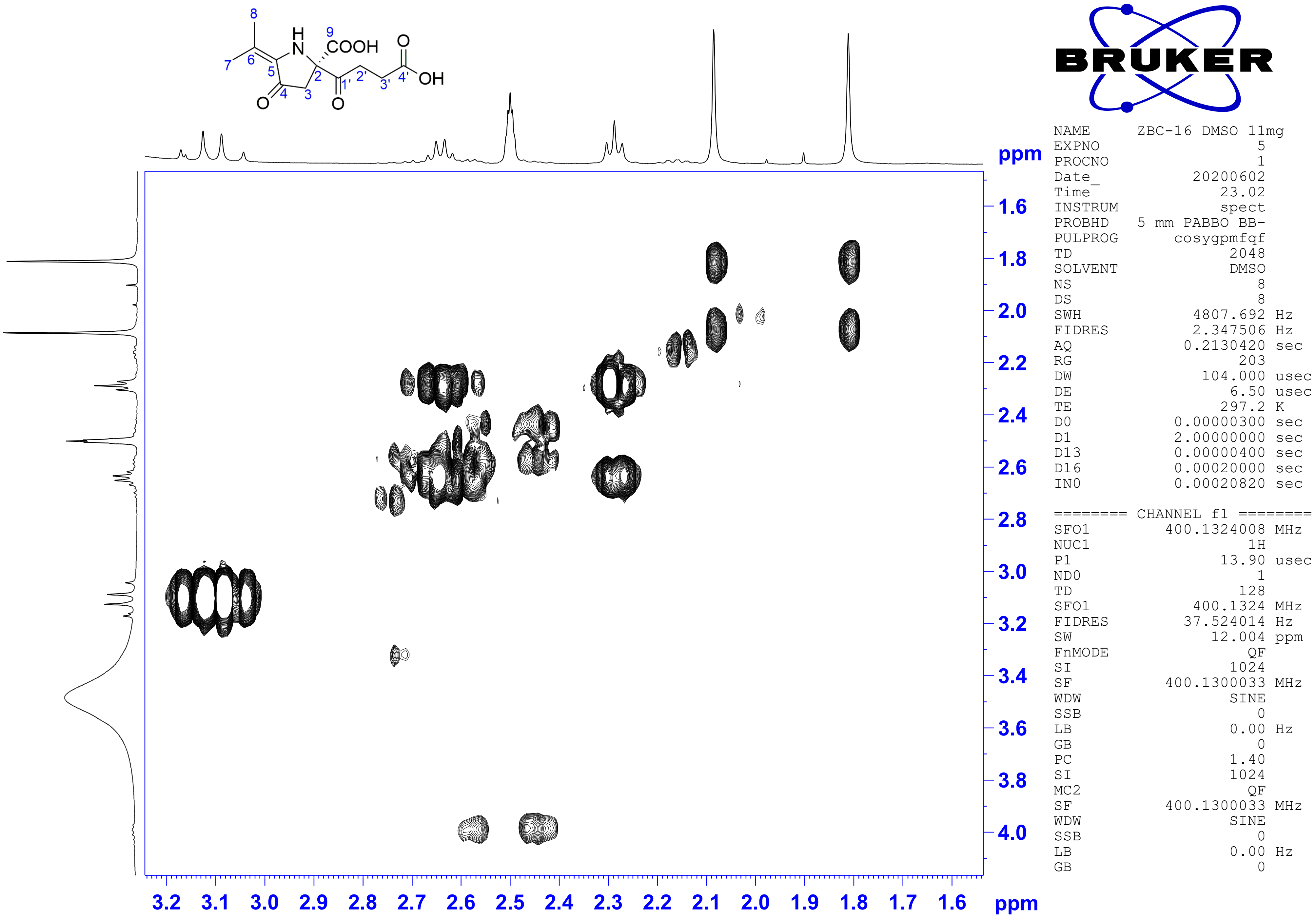

Figure S26. HMBC spectrum of compound **4** in DMSO.

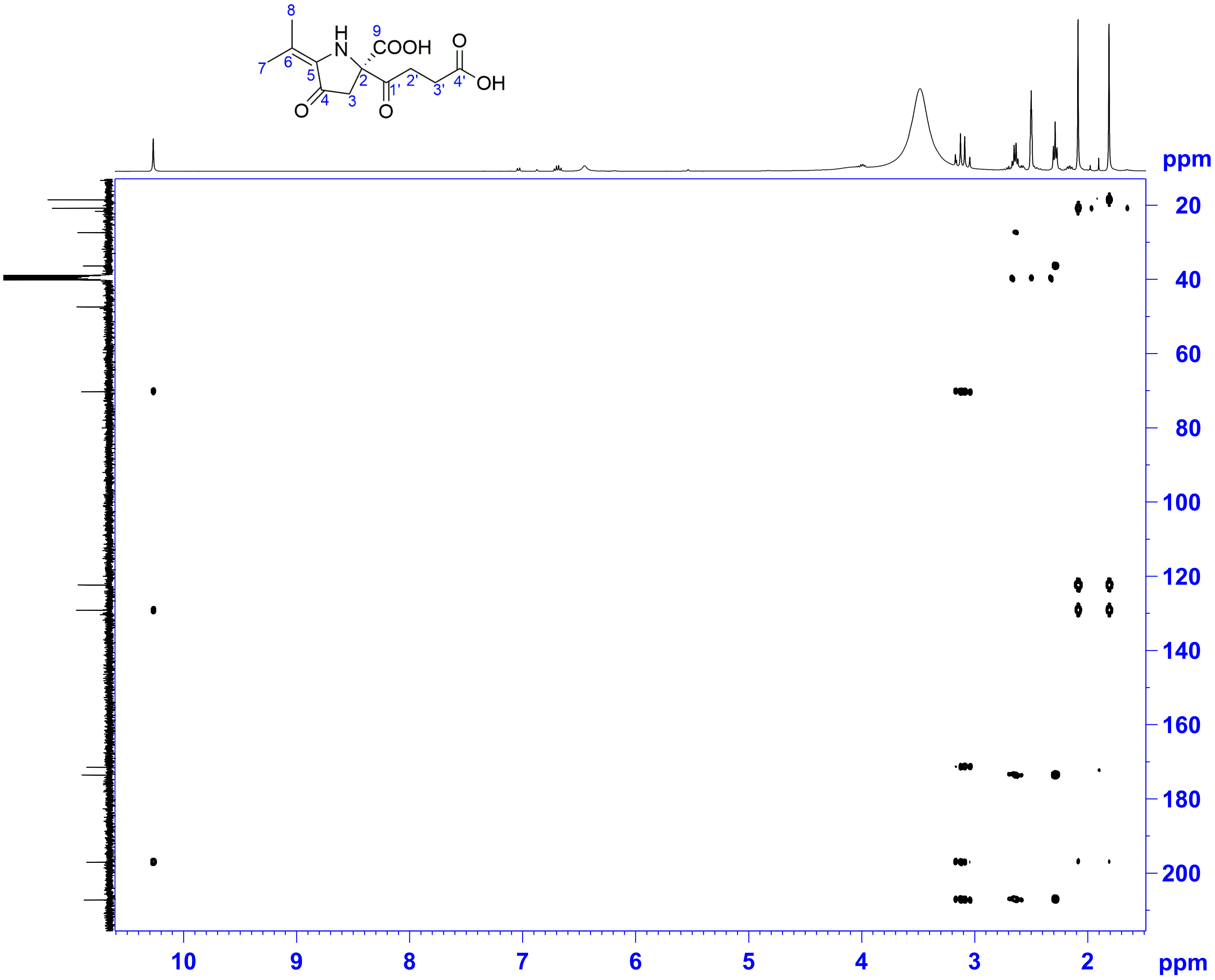

NAME	ZBC-16 DMSO 11mg
EXPNO	6
PROCNO	1
Date_	20200602
Time_	23.41
INSTRUM	spect
PROBHD	5 mm PABBO BB-
PULPROG	hmbcgpndqf
TD	4096
SOLVENT	DMSO
NS	16
DS	16
SWH	4807.692 Hz
FIDRES	1.173753 Hz
AQ	0.4260340 sec
RG	203
DW	104.000 usec
DE	6.50 usec
TE	296.9 K
CNST13	8.0000000
D0	0.00000300 sec
D1	1.50000000 sec
D6	0.06250000 sec
D16	0.00020000 sec
IN0	0.00002260 sec
======== CHANNEL f1 ========	
SFO1	400.1324008 MHz
NUC1	1H
P1	13.90 usec
P2	27.80 usec
ND0	2
TD	128
SFO1	100.6238 MHz
FIDRES	172.842926 Hz
SW	219.867 ppm
FnMODE	QF
SI	1024
SF	400.1300033 MHz
WDW	SINE
SSB	0
LB	0.00 Hz
GB	0
PC	1.40
SI	1024
MC2	QF
SF	100.6128040 MHz
WDW	SINE
SSB	0
LB	0.00 Hz
GB	0

Figure S27. NOESY spectrum of compound **4** in DMSO.

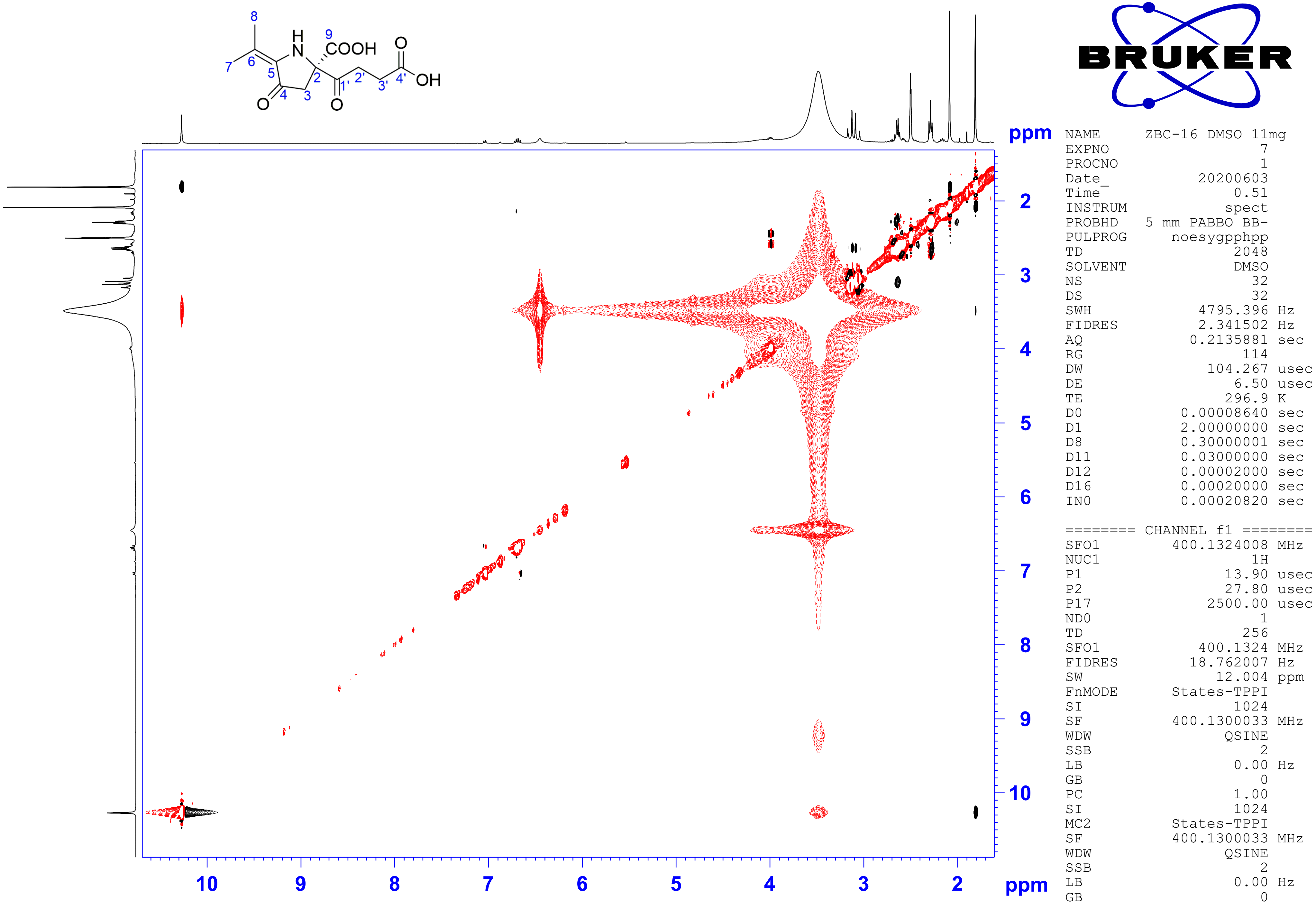

Figure S28. HRESIMS spectrum of compound **4**.

Elemental Composition Report

Single Mass Analysis

Tolerance = 40.0 mDa / DBE: min = -1.5, max = 50.0
Element prediction: Off
Number of isotope peaks used for i-FIT = 3

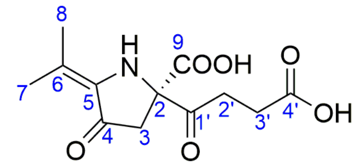

Chemical Formula: $C_{12}H_{15}NNaO_6^+$
Exact Mass: 292.0792

Monoisotopic Mass, Even Electron Ions
87 formula(e) evaluated with 10 results within limits (up to 50 best isotopic matches for each mass)
Elements Used:
C: 10-14 H: 0-40 N: 0-5 O: 0-7 Na: 1-1
ZBC-16 98 (0.390) Cm (51:262)
1: TOF MS ES+

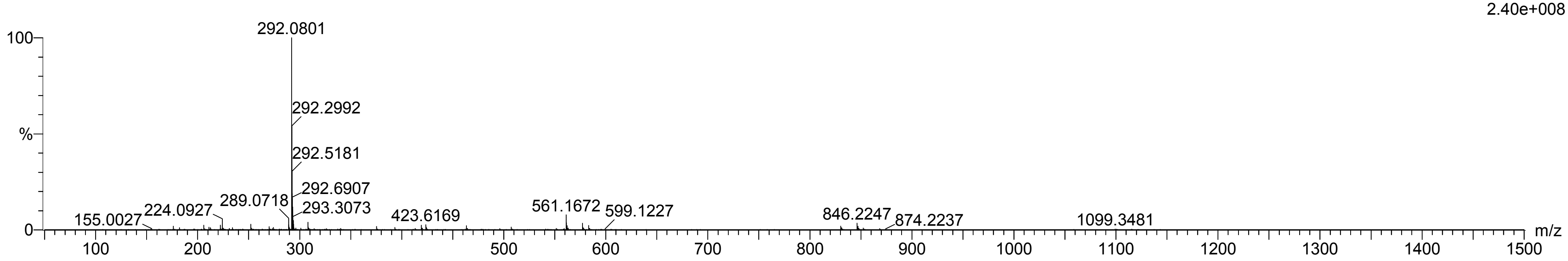

Minimum:				-1.5				
Maximum:		40.0	10.0	50.0				
Mass	Calc. Mass	mDa	PPM	DBE	i-FIT	Norm	Conf(%)	Formula
292.0801	292.0546	25.5	87.3	6.5	2895.5	0.777	46.00	C10 H11 N3 O6 Na
	292.1022	-22.1	-75.7	5.5	2896.1	1.426	24.03	C10 H15 N5 O4 Na
	292.0909	-10.8	-37.0	5.5	2896.9	2.181	11.29	C11 H15 N3 O5 Na
	292.0433	36.8	126.0	6.5	2897.1	2.381	9.24	C11 H11 N O7 Na
	292.0797	0.4	1.4	5.5	2897.3	2.615	7.32	C12 H15 N O6 Na
	292.1161	-36.0	-123.3	4.5	2899.3	4.613	0.99	C13 H19 N O5 Na
	292.0447	35.4	121.2	11.5	2899.9	5.194	0.56	C12 H7 N5 O3 Na
	292.0698	10.3	35.3	10.5	2900.5	5.860	0.29	C14 H11 N3 O3 Na
	292.0810	-0.9	-3.1	10.5	2900.8	6.076	0.23	C13 H11 N5 O2 Na
	292.1174	-37.3	-127.7	9.5	2902.1	7.462	0.06	C14 H15 N5 O Na

Figure S29. The proposed fragmentation scheme of compound **1** by ESI-QTrap-MS/MS.

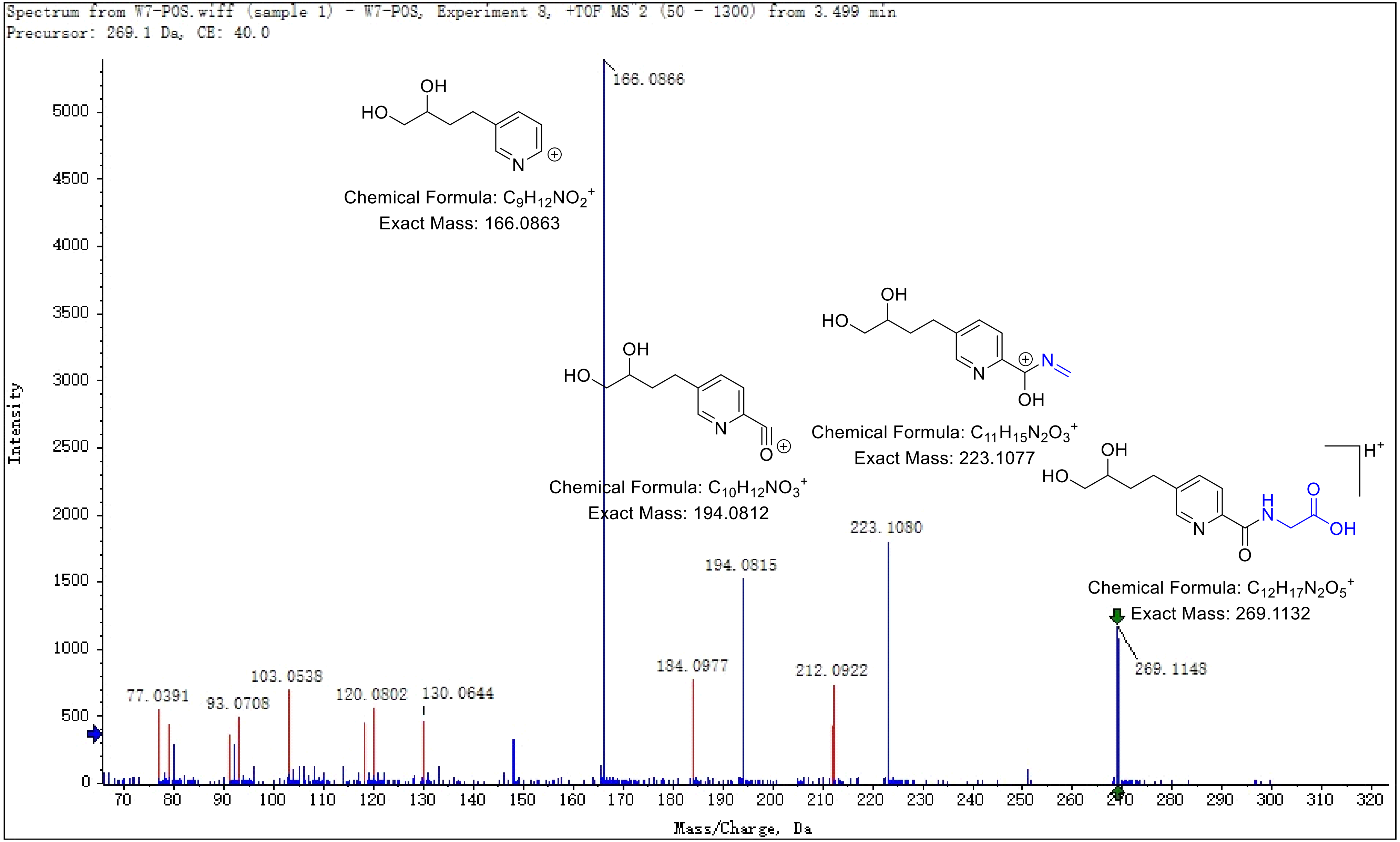

Figure S30. ^{1}H NMR spectrum of compound **5** in DMSO.

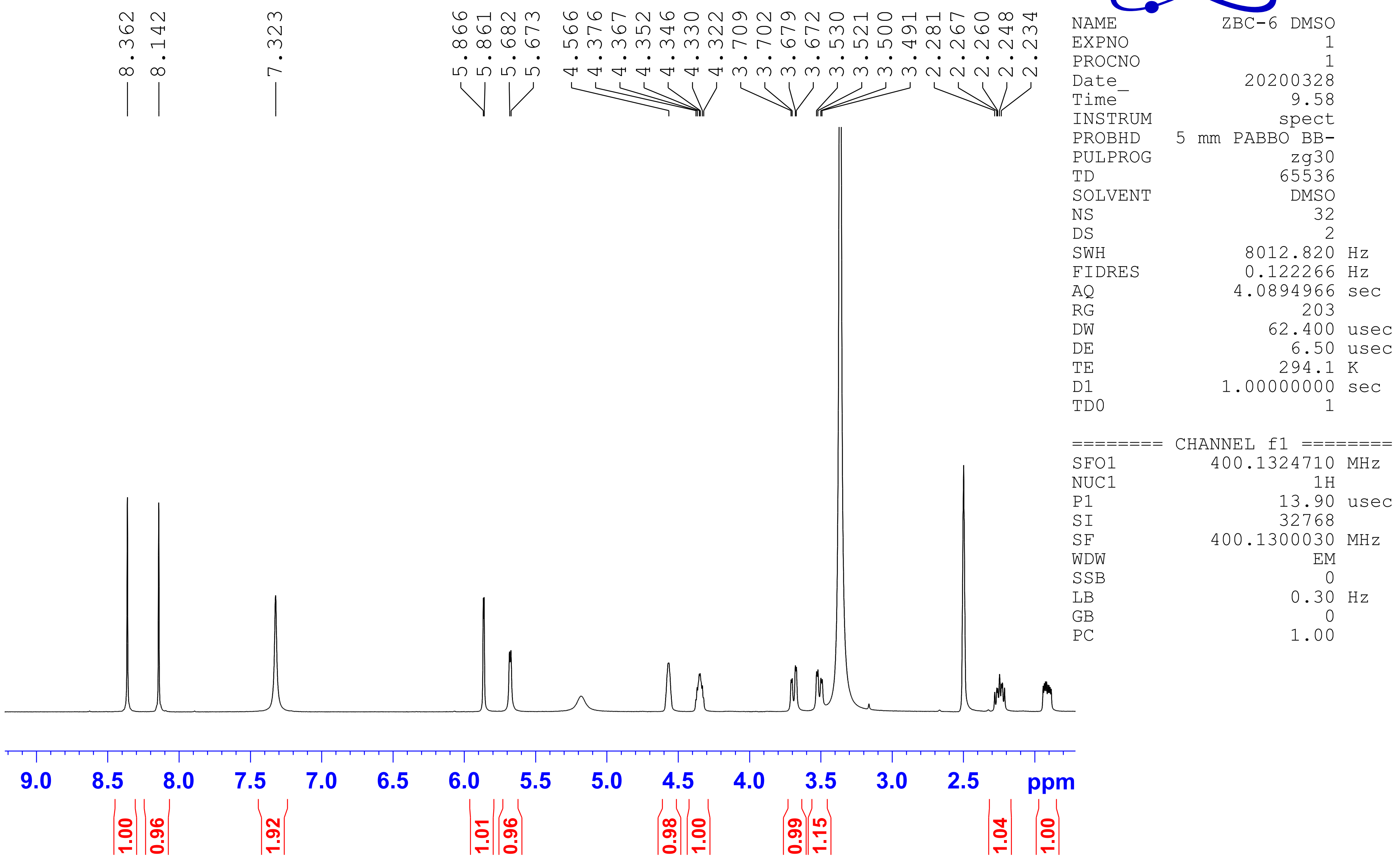

Figure S31. ^{1}H NMR spectrum of compound **6** in DMSO.

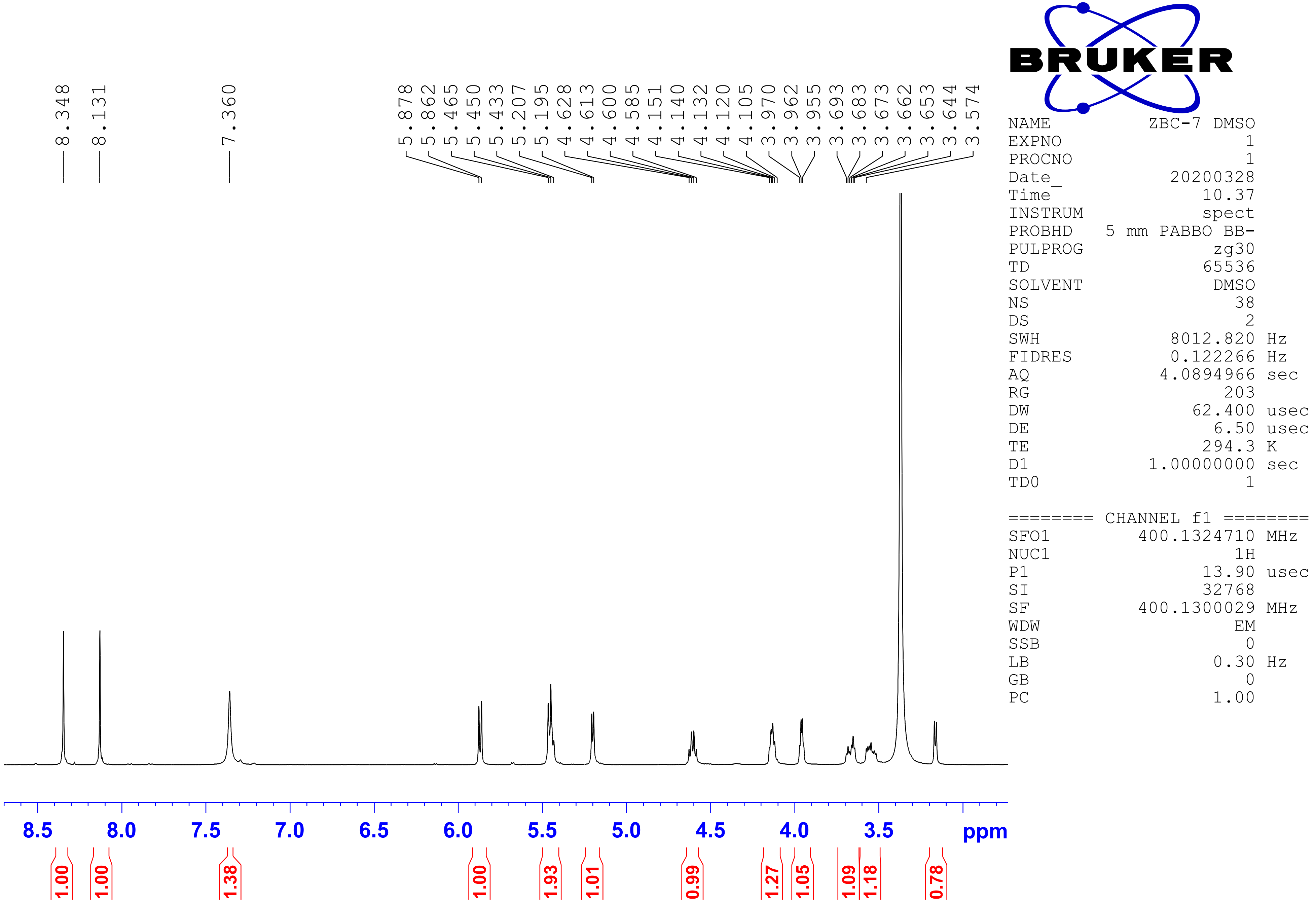

Figure S32. ^{1}H NMR spectrum of compound **7** in CD_3OD.

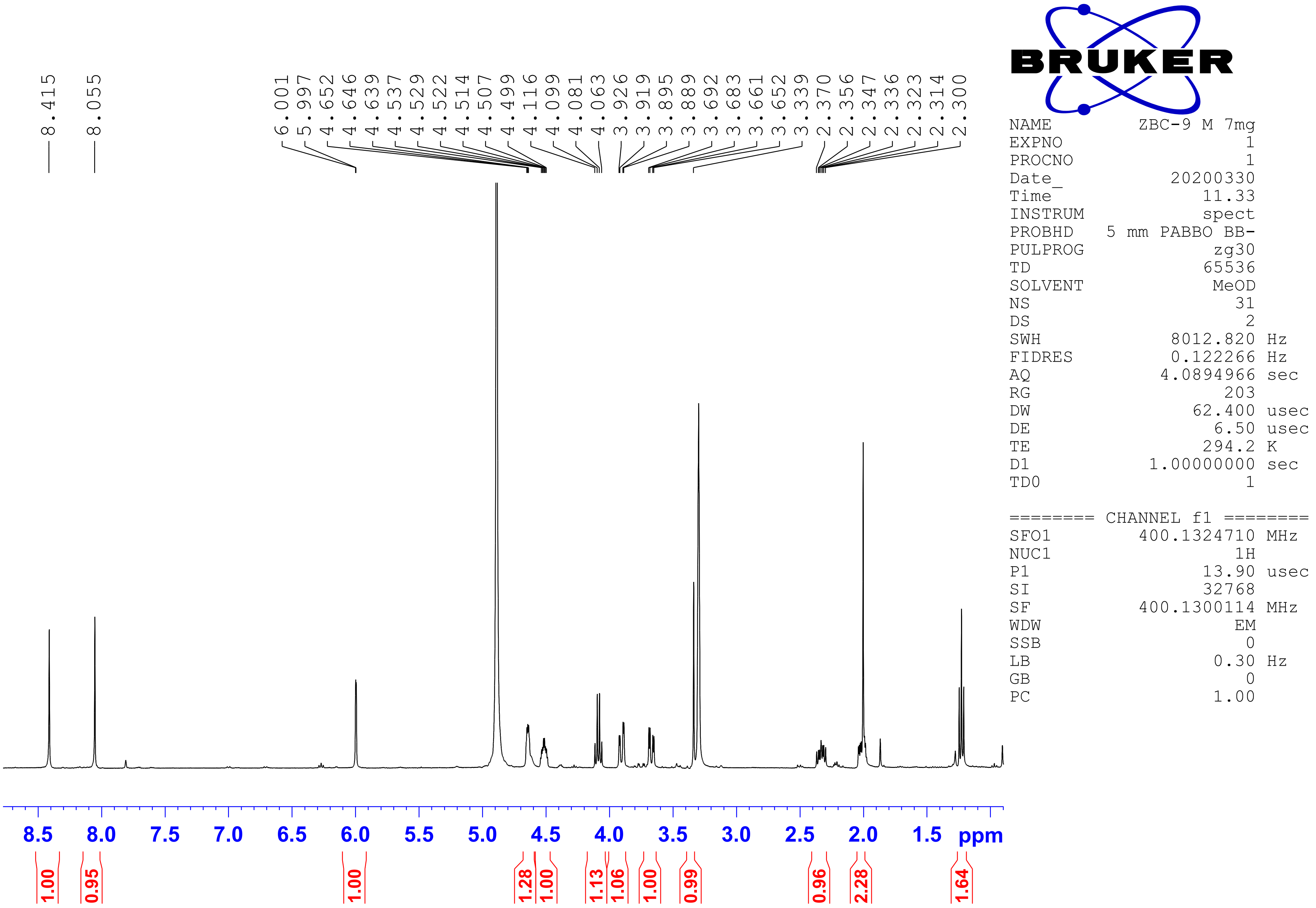

Figure S33. ^{1}H NMR spectrum of compound **8** in DMSO.

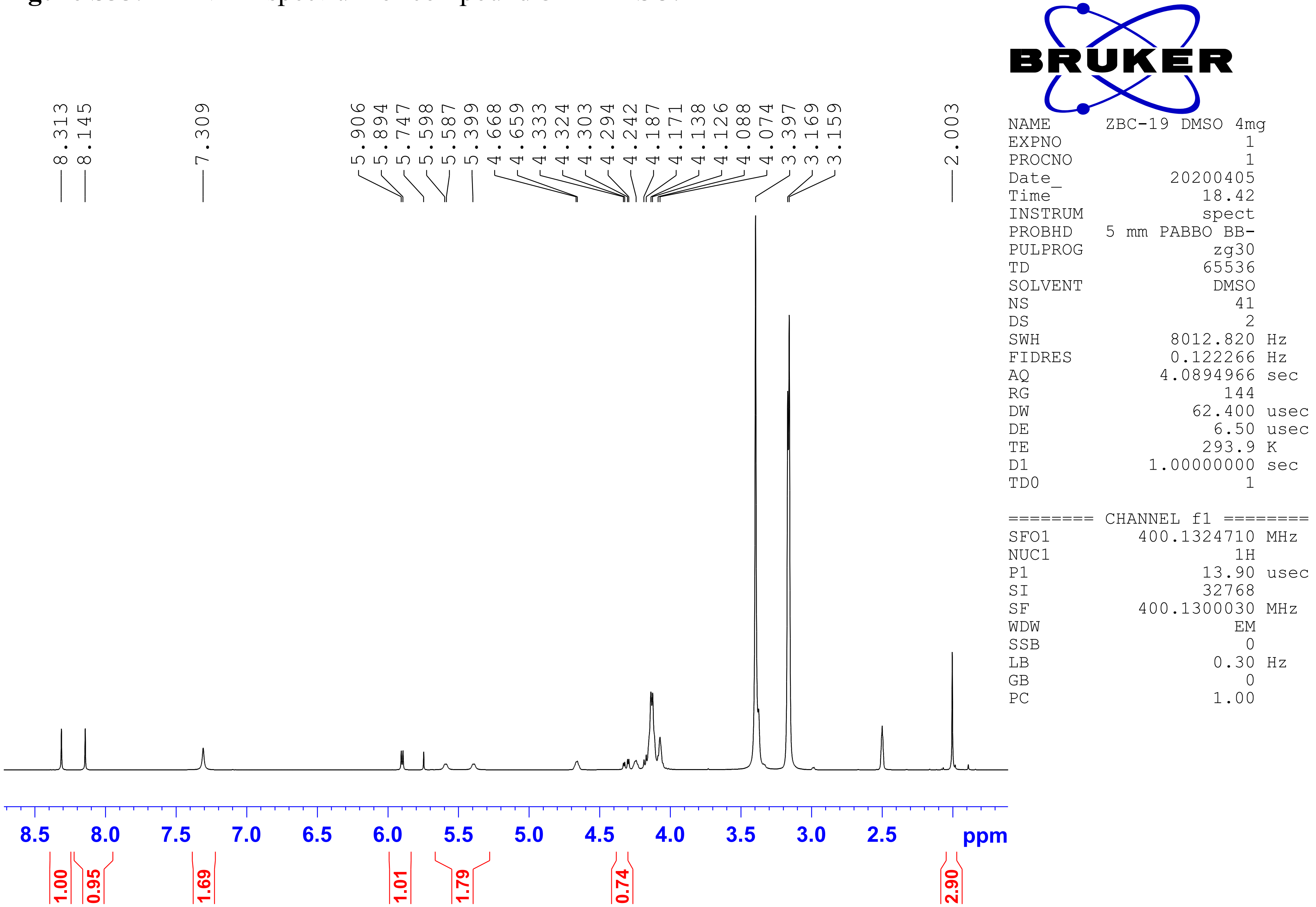

Figure S34. ^{1}H NMR spectrum of compound **9** in CD_3OD.

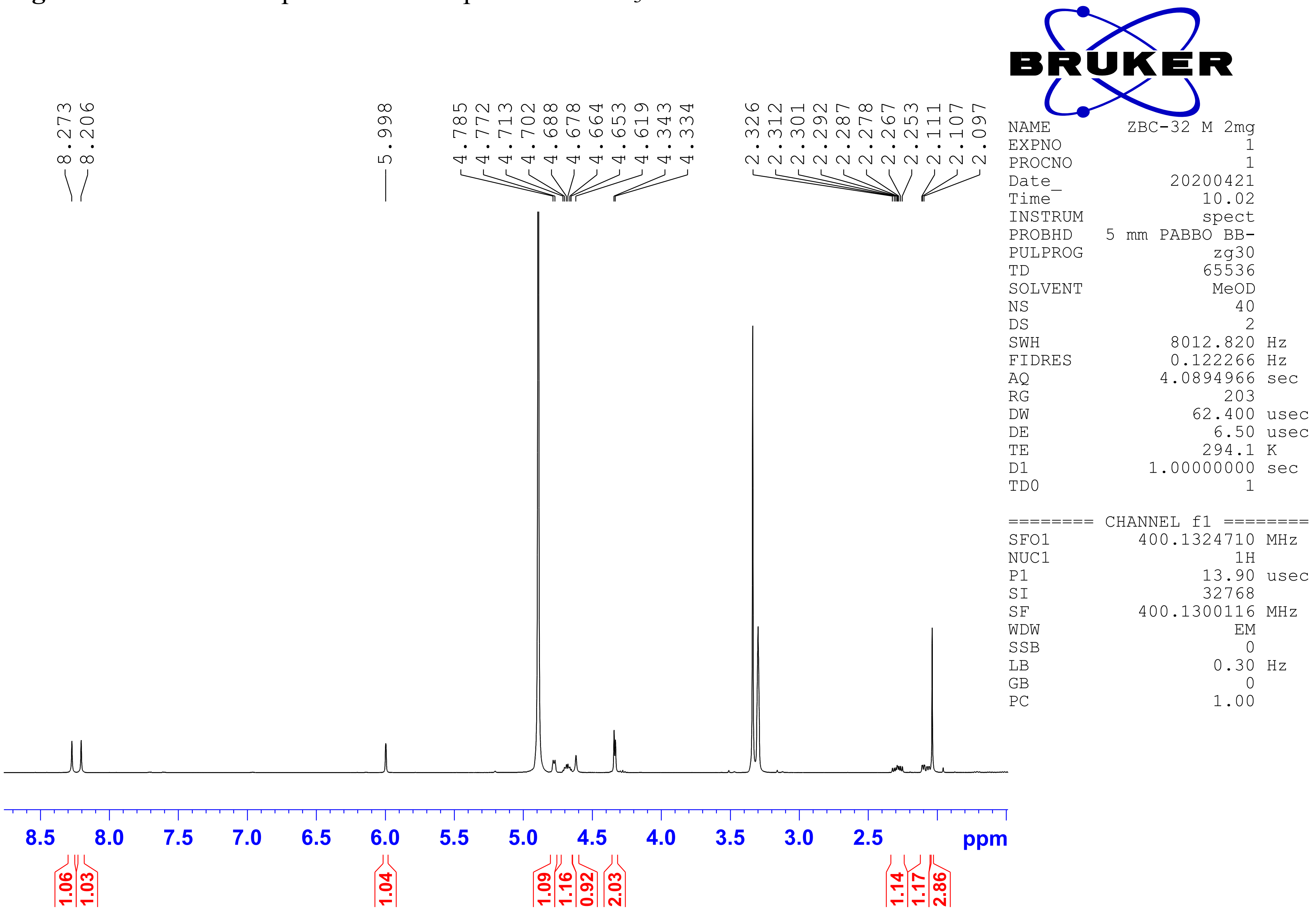

Figure S35. ^{1}H NMR spectrum of compound **10** in CD_3OD.

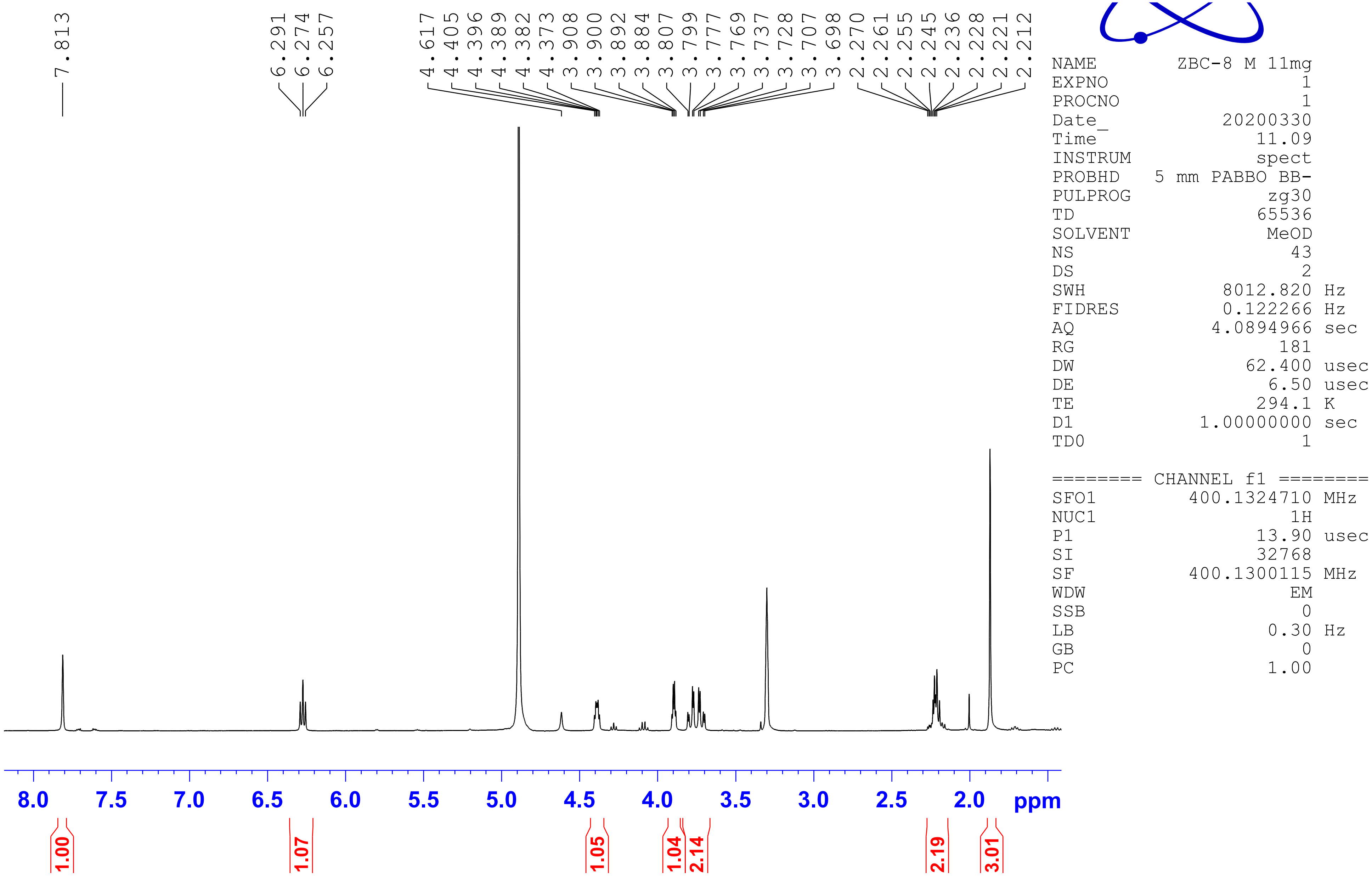

Figure S36. ^{1}H NMR spectrum of compound **11** in CD_3OD.

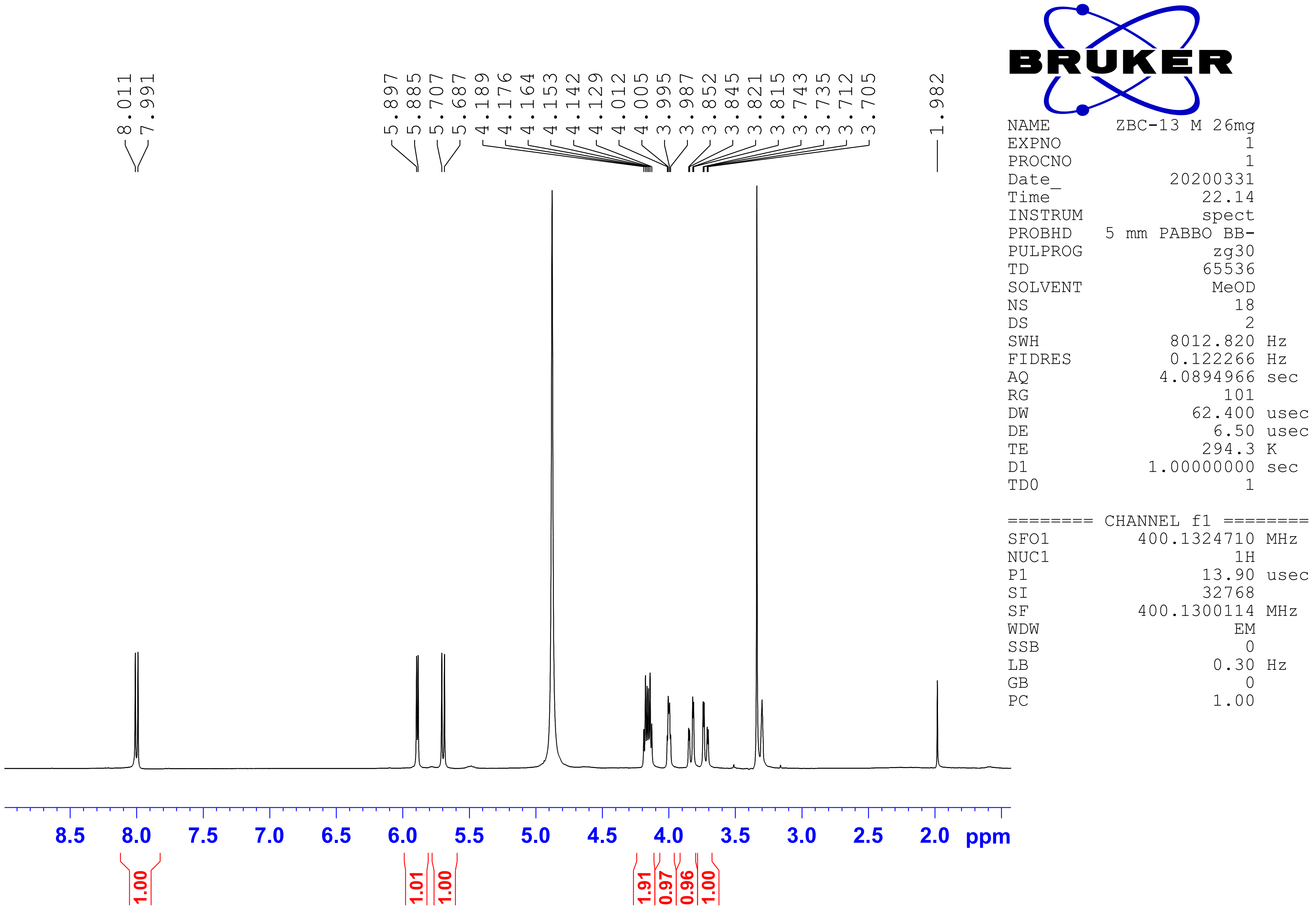

Figure S37. ^{1}H NMR spectrum of compound **12** in CD_3OD.

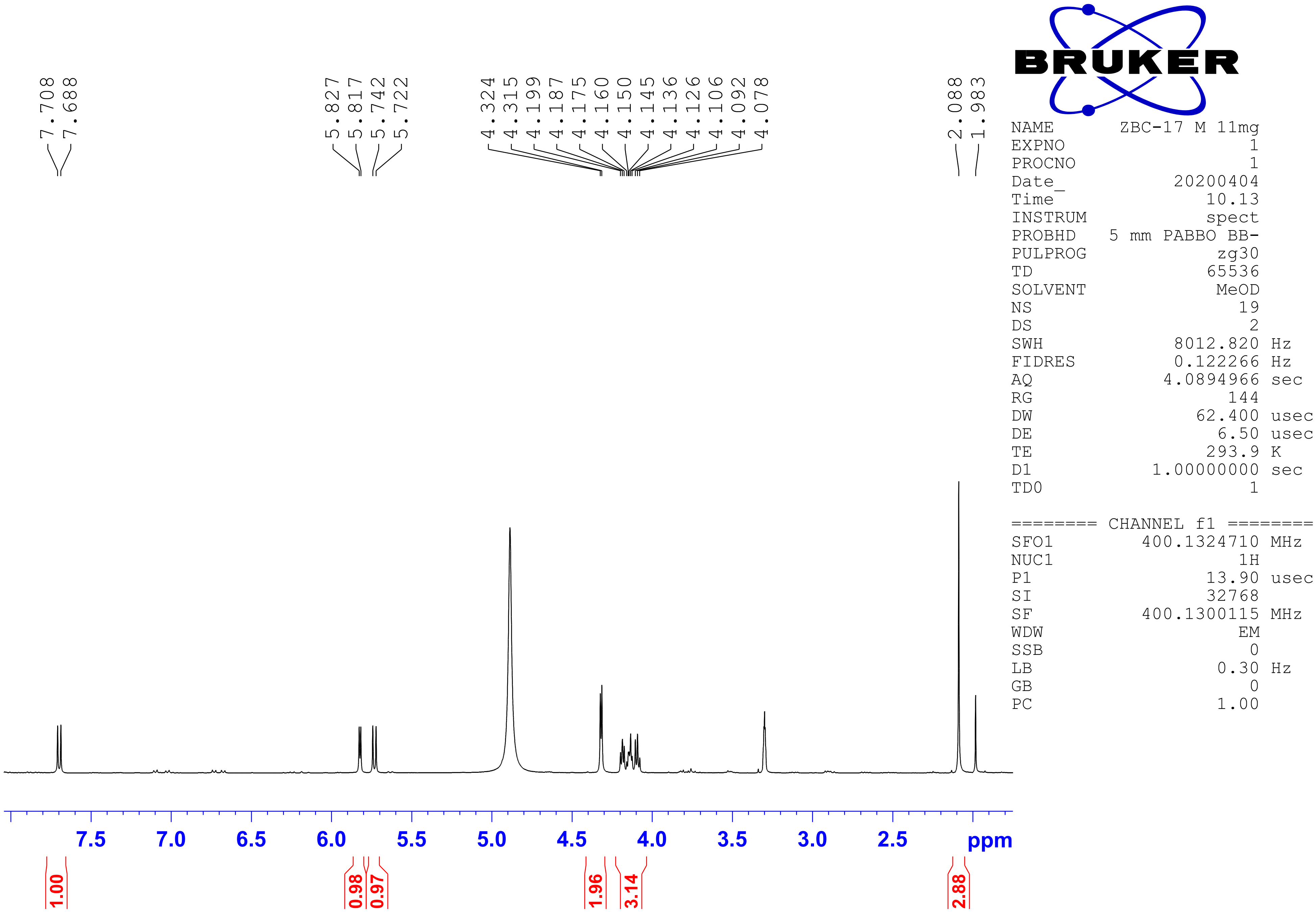

Figure S38. ^{1}H NMR spectrum of compound **13** in $CDCl_3$.

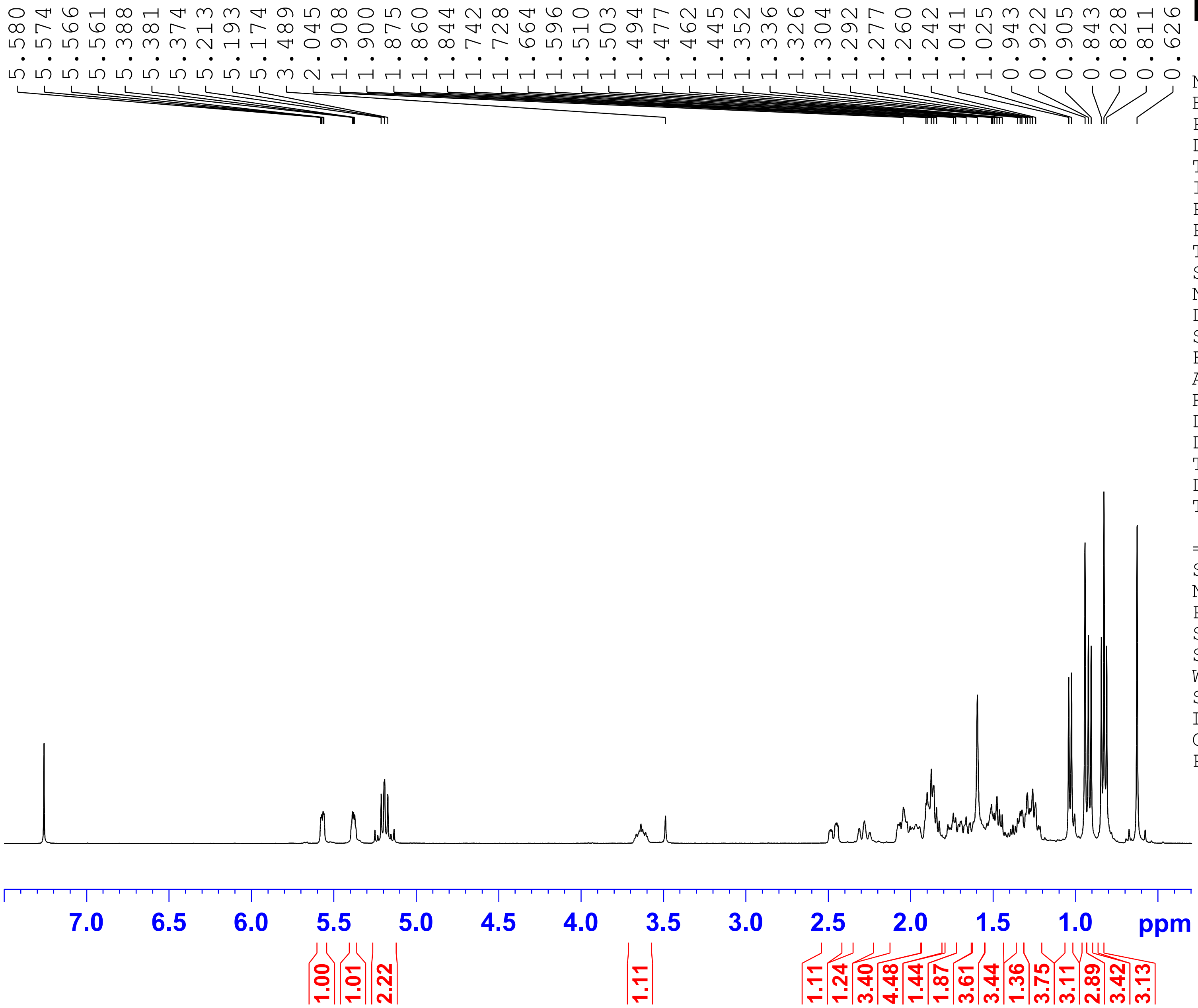

NAME	ZBC-21 CDCL3
EXPNO	1
PROCNO	1
Date_	20200408
Time	15.43
INSTRUM	spect
PROBHD	5 mm PABBO BB-
PULPROG	zg30
TD	65536
SOLVENT	CDCl3
NS	34
DS	2
SWH	8012.820 Hz
FIDRES	0.122266 Hz
AQ	4.0894966 sec
RG	203
DW	62.400 usec
DE	6.50 usec
TE	293.8 K
D1	1.00000000 sec
TD0	1
======== CHANNEL f1 ========	
SFO1	400.1324710 MHz
NUC1	1H
P1	13.90 usec
SI	32768
SF	400.1300097 MHz
WDW	EM
SSB	0
LB	0.30 Hz
GB	0
PC	1.00

Figure S39. ^{1}H NMR spectrum of compound **14** in CD_3OD.

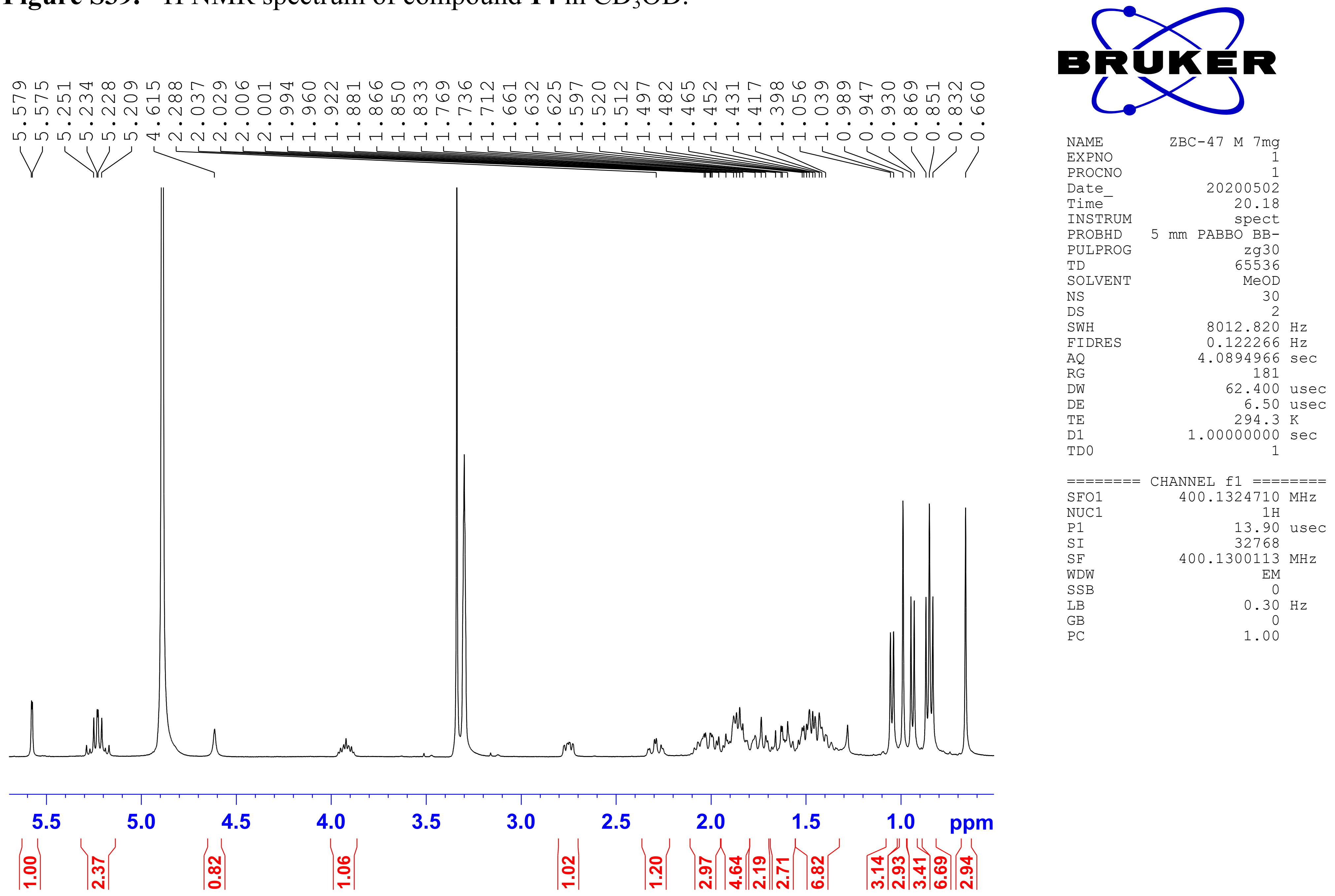

Figure S40. ^{1}H NMR spectrum of compound **15** in CD_3OD.

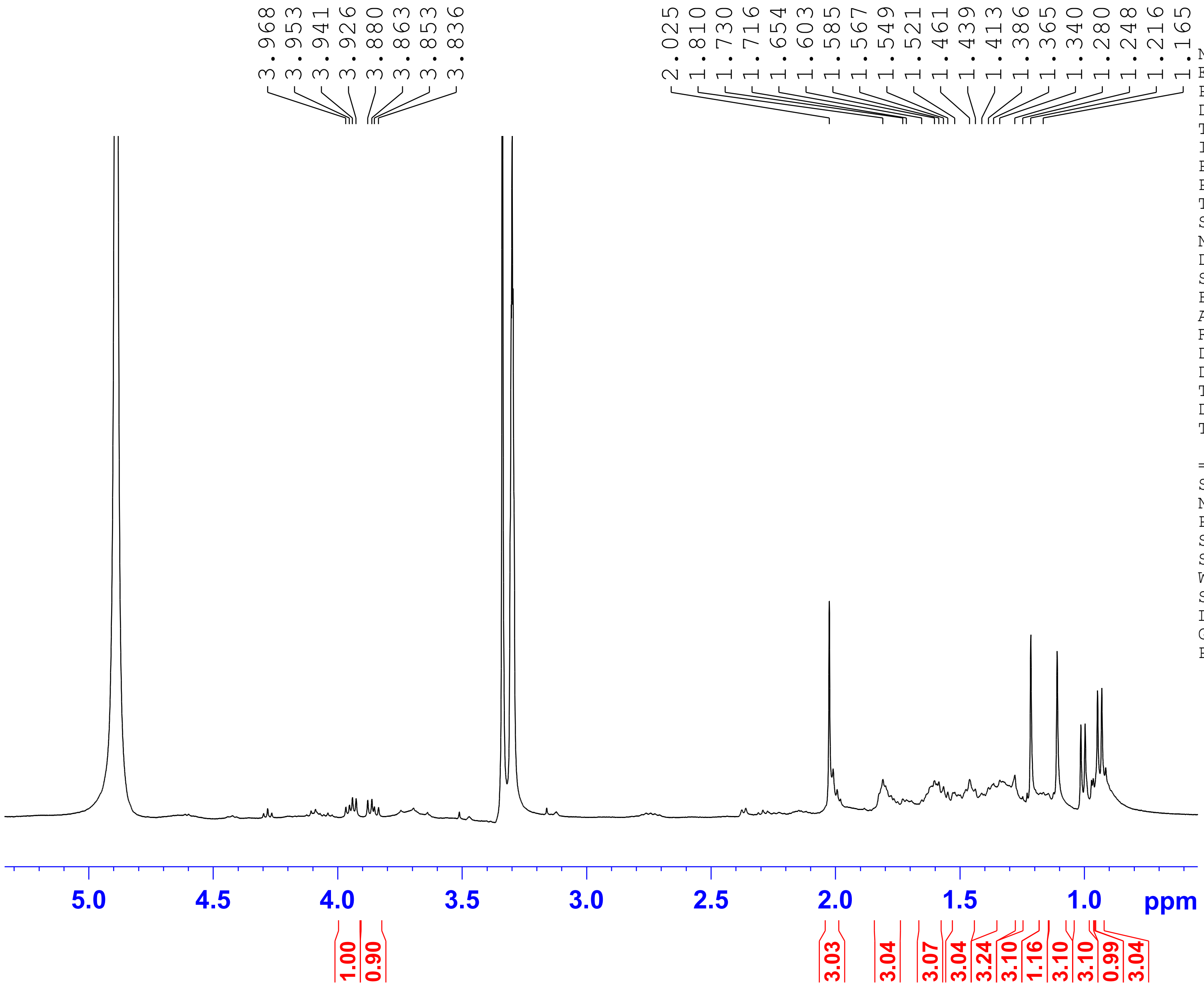

NAME	ZBC-40 M 2mg	
EXPNO	1	
PROCNO	1	
Date_	20200428	
Time	20.47	
INSTRUM	spect	
PROBHD	5 mm PABBO BB-	
PULPROG	zg30	
TD	65536	
SOLVENT	MeOD	
NS	54	
DS	2	
SWH	8012.820	Hz
FIDRES	0.122266	Hz
AQ	4.0894966	sec
RG	203	
DW	62.400	usec
DE	6.50	usec
TE	294.4	K
D1	1.00000000	sec
TD0	1	

======== CHANNEL f1 ========

SFO1	400.1324710	MHz
NUC1	1H	
P1	13.90	usec
SI	32768	
SF	400.1300116	MHz
WDW	EM	
SSB	0	
LB	0.30	Hz
GB	0	
PC	1.00	

Figure S41. ^{1}H NMR spectrum of compound **16** in CD_3OD.

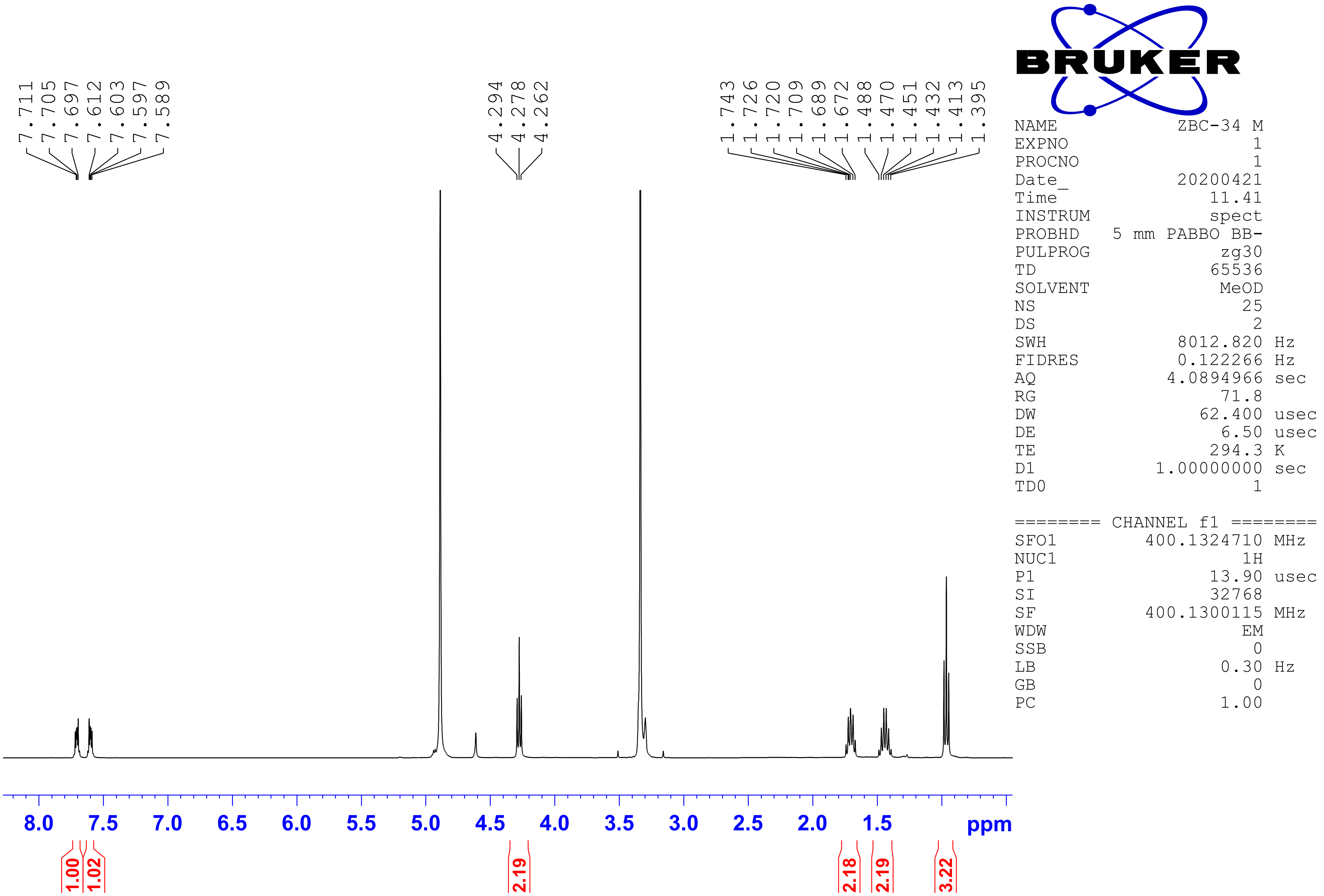

Figure S42. ^{1}H NMR spectrum of compound **17** in CD_3OD.

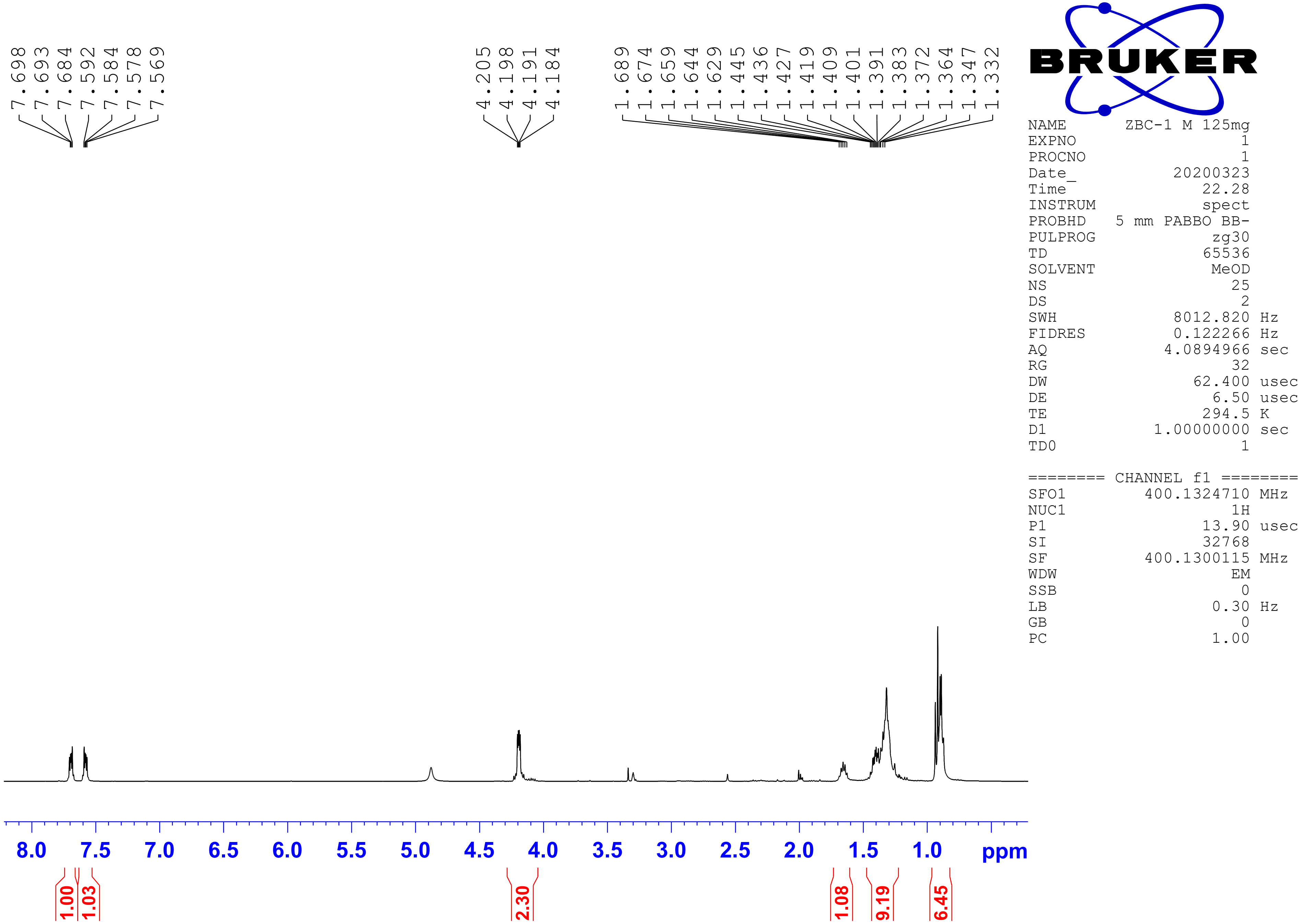

Figure S43. ^{1}H NMR spectrum of compound **18** in CD_3OD.

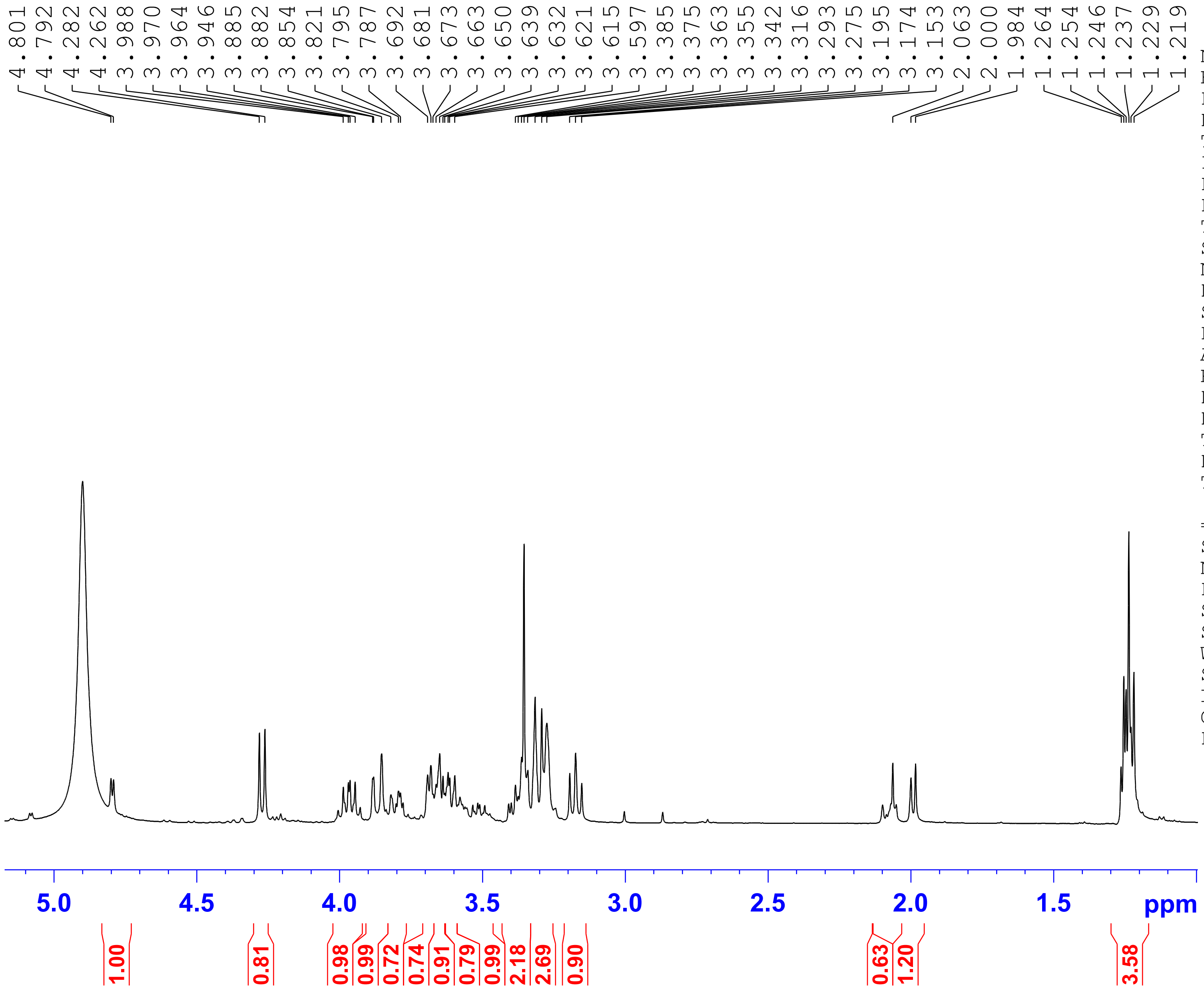

Figure S44. ^{1}H NMR spectrum of compound **19** in CD_3OD.

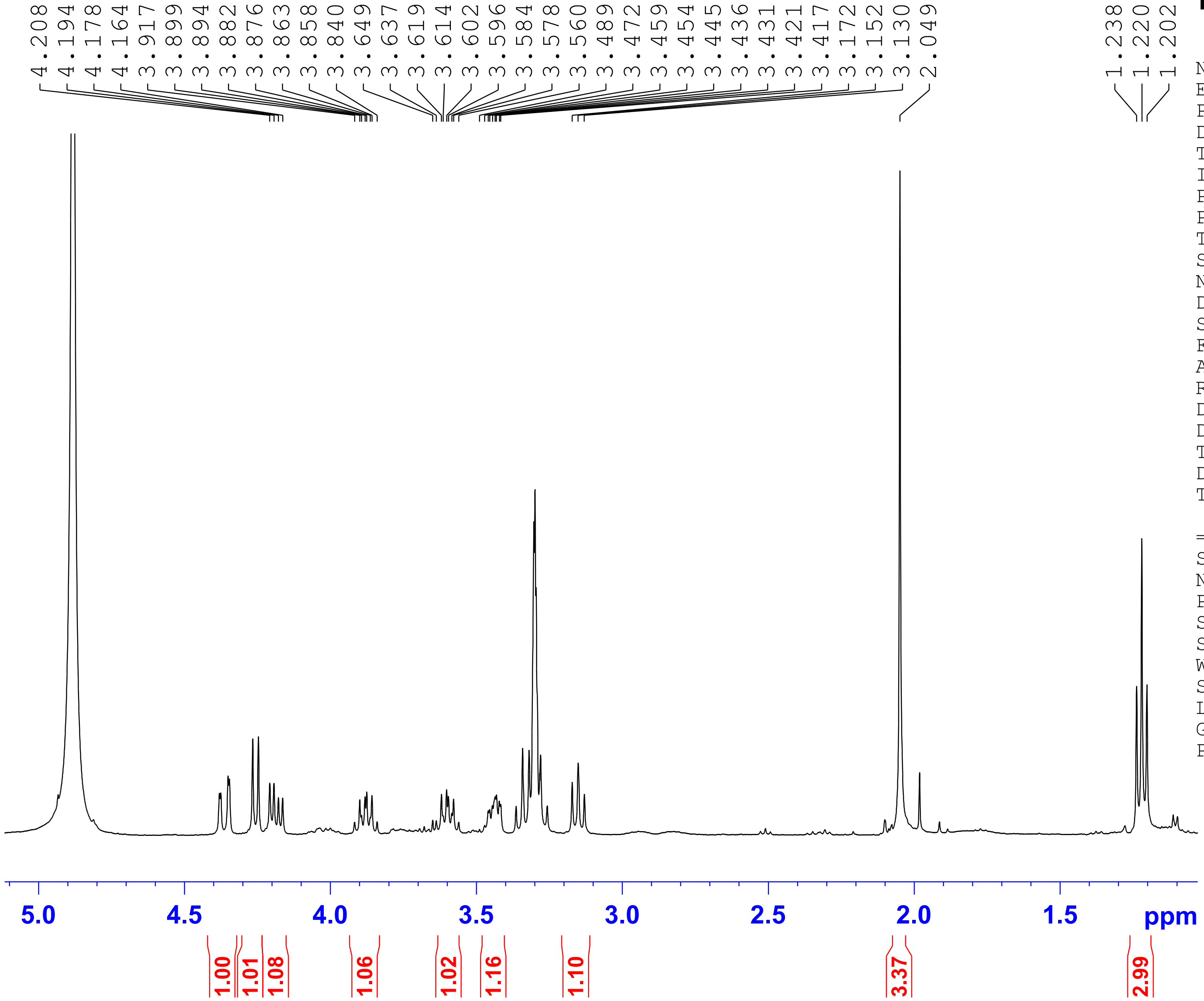

NAME	ZBC-15 M 4mg
EXPNO	1
PROCNO	1
Date_	20200402
Time_	21.43
INSTRUM	spect
PROBHD	5 mm PABBO BB-
PULPROG	zg30
TD	65536
SOLVENT	MeOD
NS	38
DS	2
SWH	8012.820 Hz
FIDRES	0.122266 Hz
AQ	4.0894966 sec
RG	181
DW	62.400 usec
DE	6.50 usec
TE	294.4 K
D1	1.00000000 sec
TD0	1

======== CHANNEL f1 ========

SFO1	400.1324710 MHz
NUC1	1H
P1	13.90 usec
SI	32768
SF	400.1300119 MHz
WDW	EM
SSB	0
LB	0.30 Hz
GB	0
PC	1.00

Figure S45. ^{1}H NMR spectrum of compound **20** in CD_3OD.

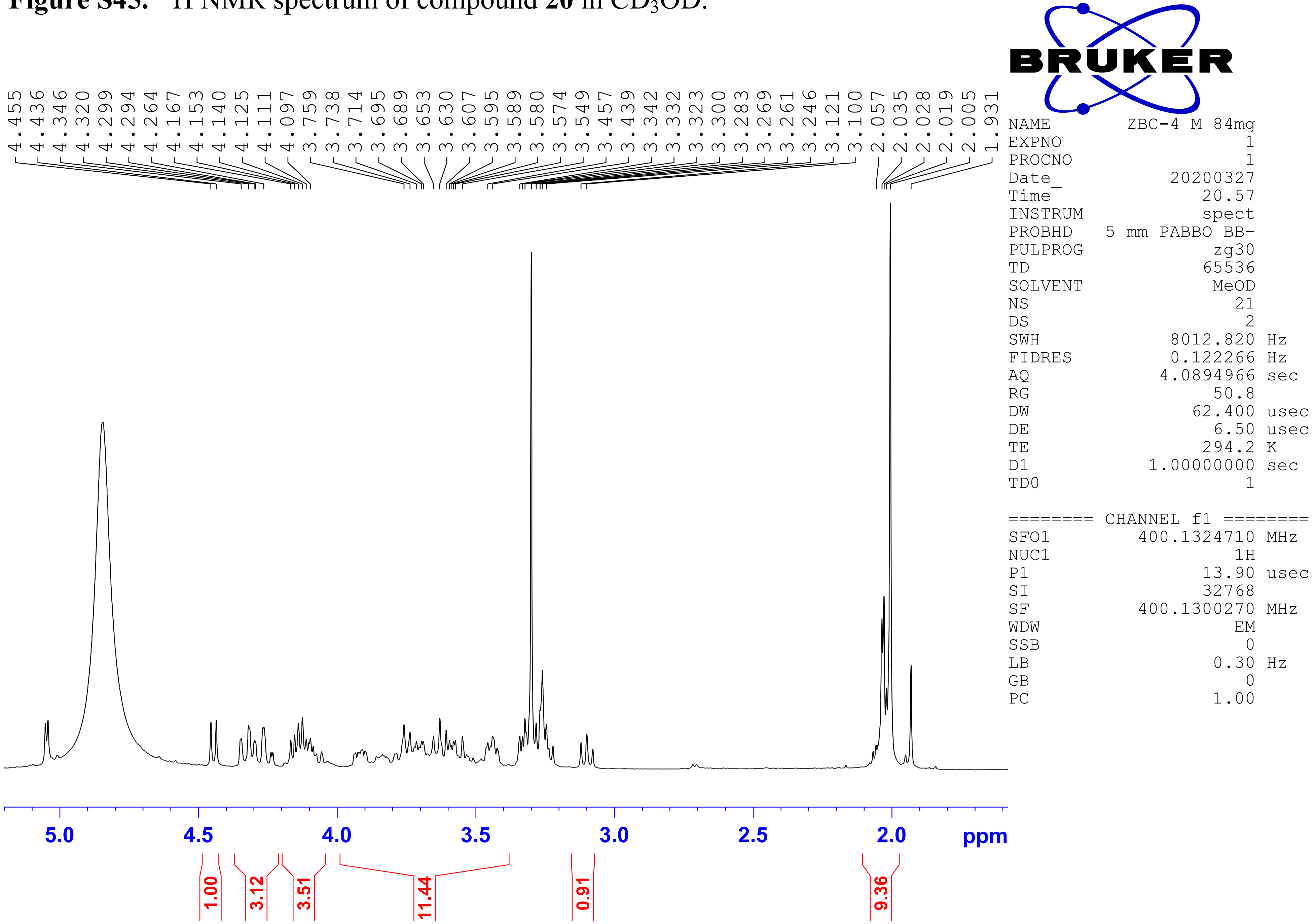

Figure S46. ^{1}H NMR spectrum of compound **21** in CD_3OD.

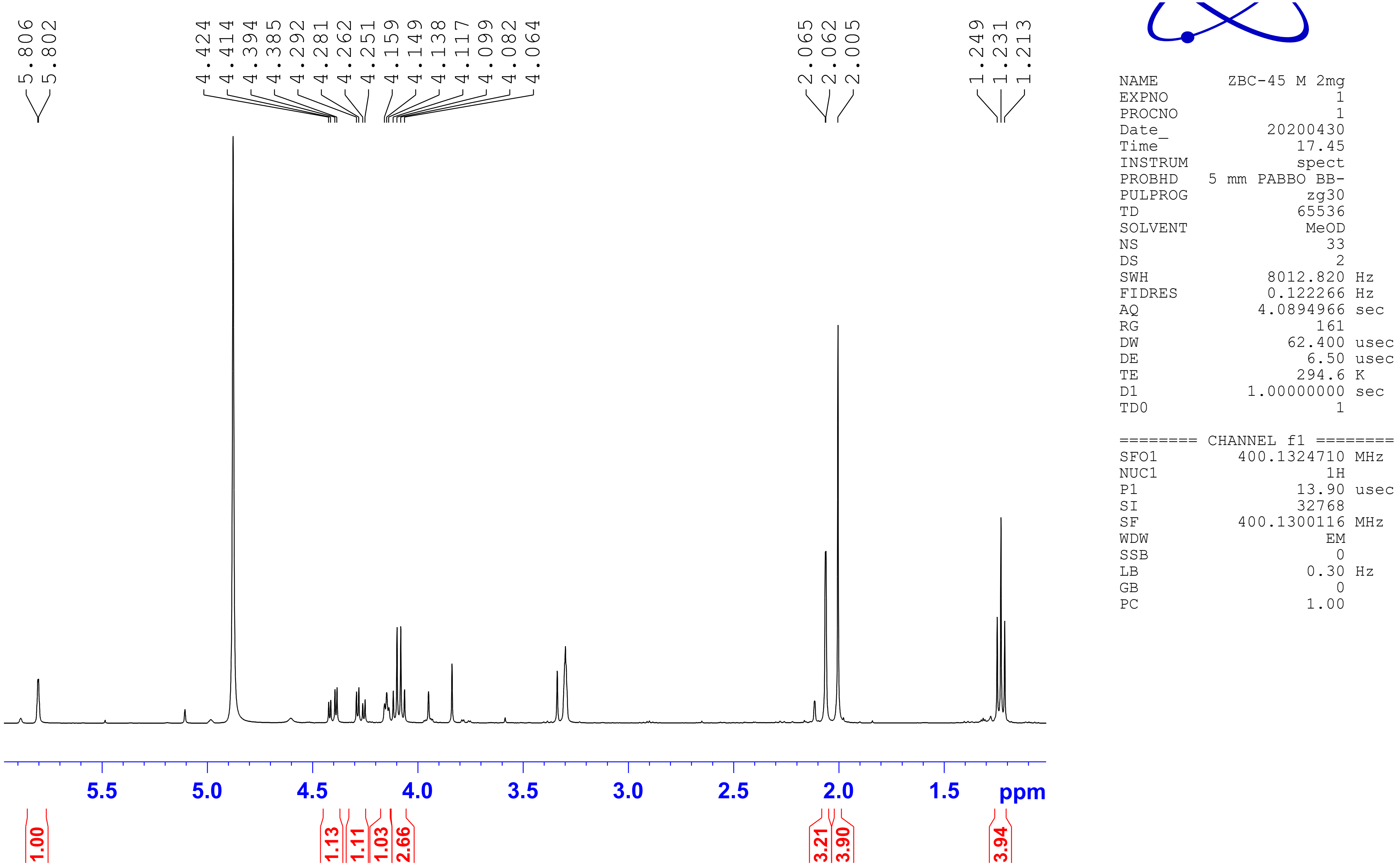

Figure S47. ^{1}H NMR spectrum of compound **22** in CD_3OD.

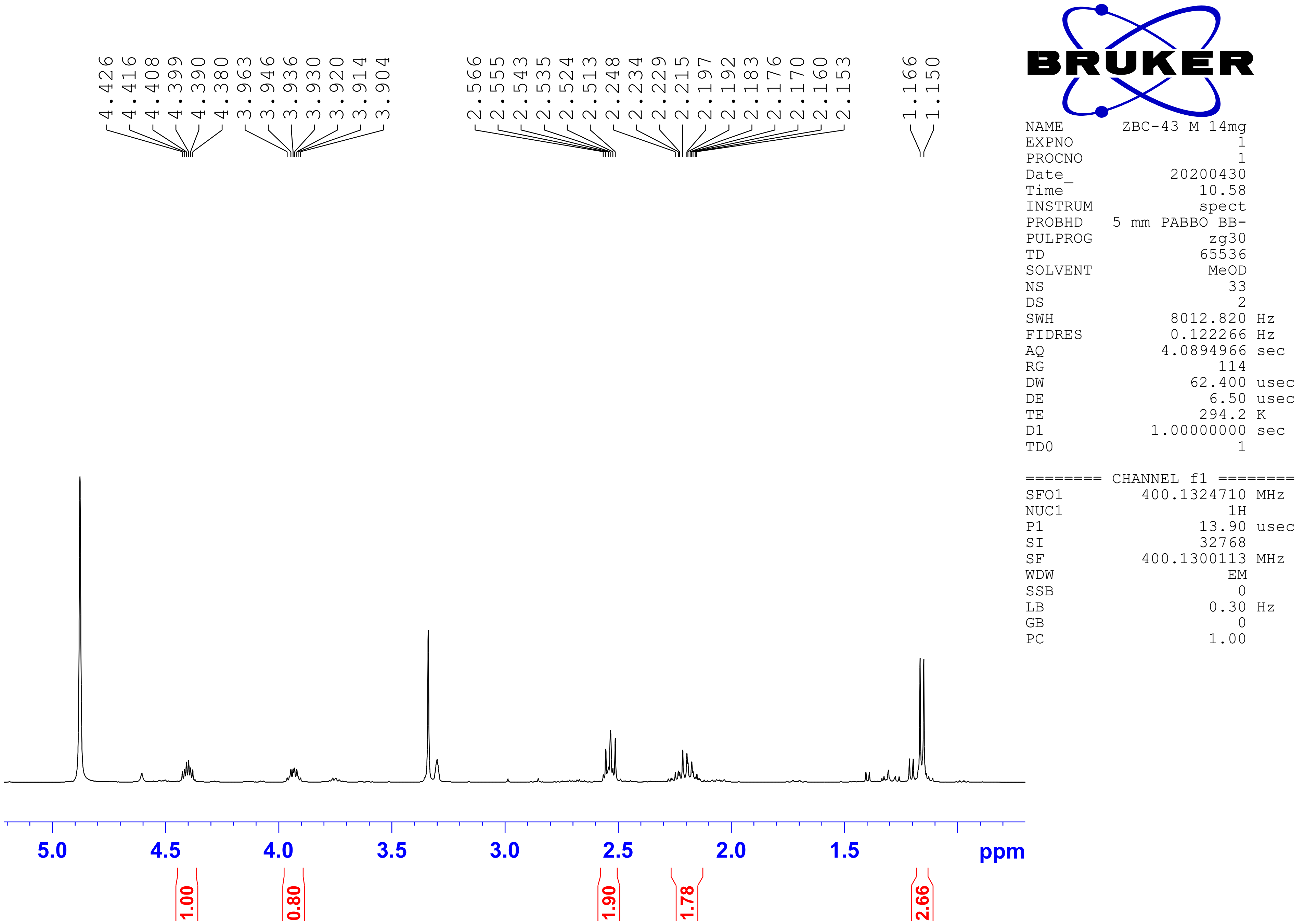

Figure S48. Gibbs free energy and equilibrium populations of low-energy conformers of **1** in ECD calculations.

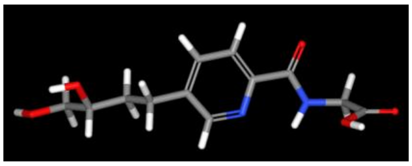

(10*R*-**3A**)

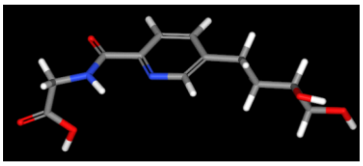

(10*R*-**3B**)

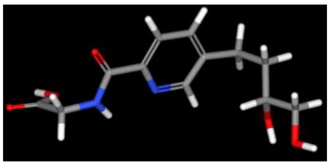

(10*R*-**3C**)

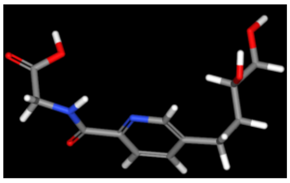

(10*R*-**3D**)

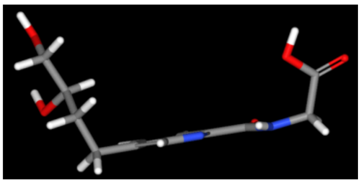

(10*R*-**3E**)

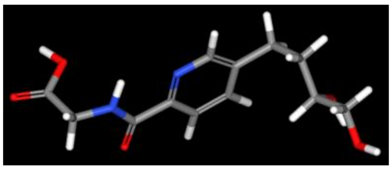

(10*R*-**3F**)

Conformers	Energy (Hartree)	Energy (kcal/mol)	Population (%)	Theory level	Solvent
10*R* -**3A**	-952.8454	-597919.54	20.4	B3LYP/6-311G(d,p)	Methanol
10*R* -**3B**	-952.8454	-597919.52	19.8	B3LYP/6-311G(d,p)	Methanol
10*R* -**3C**	-952.8451	-597919.37	15.3	B3LYP/6-311G(d,p)	Methanol
10*R* -**3D**	-952.8451	-597919.37	15.3	B3LYP/6-311G(d,p)	Methanol
10*R* -**3E**	-952.8451	-597919.34	14.6	B3LYP/6-311G(d,p)	Methanol
10*R* -**3F**	-952.8451	-597919.34	14.6	B3LYP/6-311G(d,p)	Methanol

Figure S49. Gibbs free energy and equilibrium populations of low-energy conformers of **3** in ECD calculations.

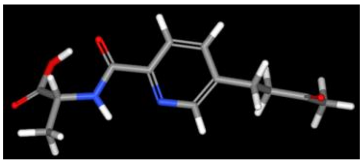
1'*S*-3A

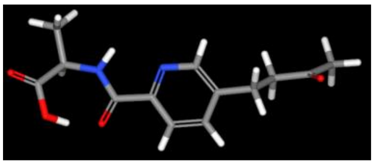
1'*S*-3B

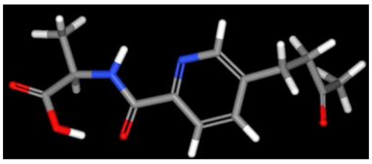
1'*S*-3C

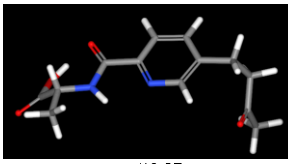
1'*S*-3D

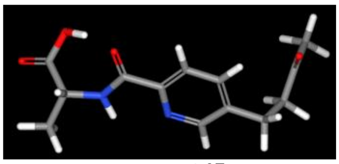
1'*S*-3E

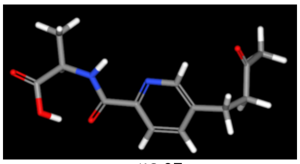
1'*S*-3F

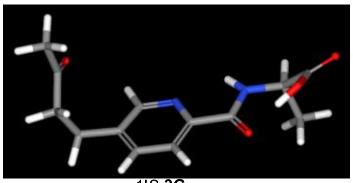
1'*S*-3G

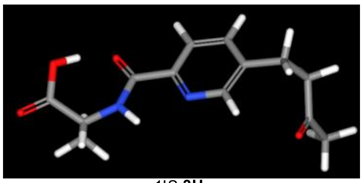
1'*S*-3H

Conformers	Energy (Hartree)	Energy (kcal/mol)	Population (%)	Theory level	Solvent
**1′*S*-3A**	-915.7345	-574632.10	23.1	B3LYP/6-311G(d,p)	Methanol
**1′*S* -3B**	-915.7345	-574632.07	22.0	B3LYP/6-311G(d,p)	Methanol
**1′*S* -3C**	-915.7340	-574631.78	13.5	B3LYP/6-311G(d,p)	Methanol
**1′*S* -3D**	-915.7340	-574631.77	13.3	B3LYP/6-311G(d,p)	Methanol
**1′*S* -3E**	-915.73397	-574631.74	12.6	B3LYP/6-311G(d,p)	Methanol
**1′*S* -3F**	-915.7339	-574631.74	12.6	B3LYP/6-311G(d,p)	Methanol
**1′*S* -3G**	-915.7319	-574630.48	1.5	B3LYP/6-311G(d,p)	Methanol
**1′*S* -3H**	-915.7319	-574630.46	1.4	B3LYP/6-311G(d,p)	Methanol

Figure S50. Gibbs free energy and equilibrium populations of low-energy conformers of **4** in ECD calculations.

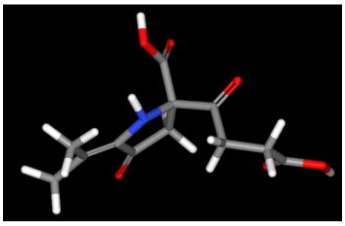

(2*R*-**3A**)

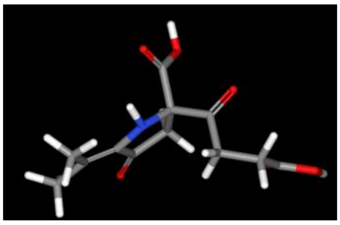

(2*R*-**3B**)

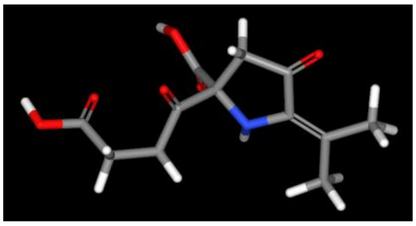

(2*R*-**3C**)

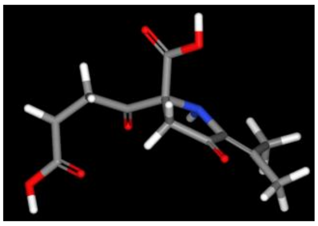

(2*R*-**3D**)

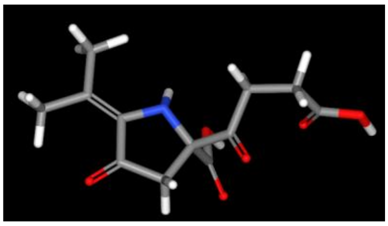

(2*R*-**3E**)

Conformers	Energy (Hartree)	Energy (kcal/mol)	Population (%)	Theory level	Solvent
2*R*-**3A**	-972.7126	-610386.38	33.2	B3LYP/6-311G(d,p)	Methanol
2*R* -**3B**	-972.7123	-610386.19	24.1	B3LYP/6-311G(d,p)	Methanol
2*R* -**3C**	-972.7123	-610386.19	24.1	B3LYP/6-311G(d,p)	Methanol
2*R* -**3D**	-972.7119	-610385.97	16.6	B3LYP/6-311G(d,p)	Methanol
2*R* -**3E**	-972.7096	-610384.50	1.4	B3LYP/6-311G(d,p)	Methanol

Figure S51. UV spectrum of compounds **1-4**.

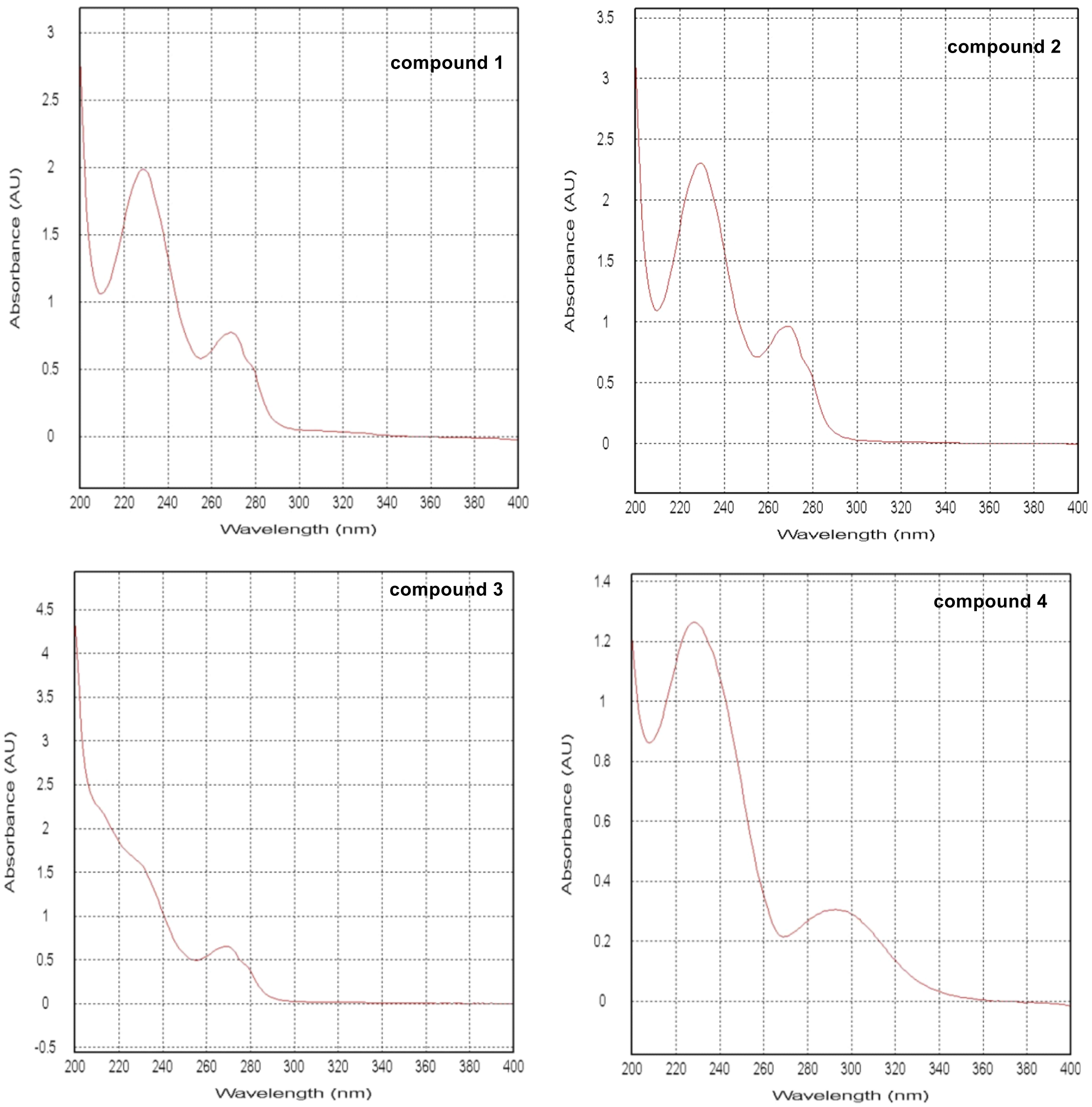

Figure S52. Inhibitory effects of compounds **1**–**22** (1 μM) on LPS-induced nitrite production in BV-2.

**NO.**	**inhibition rate(1uM)**	**NO.**	**inhibition rate(1uM)**
V	100 ± 1.6	**11**	30.7 ± 4.8
LPS	0 ± 1.6	**12**	12.0 ± 1.0
**1**	6.7 ± 2.6	**13**	32.9 ± 1.6
**2**	0.8 ± 0.9	**14**	21.5 ± 3.0
**3**	28.8 ± 1.9	**15**	20.6 ± 2.2
**4**	10.8 ± 5.9	**16**	22.2 ± 2.3
**5**	16.8 ± 2.6	**17**	0 ± 1.9
**6**	15.8 ± 3.3	**18**	15.5 ± 2.8
**7**	-1.9 ± 1.0	**19**	26.9 ± 3.2
**8**	34.2 ± 1.6	**20**	-1.0 ± 1.2
**9**	7.9 ± 2.6	**21**	38.6 ± 2.1
**10**	14.2 ± 2.6	**22**	58.2 ± 2.6
